# Adjuvant and neoadjuvant breast cancer treatments: A systematic review of their effects on mortality

**DOI:** 10.1016/j.ctrv.2022.102375

**Published:** 2022-04

**Authors:** Amanda J. Kerr, David Dodwell, Paul McGale, Francesca Holt, Fran Duane, Gurdeep Mannu, Sarah C. Darby, Carolyn W. Taylor

**Affiliations:** a Nuffield Department of Population Health, University of Oxford, Oxford, UK; b St Luke’s Radiation Oncology Network, St. James’s Hospital, Dublin, Ireland

**Keywords:** Breast cancer, Adjuvant treatments, Neoadjuvant treatments, Treatment benefits, Treatment harms

## Abstract

**Background:**

Adjuvant and neoadjuvant breast cancer treatments can reduce breast cancer mortality but may increase mortality from other causes. Information regarding treatment benefits and risks is scattered widely through the literature. To inform clinical practice we collated and reviewed the highest quality evidence.

**Methods:**

Guidelines were searched to identify adjuvant or neoadjuvant treatment options recommended in early invasive breast cancer. For each option, systematic literature searches identified the highest-ranking evidence. For radiotherapy risks, searches for dose–response relationships and modern organ doses were also undertaken.

**Results:**

Treatment options recommended in the USA and elsewhere included chemotherapy (anthracycline, taxane, platinum, capecitabine), anti-human epidermal growth factor 2 therapy (trastuzumab, pertuzumab, trastuzumab emtansine, neratinib), endocrine therapy (tamoxifen, aromatase inhibitor, ovarian ablation/suppression) and bisphosphonates. Radiotherapy options were after breast conserving surgery (whole breast, partial breast, tumour bed boost, regional nodes) and after mastectomy (chest wall, regional nodes).

Treatment options were supported by randomised evidence, including > 10,000 women for eight treatment comparisons, 1,000–10,000 for fifteen and < 1,000 for one. Most treatment comparisons reduced breast cancer mortality or recurrence by 10–25%, with no increase in non-breast-cancer death.

Anthracycline chemotherapy and radiotherapy increased overall non-breast-cancer mortality. Anthracycline risk was from heart disease and leukaemia. Radiation-risks were mainly from heart disease, lung cancer and oesophageal cancer, and increased with increasing heart, lung and oesophagus radiation doses respectively. Taxanes increased leukaemia risk.

**Conclusions:**

These benefits and risks inform treatment decisions for individuals and recommendations for groups of women.

## Highlights

• Clinical guidelines recommend > 20 adjuvant or neoadjuvant breast cancer options.
• Randomised data on 1000–10,000 women are available for most options.
• Breast cancer mortality or recurrence was reduced by 10–25% for most options.
• Anthracycline chemotherapy and radiotherapy increased non-breast-cancer mortality.
• Radiotherapy-related risks increased with increasing organ dose.

## Introduction

In early invasive breast cancer, neoadjuvant treatments may be recommended before surgery and adjuvant treatments recommended after surgery. These treatments can reduce breast cancer recurrence and mortality, but may increase the risk of death from some other diseases. The evidence on benefits and risks of these treatments is not static but accumulates continuously and is scattered throughout the literature. Therefore, an up-to-date summary is needed for clinical training and to inform treatment decisions in the clinic today.

The highest quality evidence regarding the causal effect of treatment usually arises from a *meta*-analysis of randomised trials or, occasionally, from a single randomised trial [1], [2]. The measure of the causal effect of a treatment that has proved most useful is the rate ratio (RR). The RR is the rate at which a particular endpoint occurs in women allocated to one specific treatment option divided by the corresponding rate in women allocated to a different treatment option, but for whom all other aspects of care are identical. It is important to be aware that RRs compare treatments on a proportional scale, so if the RR for treatment A *versus* treatment B is 2, then the rate at which events are occurring among patients receiving treatment A is double the rate among patients receiving treatment B. It has been observed that in most scenarios RRs are remarkably stable across different groups of patients. This is in contrast to the absolute differences in rates, i.e. the rate for treatment A minus the rate for treatment B, which often vary substantially across different trials, across patients diagnosed in different calendar years, and across groups of patients with different characteristics within a single trial [3], [4].

RRs can be calculated for overall mortality, for mortality from specific causes, and for non-fatal endpoints eg cancer recurrence. For breast cancer mortality, RRs can be used to compare the proportional benefits of different treatments and can inform commissioning of treatments. RRs are also a fundamental component in decision-making at the individual patient level, and several breast cancer decision aids use them in conjunction with mortality rates from regional or national mortality data to provide quantitative estimates of the absolute breast cancer mortality benefits of systemic therapies for individual women. These are widely used in clinics and multidisciplinary team meetings throughout the world [5], [6]

At the present time, decision aids do not provide direct quantitative estimates of the risks of systemic therapies or of the benefits and risks of radiotherapy, although they are referred to in guidelines [7], [8], [9], [10], [11], [12]. The opportunity to fill this gap may arise in the near future, as several new breast cancer decision aids are under development, and updates of existing aids are planned [5], [13], [14].

The benefits and risks of most cancer treatments vary according to dose. For systemic therapy, a few standard regimens and doses are usually used in which each patient receives a similar dose per unit surface area (mg/m^2^) for chemotherapy, or a similar total dose for anti-human epidermal growth factor 2 (HER2) therapies, endocrine therapies and bisphosphonates. RRs from *meta*-analyses of randomised data that assess benefits and risks of these standard systemic therapy regimens are likely to apply to women receiving the same regimens today. For radiotherapy, there is usually little variation in biologically effective doses delivered to the target regions (breast, chest wall and lymph nodes) [15]. However, breast cancer mortality RRs for radiotherapy are unusual in that they vary according to the surgery that a woman has received, her tumour characteristics and the regions irradiated, so different RRs apply in different scenarios. For example, radiotherapy following mastectomy reduces breast cancer mortality substantially in women with positive lymph nodes but not in women with negative nodes [16].

When considering the risks of radiotherapy, the distribution of radiation dose within the patient needs to be considered. Randomised trials have identified heart disease, lung cancer and oesophageal cancer as the main diseases where breast cancer radiotherapy can increase mortality risks. They have also shown that the increased risks are likely to last for many decades after exposure [17]. However, the RRs obtained in the trials are unlikely to be directly relevant for patients being treated today. This is because the radiation-related risk of these diseases depends on the incidental radiation doses received by the heart, lung and oesophagus respectively [17], [18], [19]. Doses to these organs from typical modern radiotherapy are usually lower than for women irradiated in the past, as radiotherapy can now be delivered more precisely. In addition, organ doses from modern radiotherapy vary substantially according to the regimen used [15], [20], [21]. A further complication is that information on incidental heart, lung and oesophagus doses is unavailable in most of the randomised trials of radiotherapy carried out in the past. Therefore the main source of useful information on the magnitude of the radiation risks has proved to be carefully designed observational studies of individuals for whom the relevant organ has been exposed to radiation at a range of doses. These individuals were then followed over several decades to estimate the rate at which they developed or died from the disease in question. These studies have enabled dose–response relationships to be derived in the form of estimated increases in the RR per gray organ dose (Gy) for heart disease, lung cancer and oesophagus cancer. The increase in RR per Gy can then be combined with typical modern organ doses in Gy to provide estimates of RRs from typical modern radiotherapy regimens.

We present a systematic review of the information needed to estimate proportional benefits and risks of modern adjuvant and neoadjuvant treatment options recommended in current clinical guidelines for early breast cancer. For each treatment option, the literature was searched for randomised evidence and the highest-ranking study was identified. RRs for breast cancer and non-breast-cancer mortality were then collated and summarised.

## Methods

**Fig. 1:**
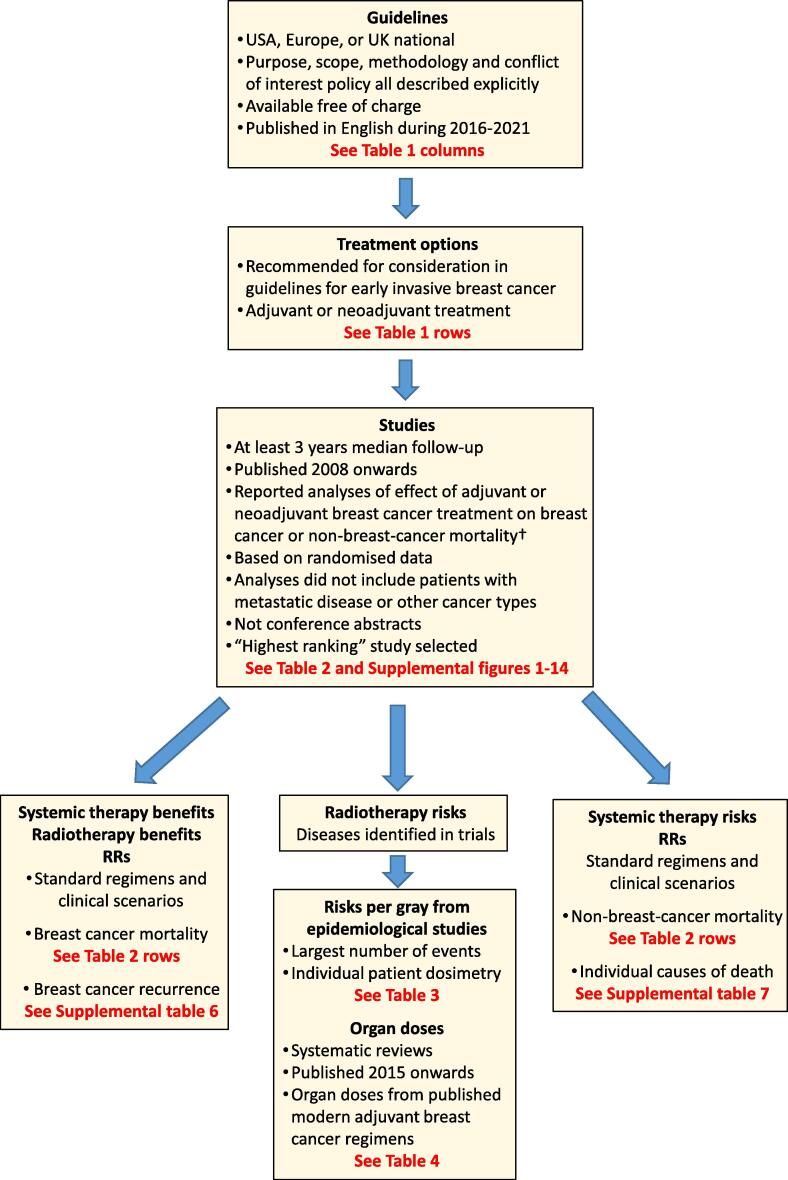
Flowchart for study with criteria applied at each stage. † If no eligible trial was found, the trial referenced in the guidelines was used. Abbreviations: RR = rate ratio.

The methods used in the present study are explained below and summarised in Fig. 1.

### Guidelines

US, European and UK national breast cancer guidelines were identified. Guidelines were included if their purpose, scope, methodology and conflict of interest policy were all clearly stated, and they were freely available and published in English during 2016 to 2021. Emergency guidelines recommending temporary changes to practice during the COVID-19 pandemic were not included, nor were guidelines specifically for hereditary or non-invasive breast cancer.

### Treatment options

Adjuvant and neoadjuvant treatment options recommended in guidelines for consideration in early invasive breast cancer were listed. Early invasive breast cancer was defined as cancer that has not spread beyond the breast or axillary lymph nodes. This included stages I, IIA, IIB and IIIA breast cancers, and excluded carcinoma in situ [22]. Adjuvant and neoadjuvant treatments were those intended to reduce risks of cancer recurrence and/or death. Adjuvant treatment was delivered after curative-intent surgery, and neoadjuvant treatment before curative-intent surgery.

### Searches

For each treatment option, systematic literature searches were conducted to identify the highest-ranking evidence of its effects on mortality. New breast cancer treatments are sometimes recommended in the USA before they are endorsed by European or UK guidelines. Searches were not performed for treatments not yet recommended outside the USA.

Database searches were conducted for *meta*-analyses of trials of each treatment option compared with a less intensive treatment option (Supplemental Table 2a; Supplemental Figs. 1-14) [23]. Eligible studies compared treatment effects on breast cancer or non-breast-cancer mortality, included at least 3 years median follow-up and were published 2008 and onwards, as earlier studies would inevitably assess the effects of older treatment regimens. Non-randomised studies comparing different treatments were excluded because they may provide misleading estimates of treatment effects [24]. We also excluded studies of patients with metastatic disease, or other cancer types. Conference abstracts were excluded since they are not usually peer-reviewed; and most have insufficient detail on methods for study ranking.

If a search identified more than one eligible *meta*-analysis, they were ranked to identify the one providing the strongest evidence for each treatment option using the following criteria:

1. Individual patient data *meta*-analysis including all relevant randomised trials, both published and unpublished.
2. Individual patient data *meta*-analysis omitting some relevant randomised trials.
3. Published data *meta*-analysis including all relevant randomised trials.
4. Published data *meta*-analysis omitting some relevant randomised trials.

For treatment options with no eligible *meta*-analysis, additional database searches were conducted for individual randomised trials. If more than one randomised trial was found, the trial with the largest number of women randomised was used. If no eligible trial was found, the largest meta analysis or trial referenced in the guidelines was used.

### Rate ratios

For each selected *meta*-analysis or trial, RRs for breast cancer mortality, non-breast-cancer mortality and individual causes of death were extracted. For chemotherapy, anti-HER2 therapy, endocrine therapy and bisphosphonates, RRs were extracted for standard dose regimens recommended in breast cancer guidelines (Supplemental Table 1). For radiotherapy, RRs were extracted for clinical scenarios quoted in guidelines. Where RRs were unavailable, hazard ratios or odds ratios were used or risk ratios were calculated (Supplemental Table 3). If guidelines recommended treatment options that were not yet reported to reduce breast cancer mortality, RRs for breast cancer recurrence were also extracted.

RRs were extracted by two oncologists (CT and DD) and checked by two other oncologists and a breast surgeon (FH, FD and GM). Discrepancies were resolved by consensus. For each endpoint the time period studied was extracted, defined as the time period covered by the RRs, as was the number of women on which the RR was based. RRs which differed from 1.00 with a two-sided p-value of ≤ 0.05 were regarded as significantly increased or decreased. 95% confidence intervals (CIs) are shown.

### Risks of radiotherapy

For each individual cause of death with a significantly raised RR from radiotherapy, additional searches were performed for relevant dose–response relationships (Supplemental Table 2b). The dose–response relationship with the largest number of events based on individual patient radiation dosimetry was selected, and details extracted. For each dose–response relationship, a search was performed for systematic reviews of radiation doses to the relevant organs from typical modern radiotherapy regimens (Supplemental Table 2b). Eligible reviews were those that included organ doses from all available published modern early breast cancer regimens and were published 2015 onwards.

## Results

### Guidelines

Guidelines from six organisations were used to extract treatment options (Table 1). USA guidelines included the National Comprehensive Cancer Network (NCCN) [25], American Society of Clinical Oncology (ASCO) [26], [27], [28], [29], [30] and American Society for Radiation Oncology (ASTRO) [8], [9], [10]. European guidelines included the European Society for Medical Oncology (ESMO) [11] and St Gallen [31], [32]. UK guidelines were produced by the National Institute for Health and Care Excellence (NICE) [7], [33], [34], [35]. Two organisations, European Society for Radiotherapy and Oncology (ESTRO) and the Royal College of Radiologists (RCR), were not used to identify options because their guidance informs technical aspects of the radiotherapy options included in ESMO and NICE.

### Treatment options

Treatment types recommended for consideration in adjuvant or neoadjuvant early breast cancer included chemotherapy, anti-HER2 therapy, other targeted therapy, endocrine therapy, bisphosphonates and radiotherapy (Table 1). Within each type, there were several treatment options. Most options were recommended in all relevant guidelines, although some entered different guidelines at different times (Supplemental Table 4). Two options, pembrolizumab and abemaciclib were recommended only in the USA. European and UK guidelines either did not mention them, or stated that insufficient evidence was available to recommend them.

For chemotherapy, USA guidelines listed categories and individual regimens whereas European guidelines usually listed just categories: anthracycline-based, anthracycline + taxane, taxane-based and platinum-based (Supplemental Table 1). Most chemotherapy options were recommended for use in either the adjuvant or neoadjuvant setting. Neoadjuvant delivery was recommended for women at higher risk of breast cancer recurrence, who had HER2 positive or ER negative cancers, and adjuvant delivery was recommended for other women. For two chemotherapy types, the recommended timing of delivery was specified as adjuvant for capecitabine and neoadjuvant for platinum-based chemotherapy.

Recommended anti-HER2 therapies in HER2 positive cancer were trastuzumab, pertuzumab, trastuzumab emtansine and neratinib, in neoadjuvant or adjuvant settings. For other targeted therapies, the recommended timing of delivery was specified as neoadjuvant for pembrolizumab and adjuvant for abemaciclib.

Endocrine therapies in oestrogen receptor (ER) positive disease were tamoxifen and aromatase inhibitors (AIs) given with varying durations and sequences and, in pre-menopausal women, ovarian suppression or ablation. Endocrine therapy in ER positive disease, was usually recommended after surgery, but neoadjuvant delivery could be given for women at low risk of recurrence (Table 1). Bisphosphonates were recommended in postmenopausal women in the adjuvant setting.

**Table 1:** Guidelines and treatment options in adjuvant and neoadjuvant breast cancer during 2016–2021.

Abbreviations NCCN National Comprehensive Cancer Network; ASCO American Society of Clinical Oncology; ASTRO American Society for Radiation Oncology; Bisphos Bisphosphonates; ESMO European Society of Medical Oncology; St Gallen St Gallen International Consensus Guidelines; NICE National Institute for Health and Care Excellence; P page; HER2 human epidermal growth factor receptor 2; ER oestrogen receptor; PR progesterone receptor; yr years; OS/OA ovarian suppression or ablation; AI aromatase inhibitors; RT radiotherapy

*Patient groups are listed only if recommendations are similar in all relevant guidelines

†Section numbers refer to NICE Guideline for all rows apart from “Appraisal” which refer to NICE Technology Appraisal Guidance

‡NCCN and ASCO list chemotherapy regimens. ESMO, St Gallen and NICE list general categories only (see Supplemental Table 1 for further details).

§NCCN, ESMO and St Gallen: HER2-/ER- disease only. ASCO: HER2- disease with any ER-status

¶Pembrolizumab was recommended in NCCN 2022 guidelines. It was stated as “not recommended” in St Gallen 2021 and ASCO 2021. It is currently under consideration by NICE (2022). It was not mentioned in ESMO. It was only recommended in USA guidelines therefore it is not included in subsequent tables.

**High risk was defined as: ≥4 positive nodes, or 1–3 positive nodes with one or more of the following: Grade 3, tumour size ≥ 5 cm, Ki-67 score ≥ 20%.

††Abemaciclib was recommended by ASCO 2022 and mentioned as an option in NCCN 2022. St Gallen 2021 stated “the panel was divided on whether to endorse abemaciclib adjuvant therapy” and “longer term follow-up from trials is awaited to settle this question”. Abemaciclib was not mentioned in ESMO and NICE. It was only recommended in USA guidelines therefore it is not included in subsequent tables.

‡‡NCCN and ASCO: Inoperable cancer, or operable cancer if high risk HER2+ or triple negative or to reduce the extent of surgery or patients in whom surgery may be delayed. NICE: High risk HER2+ or ER- or to reduce tumour size. ESMO and St Gallen: Inoperable cancer, or operable cancer if high risk HER2+ or triple negative or to reduce the extent of surgery.

Adjuvant radiotherapy after breast conserving surgery was recommended in most women. This involved either whole breast radiotherapy, sometimes with an additional radiation boost to the tumour bed, or partial breast radiotherapy. Radiotherapy recommended after mastectomy included the chest wall. Radiotherapy to the regional nodes may be recommended after either breast conserving surgery or mastectomy.

### Treatment comparisons: Systemic therapies

Treatment comparisons in randomised trials provided direct evidence for most treatment options (Table 2, Supplemental table 5). Searches were not performed for pembrolizumab and abemaciclib since they were only recommended in the USA.

**Table 2:** Studies and rate ratios for breast cancer and non-breast-cancer mortality from randomised trials comparing different adjuvant or neoadjuvant breast cancer treatments (see also Supplemental Tables 5–7).

Abbreviations: RR rate ratio; CI confidence interval; *vs versus*; HER2 human epidermal growth factor 2; ER oestrogen receptor; PR progesterone receptor; path CR pathological complete response; AI aromatase inhibitor; RT radiotherapy

*The time period following diagnosis that RRs relate to. In most studies, this starts soon after time of diagnosis, as randomisation took place soon after diagnosis. For studies where randomisation did not take place until several years after diagnosis, both time from diagnosis to randomisation and time studied following randomisation are given in footnotes.

†The number of women was the same for assessment of breast cancer mortality and non-breast-cancer mortality unless indicated.

‡ Anthracycline breast cancer mortality rate ratio is for four or more cycles of any anthracycline regimen e.g. 4AC (doxorubicin and cyclophosphamide) *versus* no chemotherapy. The non-breast-cancer mortality rate ratio is for any anthracycline chemotherapy *versus* no chemotherapy.

§Taxane + anthracycline *vs* anthracycline breast cancer mortality rate ratio is for the addition of four taxane cycles to anthracycline-based chemotherapy (usually 4AC). An estimate of the RR for taxane + anthracycline *vs* no chemotherapy can be derived by multiplying the RRs for anthracycline *vs* nil and taxane + anthracycline *vs* nil, i.e. 0.79 × 0.86 = 0.68 (95% CI 0.59–0.77). The non-breast-cancer mortality rate ratio is for taxane + anthracycline *vs* the same or more anthracycline-based non-taxane chemotherapy.

¶For capecitabine *versus* not, a reduction in overall mortality was reported (RR 0.59, 95% CI 0.39–0.90, P = 0.01) so it is likely that capecitabine did, reduce breast cancer mortality, although this was not reported specifically.

**Rate ratio not published. Values shown are risk ratios calculated from published data, see Supplemental Table 3 for details.

††Meta-analysis of 4 published trials. All women received 5 years of tamoxifen before randomisation. Median follow-up after randomisation varied from 4.2 to 7.6 years. Reported measure of reduction in breast cancer mortality is odds ratio.

‡‡In the two largest trials, the RRs for non-breast-cancer mortality were 0.99 (95% CI 0.89–1.10) (ATLAS) [55] and 0.94 (0.82–1.07) (aTTom) [56].

§§Treatments diverged 2–3 years after diagnosis. Time-period studied is 8 years starting at 2 years after diagnosis.

¶¶Women randomised after around 5 years of AI preceded by 5 years of tamoxifen. Time-period studied is 10 years starting at randomisation.

***Women received about 5 years of AI or of tamoxifen → AI before randomisation. Time-period studied is 7 years starting at randomisation.

†††Includes women of all ages

‡‡‡76% of patients had invasive breast cancer and 24% had ductal carcinoma in situ.

§§§ Includes all trials of RT *versus* no RT and also trials of RT *versus* more extensive surgery.

Searches yielded eligible *meta*-analyses for: anthracycline, taxane + anthracycline, trastuzumab, timing of systemic therapy, 5 years of tamoxifen and extended tamoxifen (ER positive cancer); AI *versus* tamoxifen (in premenopausal women with ovarian suppression or ablation and in postmenopausal women, both with ER positive cancer) and tamoxifen/AI *versus* tamoxifen alone (postmenopausal women with ER positive cancer); and bisphosphonates (postmenopausal women) (Supplemental Figs. 1,2,5a,8–11,13) [4], [38], [42], [43], [44], [46], [47], [51].

For treatment comparisons with no eligible *meta*-analysis, searches yielded eligible randomised trials for: pertuzumab and trastuzumab emtansine in HER2 positive cancer (Supplemental Figs. 5c,6b) [39], [40]. In endocrine therapy for pre-menopausal women with ER positive cancer there was an eligible trial for ovarian suppression, (Supplemental Fig. 12b) [45]. In endocrine therapy for post-menopausal women with ER positive cancer there was an eligible trial for AI *versus* not after 5 years of tamoxifen [48]. For extended AI, two randomised trials with differing designs were included because guidelines differed in their recommendations as to the optimal AI duration (Supplemental Fig. 11) [49], [50].

For neoadjuvant platinum in triple negative cancer, for capecitabine, in HER2 negative residual cancer after neoadjuvant chemotherapy, and for neratinib in HER2 positive cancer, no trials reported breast cancer mortality. Therefore the effects of platinum on pathological complete response and of capecitabine and neratinib on breast cancer recurrence were extracted from the studies cited in the guidelines (Supplemental Figs. 3a, 4b, 7b, Supplemental Table 6) [36], [37], [41].

The number of women randomised in treatment comparisons providing direct evidence regarding the efficacy of systemic treatment was>10,000 for six comparisons, between 1,000 and 10,000 for eleven comparisons and<1000 for one comparison (Table 2).

For taxanes, only indirect evidence was available. There were no trials of taxane-based treatment without inclusion of an anthracycline, *versus* no chemotherapy. Also, few women were randomised to taxane + anthracycline *versus* no chemotherapy. Instead the overall effect of taxane + anthracycline can be assessed by multiplying the RRs for taxane + anthracycline *versus* anthracycline and anthracycline *versus* no chemotherapy.

### Treatment comparisons: Radiotherapy

The guidelines recommended a total of five radiotherapy options (Table 1). Treatment comparisons in randomised trials provided direct evidence for all of these options (Table 2, Supplemental table 5).

Searches yielded eligible *meta*-analyses for radiotherapy after breast conserving surgery and radiotherapy after mastectomy (Supplemental Fig. 14a) [3], [16]. For treatment comparisons with no eligible *meta*-analyses, searches identified one eligible randomised trial for each of regional node, partial breast and tumour bed boost radiotherapy (Supplemental Fig. 14b-d) [52], [53], [54]. The number of women randomised in treatment comparisons of the efficacy of radiotherapy was >10,000 for one comparison, and between 1,000 and 10,000 for four comparisons (Table 2).

Evidence identifying causes of non-breast-cancer mortality affected by radiotherapy came from a *meta*-analysis including over 40,000 women in randomised trials of radiotherapy *versus* not following any surgery, and with any targets, and trials of radiotherapy *versus* more extensive surgery [17].

### Rate ratios

**Fig. 2:**
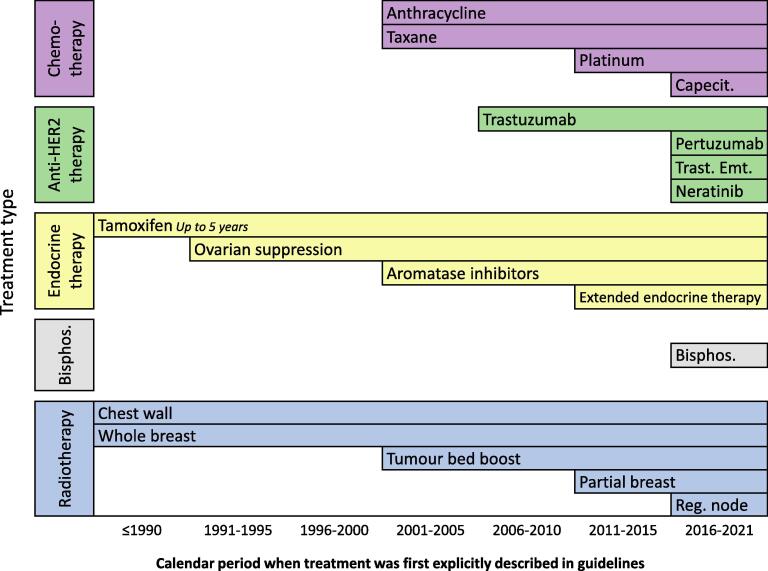
Calendar period when early breast cancer treatments were first explicitly described in clinical guidelines. For further details, see Supplemental table 4. Abbreviations: Reg. node = regional node, Bisphos. = bisphosphonates, Trast. Emt. = trastuzumab emtansine, Capecit = capecitabine.

For treatments first recommended in 1990 or earlier (Fig. 2) (up to 5 years of tamoxifen, whole breast RT, chest wall RT), trials had lengthy follow-up and RRs compared treatments over a period of at least 15 years following diagnosis (Table 2). For newer treatments first recommended after 2015 (capecitabine, pertuzumab, trastuzumab emtansine, neratinib, bisphosphonates, regional node radiotherapy), follow-up was shorter and most RRs compared treatments over <10 years following diagnosis. For other treatments, first recommended during 1991 to 2015, the length of follow-up was variable and RRs compared treatments over < 1 to 20 years after diagnosis.

### Benefits in breast cancer mortality

For eight systemic therapy comparisons (anthracycline *versus* no chemotherapy; taxane + anthracycline *versus* anthracycline; trastuzumab *versus* not; 5 years of tamoxifen *versus* not; extended tamoxifen ≥ 10 *versus* 5 years; AI *versus* tamoxifen in postmenopausal women; tamoxifen/AI *versus* tamoxifen; and bisphosphonate *versus* not) there were statistically significant reductions in breast cancer mortality, with RRs varying from 0.67 (95% CI 0.61–0.73) to 0.88 (95% CI 0.80–0.97) (Table 2). Eight of the ten remaining systemic therapy options significantly reduced breast cancer recurrence (Supplemental Table 6). Neoadjuvant platinum chemotherapy increased pathological complete response rate, but trials are not mature enough to assess its effect on breast cancer mortality. A meta analysis assessed the effect of timing of chemotherapy relative to surgery. It included all trials randomising women to the same chemotherapy given neoadjuvantly *versus* adjuvantly. Delivery of chemotherapy neoadjuvantly had no significant effect on breast cancer mortality. It did however increase local breast cancer recurrence (RR 1.37, 95% CI 1.17–1.61) (Supplemental table 6) [42].

For five treatment comparisons (pertuzumab *versus* not; trastuzumab emtansine *versus* trastuzumab; ovarian suppression *versus* not (both with tamoxifen); AI *versus* not (both after 5 years of tamoxifen); and extended AI), RRs for breast cancer mortality were less than one, but the 95% CIs included one. For capecitabine *versus* not, a reduction in overall mortality was reported (RR 0.59, 95% CI 0.39–0.90, P = 0.01) so it is likely that it did, in fact, reduce breast cancer mortality, although this was not reported specifically. For platinum chemotherapy *versus* other chemotherapy and for neratinib *versus* not, breast cancer mortality by treatment allocation has not yet been reported.

In radiotherapy, there were significant reductions in breast cancer mortality for radiotherapy after breast conserving surgery (RR = 0.82, 95% CI 0.75–0.90), radiotherapy after mastectomy and axillary dissection in node positive disease (RR = 0.84, 95% CI 0.76–0.94) and regional node radiotherapy (RR = 0.81, 95% CI 0.74–0.94).

The other two radiotherapy options — partial breast *versus* whole breast radiotherapy and tumour bed boost after whole breast radiotherapy — did not increase or reduce breast cancer mortality significantly (Table 2). Partial breast radiotherapy was recommended in guidelines due to the theoretical benefits of irradiating less tissue than whole breast radiotherapy, and for its potential to reduce overall treatment time [7], [9], [11]. It did not significantly affect recurrence at any site or ipsilateral breast tumour recurrence [52]. Tumour bed boost was recommended in addition to whole breast radiotherapy because it has been shown to reduce ipsilateral breast tumour recurrence [10], [11] (Supplemental Table 6).

### Risks of non-breast-cancer mortality and individual causes of death

For systemic therapies, anthracycline chemotherapy significantly increased non-breast-cancer mortality (RR = 1.20, 95% CI 1.00–1.43) (Table 2), with significant increases in mortality from heart disease and acute myeloid leukaemia (heart disease RR = 1.61, 95% CI 1.00–2.22 and acute myeloid leukaemia 8/4754 *versus* 0/4733 deaths, p = 0.004) (Supplemental Tables 3, 7). The cumulative dose of anthracycline in these trials ranged from 240 to 360 mg/m^2^ doxorubicin or 400–800 mg/m^2^ epirubicin [4]. For taxane chemotherapy, the RR for leukaemia was 11.00, 95% CI 1.42–85.17, based on 10/22,128 leukaemias in women randomised to taxane + anthracycline and 1/22,123 in women randomised to anthracycline. Leukaemia is rare, and there were too few events for this excess to increase non-breast cancer mortality (RR = 0.99, 95% CI 0.83–1.15). AI *versus* tamoxifen in premenopausal women increased mortality from cancers other than breast based on 22/3528 cancer deaths in women randomised to AI *versus* 10/3502 in women randomised to tamoxifen, but with no significant increase in overall non-breast-cancer mortality (RR 1.30, 95% CI 0.75–2.25) [46]. There were no reports of increased mortality from non-breast-cancer causes for other systemic therapies.

For radiotherapy, a *meta*-analysis of all available randomised trials comparing radiotherapy *versus* no radiotherapy yielded an overall non-breast-cancer mortality RR of 1.15 (95% CI 1.09–1.22) [17] (Table 2). This increase was mainly due to heart disease (RR 1.30, 95% CI 1.15–1.46), lung cancer (RR 1.64, 95% CI 1.22–2.21), oesophageal cancer (RR = 2.51, 95% CI 1.08–5.72) and thromboembolism (RR = 2.10, 95% CI 1.11–3.90) (Supplemental Table 7).

### Radiotherapy risks per unit dose

For heart disease the dose–response relationship based on the largest number of cardiac events with individual patient dosimetry, included 963 cases and the RR for major coronary events increased by 7.4% per Gy (95% CI 2.9–14.5) [18] (Table 3, Supplemental Table 8). For lung cancer there were five dose–response relationships based on individual patient or trial-level dosimetry. These were combined in a published data *meta*-analysis [17] and the RR per Gy per mean whole lung dose was 11% (95% CI 6–19). For oesophageal cancer, the risk increased by 7.1% per Gy (95% CI 1.8–20.6) median oesophagus dose [19]. For thromboembolism, no dose–response relationships were available and the mechanism of radiation-related disease remains unknown.

**Table 3:** Dose-response relationships for individual causes of non-breast-cancer mortality that are significantly increased by radiotherapy.

**Endpoint**	**Organ**	**Dose measure**	**Number of** **events**	**Percentage increase** in RR per Gy*	**Reference**†	**Table/figure in reference**
Major coronary events	Whole heart	Mean	963	7.4% (95% CI 2.9–14.5)	Darby 2013^18^	Fig. 1
Lung cancer	Lung‡	Mean	475 all studies combined	11% (95% CI 6–19)	EBCTCG 2017^17^	Fig. S8
Oesophageal cancer	Whole oesophagus	Median	156	7.1% (95% CI 1.8–20.6)§	Journy 2020^19^	Table 2

Abbreviations: RR rate ratio; Gy gray; CI confidence interval

* i.e. excess RR per Gy (lung cancer and major coronary events) or excess odds ratio per Gy (oesophageal cancer). Models are of the form B_s_(1 + KX/100) where S denotes a group, or stratum, of individuals for whom the rate at which the endpoint occurs in the absence of radiation exposure is likely to be similar. B_s_ is the rate at which the endpoint occurs in that stratum in the absence of radiotherapy, X is the dose measure in Gy and K is the percentage increase in the rate ratio or the odds ratio per Gy.

† See also Supplemental Table 8.

‡ Based on published data *meta*-analysis of five studies where doses were allocated to individuals based on trial-level or individual patient doses. Organ doses were for both lungs combined in one study, ipsilateral lung in two studies and location of second cancer in two studies.

§ The dose–response relationship based on whole oesophagus dose is listed because it is based on median oesophagus dose, which is assessable for patients being considered for breast cancer radiotherapy.

### Radiotherapy risks and organ doses

Four systematic reviews were identified reporting typical regimen-specific organ doses, ie the average of the mean organ doses measured in CT plans for that regimen. Some regimen-specific doses were based on radiotherapy plans from multiple patients, others were based on a single radiotherapy plan.

Two systematic reviews of heart radiation doses from modern breast cancer radiotherapy were identified (Table 4). The first included all regimens published during 2003–2013 which reported mean heart dose from breast cancer radiotherapy [15]. There were 45 right and 357 left radiotherapy regimens in 167 studies from 28 countries. A second systematic review included heart doses from 32 right and 196 left regimens published in 99 studies during 2014–2017 [57]. For lung doses, one systematic review included mean whole lung doses (right and left lungs combined) from 218 regimens in 88 studies published during 2010–2015 [20]. For whole oesophagus doses, one systematic review included mean oesophagus doses from 89 regimens in 33 studies published during 2010–2020 [21]. In these reviews, the main determinants of heart, lung and oesophagus dose were regions irradiated and techniques used. For all these organs the range of doses was substantial and for many regimens, organ doses of over 10 Gy had been reported.

**Table 4:** Typical modern radiotherapy organ doses for heart, lung and oesophagus in systematic reviews of breast cancer radiotherapy published during 2015–2020.

**Organ**	**Years of radiotherapy**	**Number regimens/laterality of cancer**	**Regions irradiated**	Organ doses* Average/range		**Reference**	**Table/Figure in reference**
Whole heart	2003–2013	45 right 357 left	Right, all regimens Left without IMN Left + IMN	3.3 4.2 8.4	0.4–21.6 <0.1–23.0 0.7–28.6	Taylor 2015^15^	Table 1 Fig. 3
							
Whole heart	2014–2017	32 right 196 left	Right whole breast Left whole breast	1.9 3.6	0.2–8.8 0.1–18.7	Drost 2018^57^	Table 1
							
Both lungs combined	2010–2015	218 right + left combined	Partial breast Breast/chest wall Breast/chest wall + axilla/SCF Breast/chest wall + axilla/SCF + IMN	1.6 5.6 7.3 8.6	0.3–5.1 0.2–13.1 2.2–12.1 3.0–12.1	Aznar 2018^20^	Fig. E4
							
Whole oesophagus	2010–2020	89 right + left combined	Partial breast Breast/chest wall Breast/chest wall + SCF/axilla/IMN	0.2 1.8 11.4	0.1–0.4 0.1–10.4 1.1–29.3	Duane 2021^21^	Fig. 3

Abbreviations: SCF supraclavicular fossa; IMN internal mammary node

* Average and range of reported regimen-specific doses for radiotherapy to different target regions. Each regimen-specific dose is the average of the mean organ doses in individual patient CT plans for the regimen.

## Discussion

This review of adjuvant and neoadjuvant treatments for early breast cancer provides a resource for clinical practice and training. It summarises the treatment options recommended in USA, European and UK national guidelines, and the benefits and risks of those options compared, in most cases, with an already established standard, less intensive, treatment. The proportional benefits of most treatment options compared with standard treatment were a 10–25% reduction in breast cancer mortality or recurrence, with no overall increase in non-breast-cancer death.

Two treatments, anthracycline chemotherapy and radiotherapy, did increase overall non-breast-cancer death. For anthracycline, the main risks were from heart disease and acute myeloid leukaemia. For individual causes of death, taxanes increased leukaemia risk and aromatase inhibitors in premenopausal women were associated with increased mortality from second cancers, but this was based on few events. The risks from current systemic therapies are likely to be similar to those described here since similar doses of these drugs are used today. Radiation-risks were mainly from heart disease, lung cancer, oesophageal cancer and thromboembolism. The radiation risks from current treatment are likely to be lower than those in the evidence presented here, as organ-specific doses are lower. This review summarises the information needed to estimate proportional radiation-risks from modern radiotherapy.

### Strengths and weaknesses

This is the first published review to summarise systematically the quantitative evidence on the proportional mortality benefits and risks from adjuvant and neoadjuvant breast cancer treatments. It has several strengths.

First, it collates multiple estimates that were previously scattered widely throughout the literature. Most of these estimates were in tables, graphs, footnotes, or in the text of publications and were time-consuming to locate.

Second, wherever possible the same endpoints — breast cancer and non-breast-cancer mortality — were presented for all studies, so the effects of different treatments could be compared. Wherever possible we have presented the results in terms of RRs. However, in one study, only odds ratios were available and so we provided them. For some other studies only numbers of cause-specific deaths were available and for these we calculated and provided the relevant risk ratios. For these trials in which rates are small and the treatments arms being compared have usually been followed for similar lengths of time, both of these approximations are likely to perform well.

Third, our searches were extensive, with inclusive terms. We searched > 13,000 publications in total, with > 100 publications for each treatment option. Therefore we are likely to have identified and assessed all relevant studies.

Fourth, ranking of search results enabled identification of the highest quality estimates, with the least risk of bias and the greatest number of women randomised. Measures of strength of evidence were abstracted, including the number of women randomised, confidence intervals, and the time period studied. For 12 of the 24 treatment comparisons (Table 2) an individual patient data *meta*-analysis was identified including all relevant available randomised trials. Most adjuvant and neoadjuvant breast cancer treatments have modest effects on mortality that can be difficult to quantify in individual trials due to lack of statistical power. However, *meta*-analyses of all available randomised data can enable reliable assessment of treatment effects up to 10 years. Even using the highest quality randomised evidence, there is uncertainty about the effects of treatment beyond this since most trials do not usually follow patients beyond 10 years.

Fifth, the review brings together all evidence types needed to assess quantitatively the proportional risks from modern breast cancer radiotherapy, including *meta*-analyses of randomised data, dose–response relationships and systematic dosimetry reviews. These may be used to estimate risks from modern radiotherapy regimens using typical regimen-specific organ doses. Radiation-risks can also be estimated for individual women, using organ doses from their radiotherapy planning CT-scan. It is not known how radiotherapy causes thromboembolism, so that at present it is difficult to quantify with any confidence the thromboembolic risk from current radiotherapy.

A limitation of our study is that we considered only the mortality benefits and risks of breast cancer treatments, not their effects on breast cancer recurrence or on side-effects that are not usually fatal, such as endometrial cancer after tamoxifen. The exception is the inclusion of more recent trials mentioned in guidelines and relevant to current clinical practice, but which had not reported an improvement in breast cancer mortality at the time of writing. In addition, even using all available randomised evidence, there were few events for reliable assessment of rare side-effects. For example, the risk of acute myeloid leukaemia after taxane chemotherapy was significantly increased in a *meta*-analysis including 44,251 women, but this was based on just 12 events. A recent abstract of a *meta*-analysis of trials comparing taxane + anthracycline *versus* taxane alone reported no increase in leukaemias, although longer follow-up is needed [58].

A further limitation is that the review can inevitably only assess current guidelines and evidence. As existing trials mature, and new trials are reported, guidelines will change, and updated reviews will be needed.

### Clinical implications

The RRs in our review may be used by health care professionals to estimate benefits and risks of adjuvant and neoadjuvant breast cancer treatments for patients today. For breast cancer mortality, RRs can be used to compare the proportional benefits of different treatments and can inform prioritisation of treatments at a national level.

At an individual patient level, the trade off between absolute benefits and risks of treatments is complex. Absolute benefits and risks differ according to patient and tumour characteristics. Estimation of these effects requires data on women in the general population, and it is outside the scope of this review. It was recently addressed in a systematic review of breast cancer decision aids [12]. The RRs in our review may however be used in breast cancer decision aids. First, the list of early breast cancer treatments recommended in current clinical guidelines informs which new treatments may be included when decision aids are updated. For example capecitabine, pertuzumab, trastuzumab emtansine and neratinib, are recommended in clinical guidelines but are not included in current decision aids, so clinicians cannot easily estimate their absolute benefits and risks for individual patients. Second, our summary of the highest-ranking RRs may be used to update current decision aids. Third, our review provides quantitative estimates of the risks from systemic therapy and of the benefits and risks from radiotherapy. These are not currently available in decision aids.

Our review of clinical guidelines illustrates the successive improvements in adjuvant and neoadjuvant breast cancer treatments that have occurred during the past 30 years (Fig. 2). During 2001–2005, taxane and anthracycline chemotherapy were first recommended, as were AIs and tumour bed radiotherapy boost. These were followed by trastuzumab in 2006–2010, platinum chemotherapy, extended tamoxifen and partial breast RT during 2011–2015. Then during 2016–2021 several new anti-HER2 agents were approved for use, as were bisphosphonates and capecitabine, and the role of regional node RT was established. The resulting changes in clinical practice are likely to have reduced breast cancer mortality. Since the 1990s, breast cancer mortality in high income countries has approximately halved, and a considerable part of this is likely to be due to improved treatments [59].

### Future research

Breast cancer practice is likely to continue to improve as the results of more trials become available. Several ongoing trials are investigating treatment de-escalation. The timing of treatments relative to surgery is changing and an increasing number of women now receive neoadjuvant chemotherapy. More information is needed on whether RRs from adjuvant regimens also apply to the same regimens if they are used before surgery. Chemotherapy regimens may also change. A few recent trials have assessed the impact of avoiding anthracycline in chemotherapy regimens, but at present the randomised evidence on this is limited [58].

In the future, new chemotherapy and anti-HER2 therapies may be recommended. For currently recommended treatments, increased follow-up of trials may provide additional information on long-term outcomes. This is particularly relevant to toxicity, which can occur several decades after treatment. As evidence increases, so will the need for accurate up-to-date summaries of it.

## CRediT authorship contribution statement

**Amanda J. Kerr:** Conceptualization, Data curation, Investigation, Methodology, Visualization, Writing – review & editing. **David Dodwell:** Conceptualization, Data curation, Investigation, Methodology, Writing – review & editing. **Paul McGale:** Conceptualization, Methodology, Writing – review & editing. **Francesca Holt:** Data curation, Writing – review & editing. **Fran Duane:** Data curation, Writing – review & editing. **Gurdeep Mannu:** Data curation, Writing – review & editing. **Sarah C. Darby:** Analysis, Conceptualization, Data curation, Investigation, Methodology, Supervision, Visualization, Writing – review & editing. **Carolyn W. Taylor:** Conceptualization, Data curation, Investigation, Methodology, Supervision, Visualization, Writing – original draft.

## Declaration of Competing Interest

The authors declare that they have no known competing financial interests or personal relationships that could have appeared to influence the work reported in this paper.
