# Supplementary material for: Adjuvant and neoadjuvant breast cancer treatments: A systematic review of their effects on mortality
Source: Cancer Treat Rev. Author manuscript; Available in PMC 2022 Jun 18. (PMC9096622; doi:10.1016/j.ctrv.2022.102375)
Supplement: Appendix 1 [file EMS144010-supplement-Appendix_1.pdf]

Adjuvant and neoadjuvant breast cancer treatments:

A systematic review of their effects on mortality

Amanda Kerr, David Dodwell, Paul McGale, Francesca Holt,
Fran Duane, Gurdeep Mannu, Sarah C Darby, Carolyn W Taylor

Supplementary material

Contents

Guidelines and searches		*Page*
Supplemental Table 1	Chemotherapy treatment options recommended in guidelines	4
Supplemental Table 2a	Embase and Medline database searches for rate ratios of the effects of adjuvant and neoadjuvant breast cancer treatments	5
Supplemental Table 2b	Database searches for radiation dose-response relationships and doses from typical modern breast cancer radiotherapy for mortality risks significantly increased by radiotherapy	9

PRISMA Diagrams summarising the process of study identification

Supplemental Figure 1	Anthracycline chemotherapy	10
Supplemental Figure 2	Taxane+anthracycline chemotherapy	11
Supplemental Figure 3	Platinum chemotherapy	12
Supplemental Figure 4	Capecitabine chemotherapy	14
Supplemental Figure 5	Trastuzumab/trastuzumab emtansine	16
Supplemental Figure 6	Pertuzumab	19
Supplemental Figure 7	Neratinib	21
Supplemental Figure 8	Neoadjuvant chemotherapy	23
Supplemental Figure 9	Tamoxifen	24
Supplemental Figure 10	Aromatase inhibitors	25
Supplemental Figure 11	Extended endocrine therapy	26
Supplemental Figure 12	Ovarian suppression/ablation	27
Supplemental Figure 13	Bisphosphonates	29
Supplemental Figure 14	Radiotherapy	30

Rate ratios		***Page***
Supplemental Table 3	Risk ratios and p-values for studies where rate ratios were not reported	34
Supplemental Table 4	Calendar periods when adjuvant and neoadjuvant early breast cancer treatments were first explicitly described in clinical guidelines	38
Supplemental Table 5	Rate ratios for the effects of adjuvant and neoadjuvant breast cancer treatments on breast cancer and non-breast-cancer mortality	42
Supplemental Table 6	Rate ratios for the effects of adjuvant and neoadjuvant breast cancer treatments not at present reported to reduce breast cancer mortality	47
Supplemental Table 7	Rate ratios for the effects of adjuvant and neoadjuvant breast cancer treatments on individual causes of non-breast-cancer mortality	49
Supplemental Table 8	Epidemiological studies in which dose-response relationships for radiation-related diseases have been derived	53

Supplemental Table 1. Chemotherapy treatment options recommended in guidelines

	NCCN[1]	ASCO[2,3]	ESMO[4]	St Gallen[5]	NICE[6]
**Regimen**					
Anthracycline-based					
Dose-dense AC	X				
AC	X	X		X	
EC	X				
Anthracycline+taxane					
Dose-dense AC - paclitaxel every 2 weeks	X	X			
Dose-dense AC - paclitaxel every week	X	X			
Dose-dense EC - paclitaxel every 2 weeks		X			
AC - paclitaxel every week	X	X			
AC - docetaxel every 3 weeks	X	X			
AC - docetaxel	X				
FEC - docetaxel		X			
Docetaxel AC (combination)	X	X			
Taxane-based					
Paclitaxel	X			X	
Carboplatin paclitaxel every week	X				
Docetaxel cyclophosphamide	X	X			
Docetaxel or paclitaxel carboplatin (neoadjuvant)	X	X			
**Category**					
Anthracycline-based			X	X	X
Anthracycline+taxane			X	X	X
Taxane-based			X	X	
Platinum-based			X		X
**Other regimens**					
CMF (selected patients)	X	X	X		
Capecitabine (HER2- residual disease after neoadjuvant chemotherapy)	X	X	X	X	

Abbreviations: NCCN National Comprehensive Cancer Network; ASCO American Society of Clinical Oncology; ESMO European Society of Medical Oncology; St Gallen St Gallen International Consensus Guidelines; NICE National Institute for Health and Care Excellence; A doxorubicin; C cyclophosphamide; F fluorouracil; E epirubicin; M methotrexate.
US guidelines listed chemotherapy categories and individual regimens whereas European guidelines usually listed just categories.

References for Supplemental Table 1

1 National Comprehensive Cancer Network Guidelines Version 2.2022 Breast Cancer. https://www.nccn.org/ [accessed 4th Feb 2022]

2 Denduluri N, Chavez-MacGregor M, Telli ML, Eisen A, Graff SL, Hassett MJ, et al. Selection of optimal adjuvant chemotherapy and targeted therapy for early breast cancer: ASCO clinical practice guideline focused update. J Clin Oncol 2018;36:2433-43. https://doi.org/10.1200/JCO.2018.78.8604

3 Korde LA, Somerfield MR, Carey LA, Crews JR, Denduluri N, Hwang ES, et al. Neoadjuvant chemotherapy, endocrine therapy, and targeted therapy for breast cancer: ASCO guideline. JCO 2021;39:1485-1505. https://ascopubs.org/doi/full/10.1200/JCO.20.03399

4 Cardoso F, Kyriakides S, Ohno S, Penault-Llorca F, Poortmans P, Rubio IT, et al. On behalf of the ESMO Guidelines Committee. Early breast cancer: ESMO Clinical Practice Guidelines for diagnosis, treatment and follow-up. Ann Oncol, 2019;30:1194–220. https://doi.org/10.1093/annonc/mdz173

5 Burstein HJ, Curigliano G, Thurlimann B, Weber WP, Poortmans P, Regan MM, et al. Customising local and systemic therapies for women with early breast cancer: the St. Gallen International Consensus Guidelines for treatment of early breast cancer 2021. Ann Oncol 2021;32:1216-35. https://doi.org/10.1016/j.annonc.2021.06.023

6 NICE (National Institute for Health and Care Excellence) guideline. Early and locally advanced breast cancer: diagnosis and management, 2018. https://www.nice.org.uk/guidance/ng101 [accessed 7th Nov 2020].

Supplemental Table 2a. Embase and Medline database searches for rate ratios of the effects of adjuvant or neoadjuvant breast cancer treatments

Category	Treatment	Search terms in the title or abstract or keywords†
Chemotherapy	Anthracycline-based	(breast cancer.mp. OR Breast Neoplasms/) AND (Meta analysis/ OR meta analysis.mp. OR Meta-analysis/ OR meta-analysis.mp) AND (Anthracyclines/ OR anthracycline*.mp. OR doxorubicin.mp. OR Doxorubicin/ OR Epirubicin/ OR epirubicin.mp.)
	Taxane-based	(breast cancer.mp. OR Breast Neoplasms/) AND (Meta analysis/ OR meta analysis.mp. OR Meta-analysis/ OR meta-analysis.mp) AND (Taxoids/ OR Taxanes/ OR taxane*.mp. OR Paclitaxel/ OR paclitaxel.mp. OR Docetaxel/ OR docetaxel.mp.)
	Platinum	(breast cancer.mp. OR Breast Neoplasms/) AND (Meta analysis/ OR meta analysis.mp. OR Meta-analysis/ OR meta-analysis.mp) AND (Platinum/ OR platinum.mp)
	Capecitabine	(breast cancer.mp. OR Breast Neoplasms/) AND (Meta analysis/ OR meta analysis.mp. OR Meta-analysis/ OR meta-analysis.mp) AND (capecitabine.mp. OR Capecitabine/)
		(breast cancer.mp. OR Breast Neoplasms/) AND (randomized controlled trial.mp. OR Randomized Controlled Trial/ OR controlled clinical trial.mp. OR Controlled Clinical Trial/ OR random*.mp. OR placebo.mp. OR clinical trial*.mp. OR Clinical Trial/ OR Double-Blind Method/ OR double blind.mp. OR Single-Blind Method/ OR single blind.mp.) AND (capecitabine.mp. OR Capecitabine/)
Anti HER2 therapy	Trastuzumab or trastuzumab emtansine	(breast cancer.mp. OR Breast Neoplasms/) AND (Meta analysis/ OR meta analysis.mp. OR Meta-analysis/ OR meta-analysis.mp) AND (trastuzumab emtansine.mp. OR Ado-Trastuzumab Emtansine/ OR trastuzumab.mp. OR Trastuzumab/ OR Herceptin.mp.)
		(breast cancer.mp. OR Breast Neoplasms/) AND (randomized controlled trial.mp. OR Randomized Controlled Trial/ OR controlled clinical trial.mp. OR Controlled Clinical Trial/ OR random*.mp. OR placebo.mp. OR clinical trial*.mp. OR Clinical Trial/ OR Double-Blind Method/ OR double blind.mp. OR Single-Blind Method/ OR single blind.mp.) AND (trastuzumab emtansine.mp. OR Ado-Trastuzumab Emtansine/ OR)
	Pertuzumab	(breast cancer.mp. OR Breast Neoplasms/) AND (Meta analysis/ OR meta analysis.mp. OR Meta-analysis/ OR meta-analysis.mp) AND (pertuzumab.mp.)

†Controlled vocabulary terms are indicated by a capital letter at the start and forward slash at the end of the term. Eg Meta-analysis/

Continued on next page

Category	Treatment	Search terms in the title or abstract or keywords[†]
Anti HER2 therapy (continued)	Pertuzumab (continued)	(breast cancer.mp. OR Breast Neoplasms/) AND (randomized controlled trial.mp. OR Randomized Controlled Trial/ OR controlled clinical trial.mp. OR Controlled Clinical Trial/ OR random*.mp. OR placebo.mp. OR clinical trial*.mp. OR Clinical Trial/ OR Double-Blind Method/ OR double blind.mp. OR Single-Blind Method/ OR single blind.mp.) AND (pertuzumab.mp.)
	Neratinib	(breast cancer.mp. OR Breast Neoplasms/) AND (Meta analysis/ OR meta analysis.mp. OR Meta-analysis/ OR meta-analysis.mp) AND (neratinib.mp. OR Protein Kinase Inhibitors/)
		(breast cancer.mp. OR Breast Neoplasms/) AND (randomized controlled trial.mp. OR Randomized Controlled Trial/ OR controlled clinical trial.mp. OR Controlled Clinical Trial/ OR random*.mp. OR placebo.mp. OR clinical trial*.mp. OR Clinical Trial/ OR Double-Blind Method/ OR double blind.mp. OR Single-Blind Method/ OR single blind.mp.) AND (neratinib.mp. OR Protein Kinase Inhibitors/)
Neoadjuvant timing of systemic therapy	Neoadjuvant	(breast cancer.mp. OR Breast Neoplasms/) AND (Meta analysis/ OR meta analysis.mp. OR Meta-analysis/ OR meta-analysis.mp) AND (Neoadjuvant Therapy/ OR neoadjuvant.mp)
Endocrine therapy	Tamoxifen	(breast cancer.mp. OR Breast Neoplasms/) AND (Meta analysis/ OR meta analysis.mp. OR Meta-analysis/ OR meta-analysis.mp) AND (tamoxifen.mp. OR Tamoxifen/)
	Aromatase inhibitors	(breast cancer.mp. OR Breast Neoplasms/) AND (Meta analysis/ OR meta analysis.mp. OR Meta-analysis/ OR meta-analysis.mp) AND (Aromatase Inhibitors/ OR aromatase inhibitor*.mp. OR anastrozole.mp. OR letrozole.mp. OR exemestane.mp.)
	Extended endocrine therapy	(breast cancer.mp. OR Breast Neoplasms/) AND (Meta analysis/ OR meta analysis.mp. OR Meta-analysis/ OR meta-analysis.mp) AND (tamoxifen.mp. OR Tamoxifen/ OR Aromatase Inhibitors/ OR aromatase inhibitor*.mp. OR anastrozole.mp. OR letrozole.mp. OR exemestane.mp.) AND (extended.mp)
		(breast cancer.mp OR Breast Neoplasm/) AND (tamoxifen.mp. OR Tamoxifen/ OR Aromatase Inhibitors/ OR aromatase inhibitor*.mp. OR anastrozole.mp. OR letrozole.mp. OR exemestane.mp.) AND (extended.mp) AND (randomized controlled trial.mp. OR Randomized Controlled Trial/ OR controlled clinical trial.mp. OR Controlled Clinical Trial/ OR random*.mp. OR placebo.mp. OR clinical trial*.mp. OR Clinical Trial/ OR Double-Blind Method/ OR double blind.mp. OR Single-Blind Method/ OR single blind.mp.)

[†]Controlled vocabulary terms are indicated by a capital letter at the start and forward slash at the end of the term. Eg Meta-analysis/

Continued on next page

Category	Treatment	Search terms in the title or abstract or keywords†
Endocrine therapy (continued)	Ovarian suppression/ Ablation	(breast cancer.mp. OR Breast Neoplasms/) AND (Meta analysis/ OR meta analysis.mp. OR Meta-analysis/ OR meta-analysis.mp) AND (ovarian suppression.mp. OR ovarian ablation.mp. OR oophorectomy.mp. OR Ovariectomy/ OR salpingo-oophorectomy.mp. OR Salpingo-oophorectomy/ OR TAHBSO.mp. OR ovariectomy.mp. OR ovarian irradiation.mp. OR radiation menopause.mp. OR radiation-induced menopause.mp. OR LHRH analogues.mp. OR goserelin.mp. OR Goserelin/ OR Zoladex.mp. OR gonadotropin releasing hormone agonist.mp. OR Luteinizing hormone-releasing hormone.mp. OR Gonadotropin-Releasing Hormone/ OR GnRH agonists.mp. OR leuprolide acetate.mp. OR Leuprolide/ OR Leuprolide.mp. OR Triptorelin.mp. OR Triptorelin Pamoate/ OR buserelin.mp. OR Buserelin/)
		(breast cancer.mp. OR Breast Neoplasms/) AND (randomized controlled trial.mp. or Randomized Controlled Trial/ OR controlled clinical trial.mp. or Controlled Clinical Trial/ OR random*.mp. OR placebo.mp. OR clinical trial*.mp. or Clinical Trial/ OR Double-Blind Method/ or double blind.mp. or Single-Blind Method/ or single blind.mp.) AND (ovarian suppression.mp. OR ovarian ablation.mp. OR oophorectomy.mp. OR Ovariectomy/ OR salpingo-oophorectomy.mp. OR Salpingo-oophorectomy/ OR TAHBSO.mp. OR ovariectomy.mp. OR ovarian irradiation.mp. OR radiation menopause.mp. OR radiation-induced menopause.mp. OR LHRH analogues.mp. OR goserelin.mp. OR Goserelin/ OR Zoladex.mp. OR gonadotropin releasing hormone agonist.mp. OR Luteinizing hormone-releasing hormone.mp. OR Gonadotropin-Releasing Hormone/ OR GnRH agonists.mp. OR leuprolide acetate.mp. OR Leuprolide/ OR Leuprolide.mp. OR Triptorelin.mp. or Triptorelin Pamoate/ OR buserelin.mp. OR Buserelin/)
Bisphosphonates	Any type	(breast cancer.mp. OR Breast Neoplasms/) AND (Meta analysis/ OR meta analysis.mp. OR Meta-analysis/ OR meta-analysis.mp) AND (bisphosphonate.mp. or Diphosphonates/ or bisphosphonate*.mp. or Diphosphonates/ or clodronate.mp. or Clodronic Acid/ or zoledronate.mp. or Zoledronate/ OR zoledronic acid.mp. or pamidronate.mp. or risedronate.mp. or Risedronate Sodium/ or ibandronate.mp. or alendronate.mp. or Alendronate/)
Radiotherapy	Any surgery, target or nodes	(breast cancer.mp. OR Breast Neoplasms/) AND (Meta analysis/ OR meta analysis.mp. OR Meta-analysis/ OR meta-analysis.mp) AND (Radiotherapy/ OR radiother* OR radiat* OR irradiat*)
	Tumour bed boost	(breast cancer.mp OR Breast Neoplasm/) AND (radiotherapy.mp or Radiotherapy/) AND (boost.mp) AND (randomized controlled trial.mp. or Randomized Controlled Trial/ OR controlled clinical trial.mp. or Controlled Clinical Trial/ OR random*.mp. OR placebo.mp. OR clinical trial*.mp. or Clinical Trial/ OR Double-Blind Method/ or double blind.mp. or Single-Blind Method/ or single blind.mp.)

†Controlled vocabulary terms are indicated by a capital letter at the start and forward slash at the end of the term. Eg Meta-analysis/

Continued on next page

Category	Treatment	Search terms in the title or abstract or keywords[†]
Radiotherapy (continued)	Partial breast radiotherapy	(breast cancer.mp OR Breast Neoplasm/) AND (radiotherapy.mp or Radiotherapy/) AND (partial.mp) AND (randomized controlled trial.mp. or Randomized Controlled Trial/ OR controlled clinical trial.mp. or Controlled Clinical Trial/ OR random*.mp. OR placebo.mp. OR clinical trial*.mp. or Clinical Trial/ OR Double-Blind Method/ or double blind.mp. or Single-Blind Method/ or single blind.mp.)
	Regional node radiotherapy	(breast cancer.mp OR Breast Neoplasm/) AND (radiotherapy.mp or Radiotherapy/) AND (lymph node.mp) AND (randomized controlled trial.mp. or Randomized Controlled Trial/ OR controlled clinical trial.mp. or Controlled Clinical Trial/ OR random*.mp. OR placebo.mp. OR clinical trial*.mp. or Clinical Trial/ OR Double-Blind Method/ or double blind.mp. or Single-Blind Method/ or single blind.mp.)

[†]Controlled vocabulary terms are indicated by a capital letter at the start and forward slash at the end of the term. Eg Meta-analysis/

Supplemental Table 2b. Database searches for radiation dose-response relationships and doses from typical modern breast cancer radiotherapy for mortality risks significantly increased by radiotherapy

Category	Search terms in the title or abstract or keywords
**Radiotherapy dose-response relationships**	(((per gray.mp) OR per Gy.mp) AND breast cancer.mp OR Breast Neoplasms) AND radiation/radiotherapy*))
**Organ doses from modern RT**	dos* AND breast* AND cancer*/carcinoma*/tumor*/tumour* AND radiation/radiotherapy*

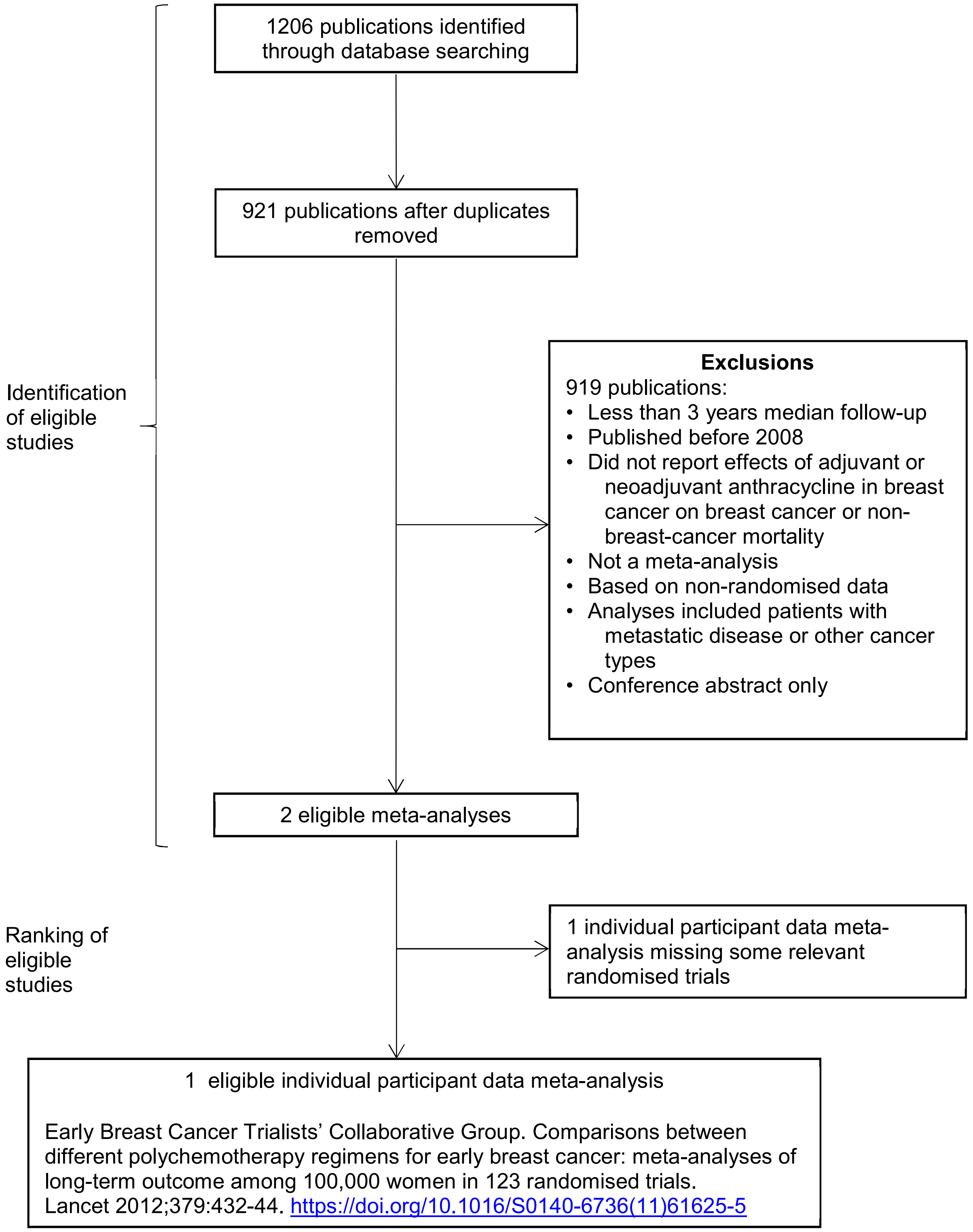

Supplemental Figure 1. Anthracycline chemotherapy in adjuvant or neoadjuvant breast cancer treatment: the process of study identification of *meta-analyses*

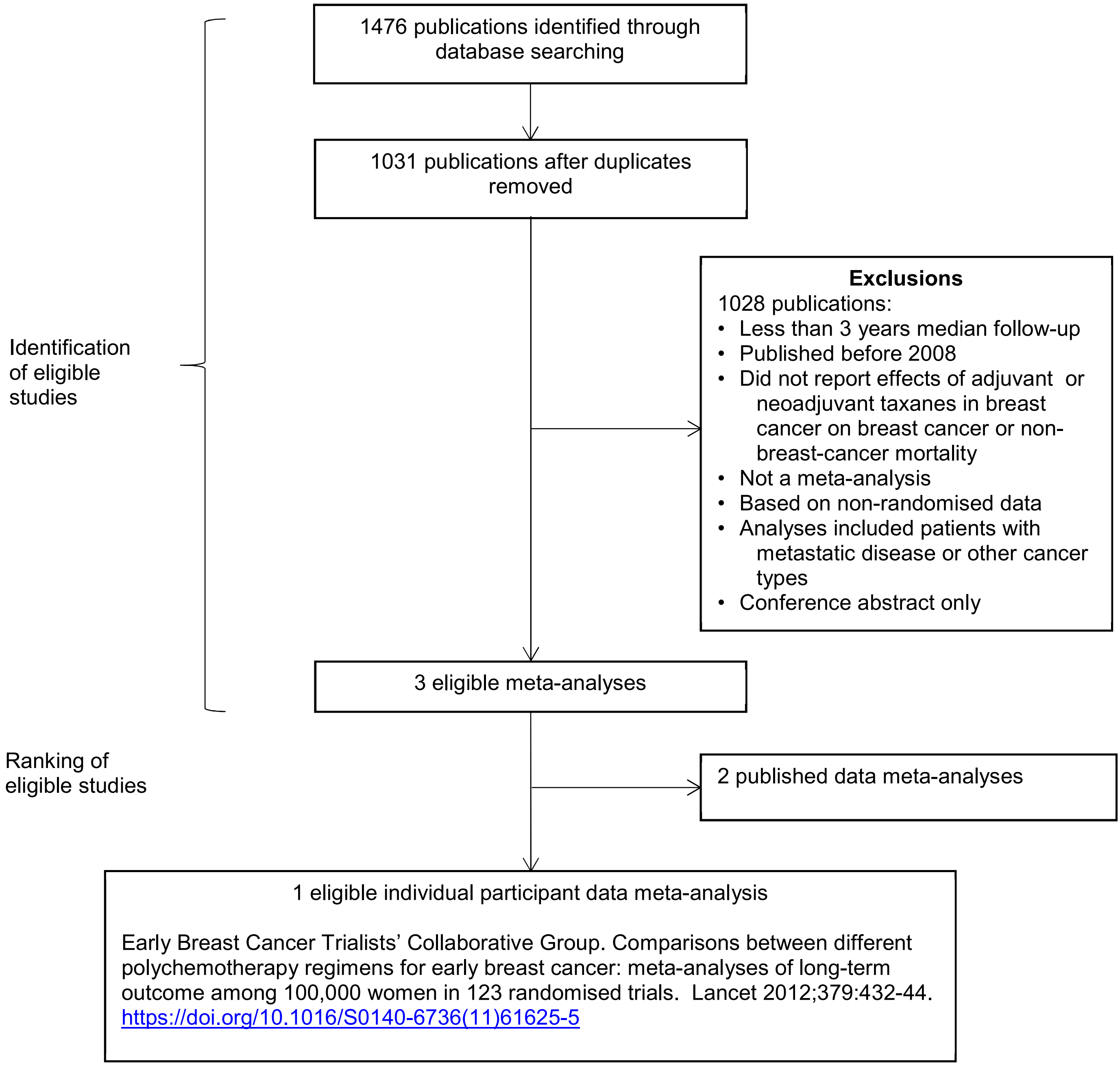

Supplemental Figure 2. Taxane + anthracycline chemotherapy in adjuvant or neoadjuvant breast cancer treatment: the process of study identification of *meta-analyses*

Continued from previous page

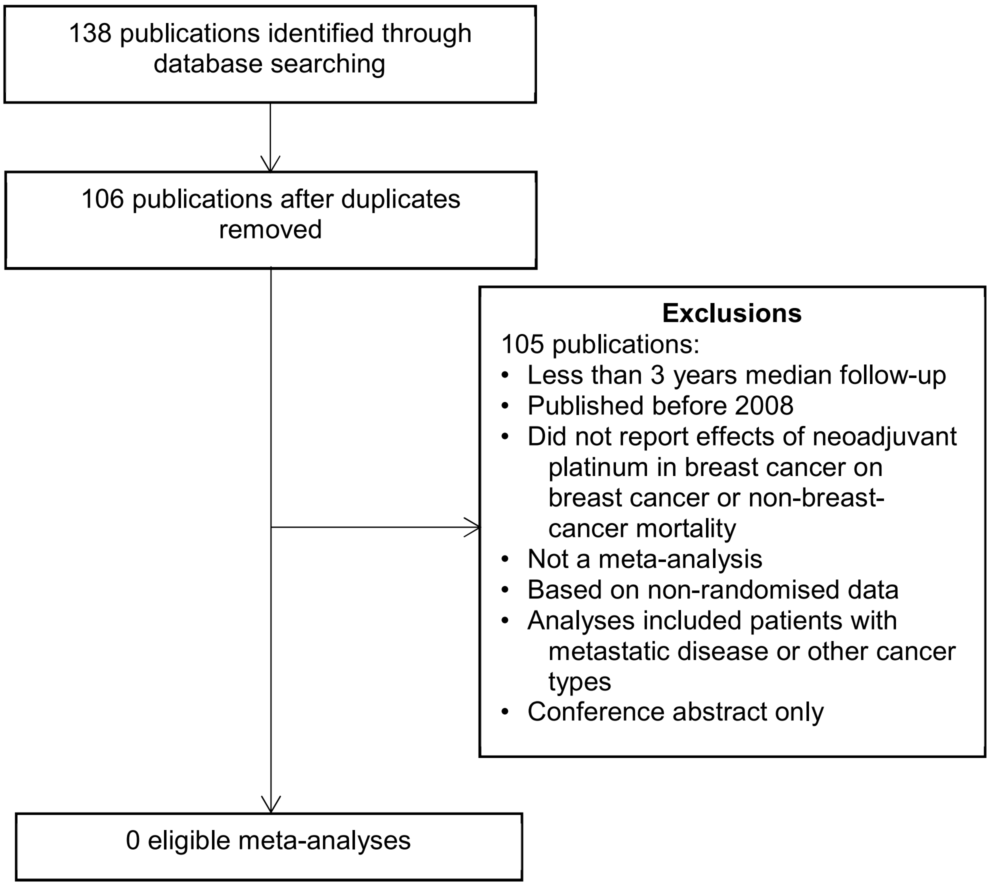

1 published data meta-analysis reporting pathological complete response*

Poggio F, Bruzzone M, Ceppi M, Ponde NF, La Valle G, Del Mastro L, et al. Platinum-based neoadjuvant chemotherapy in triple-negative breast cancer: a systematic review and meta-analysis. Ann Oncol 2018;29:14971508. https://doi.org/10.1093/annonc/mdy127

Supplemental Figure 3a. Platinum chemotherapy in neoadjuvant breast cancer treatment: the process of study identification of *meta-analyses*

*Guidelines recommend platinum based on the outcome complete pathological response. Therefore we listed the study that included the largest number of women with information on complete pathological response

Continued on next page

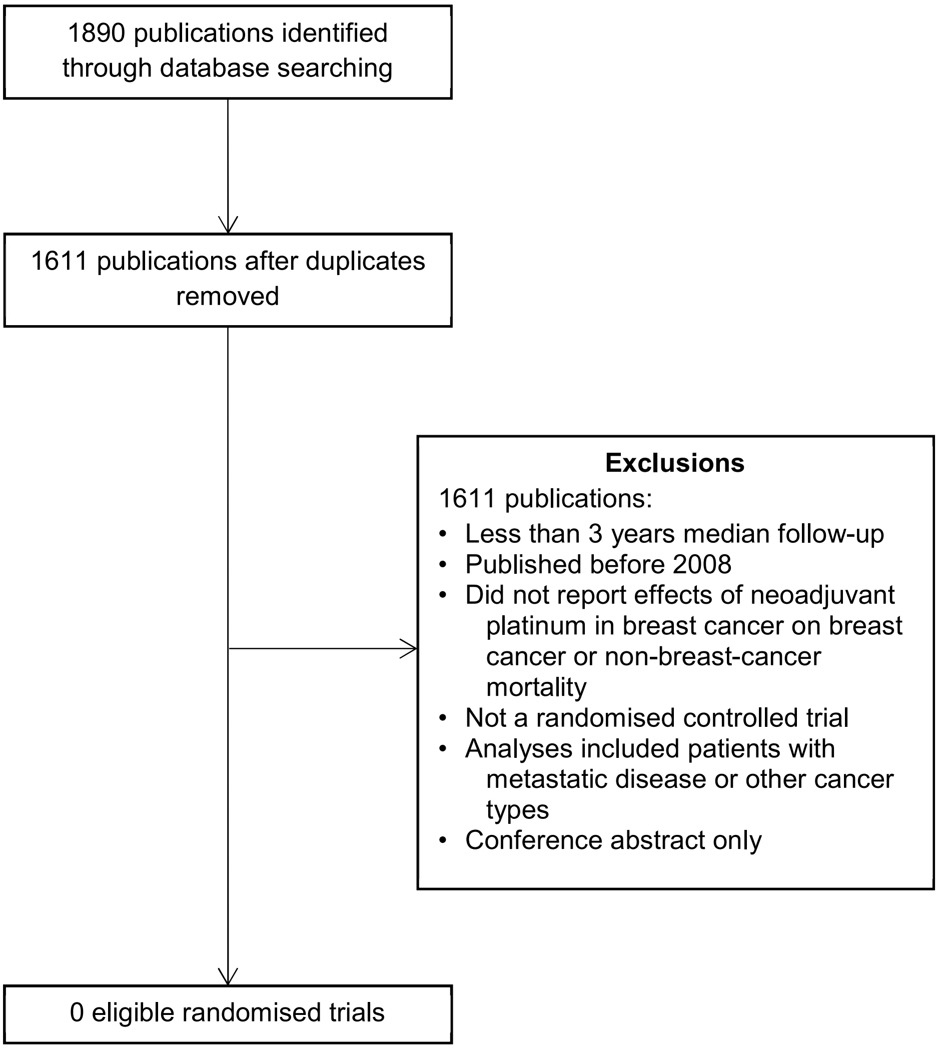

Supplemental Figure 3b. Platinum chemotherapy in neoadjuvant breast cancer treatment: the process of study identification of *randomised trials*

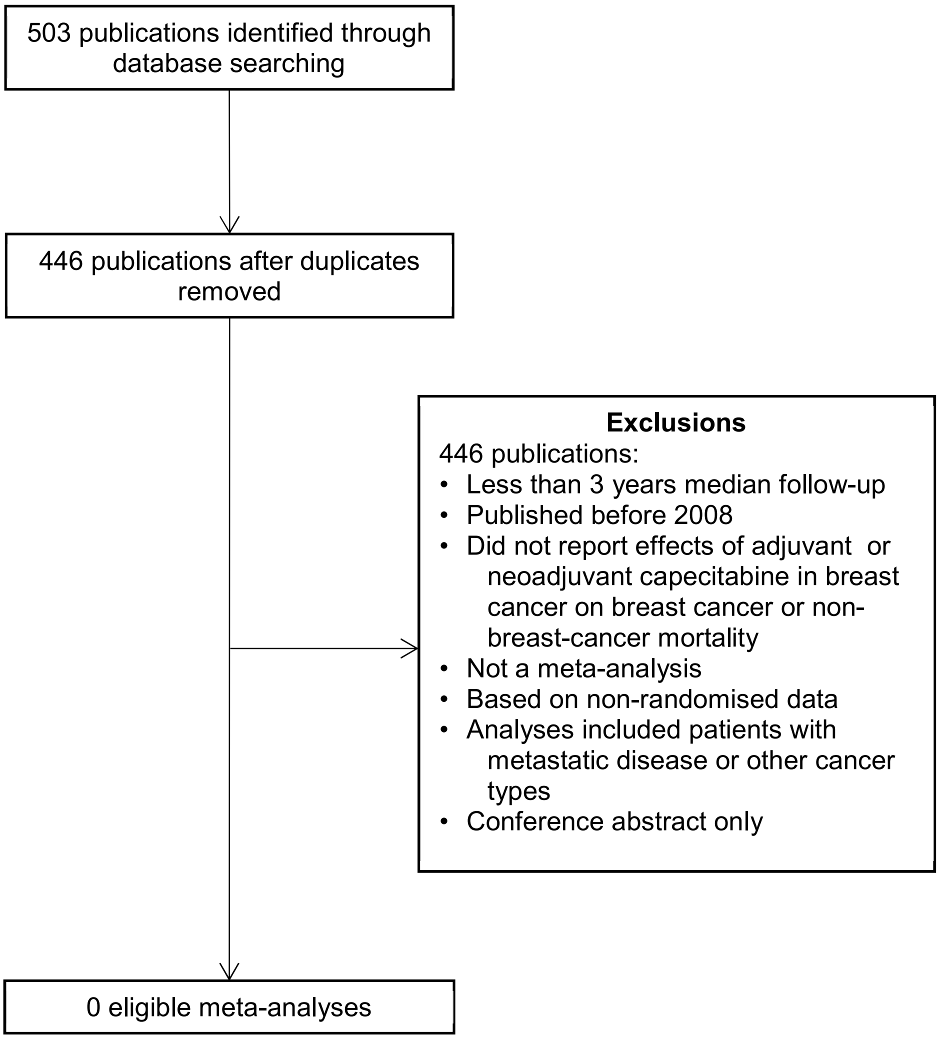

Supplemental Figure 4a. Capecitabine chemotherapy in adjuvant or neoadjuvant breast cancer treatment: the process of study identification of *meta-analyses*

Continued on next page

Continued from previous page

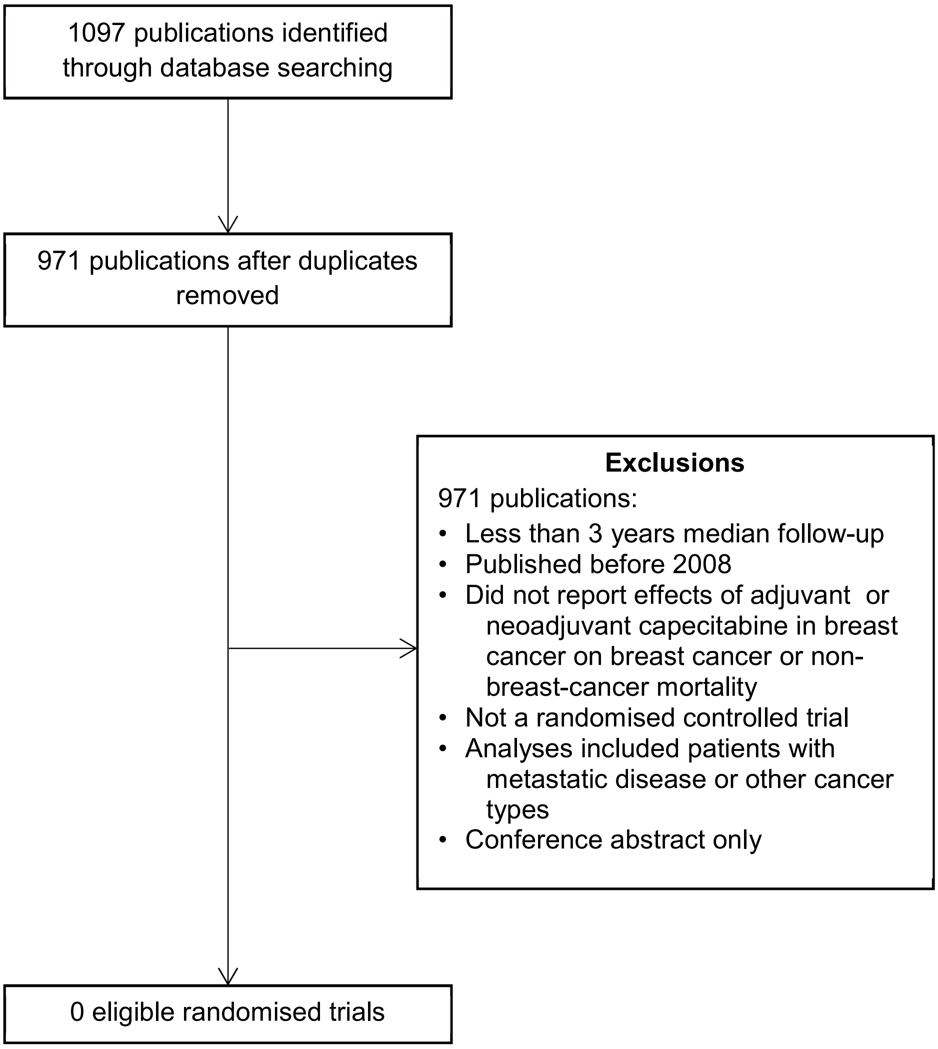

The following randomised trial was referenced in guidelines:

Masuda N, Lee SJ, Ohtani S, Im YH, Lee ES, Yokota I, et al. Adjuvant capecitabine for breast cancer after preoperative chemotherapy. N Eng J Med; 2017;376:2147-59. https://doi.org/10.1056/NEJMoa1612645

Supplemental Figure 4b. Capecitabine chemotherapy in adjuvant or neoadjuvant breast cancer treatment: the process of study identification of *randomised trials*

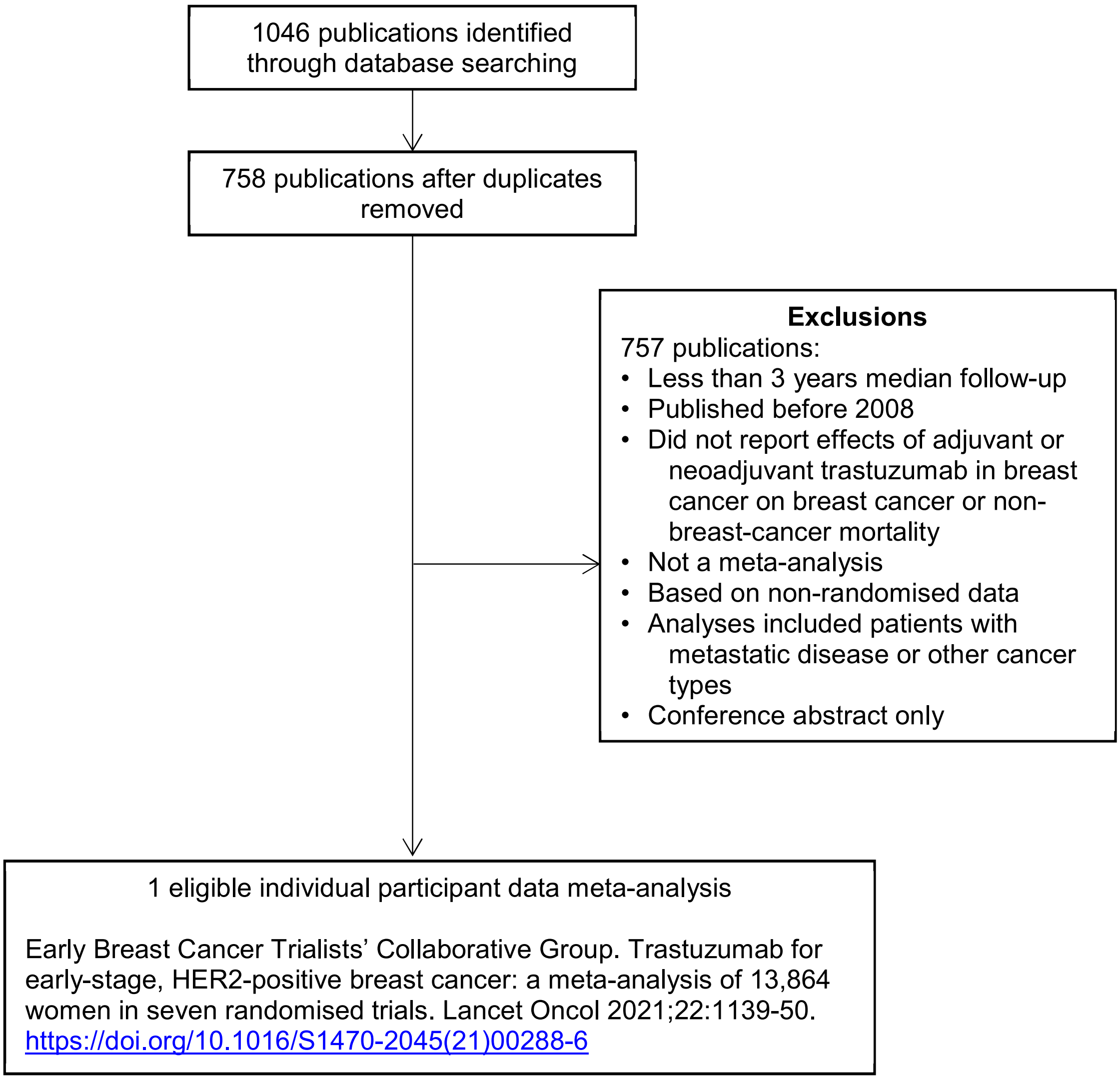

Supplemental Figure 5a. Trastuzumab in adjuvant or neoadjuvant breast cancer treatment: the process of study identification of *meta-analyses*

Continued on next page

Continued from previous page

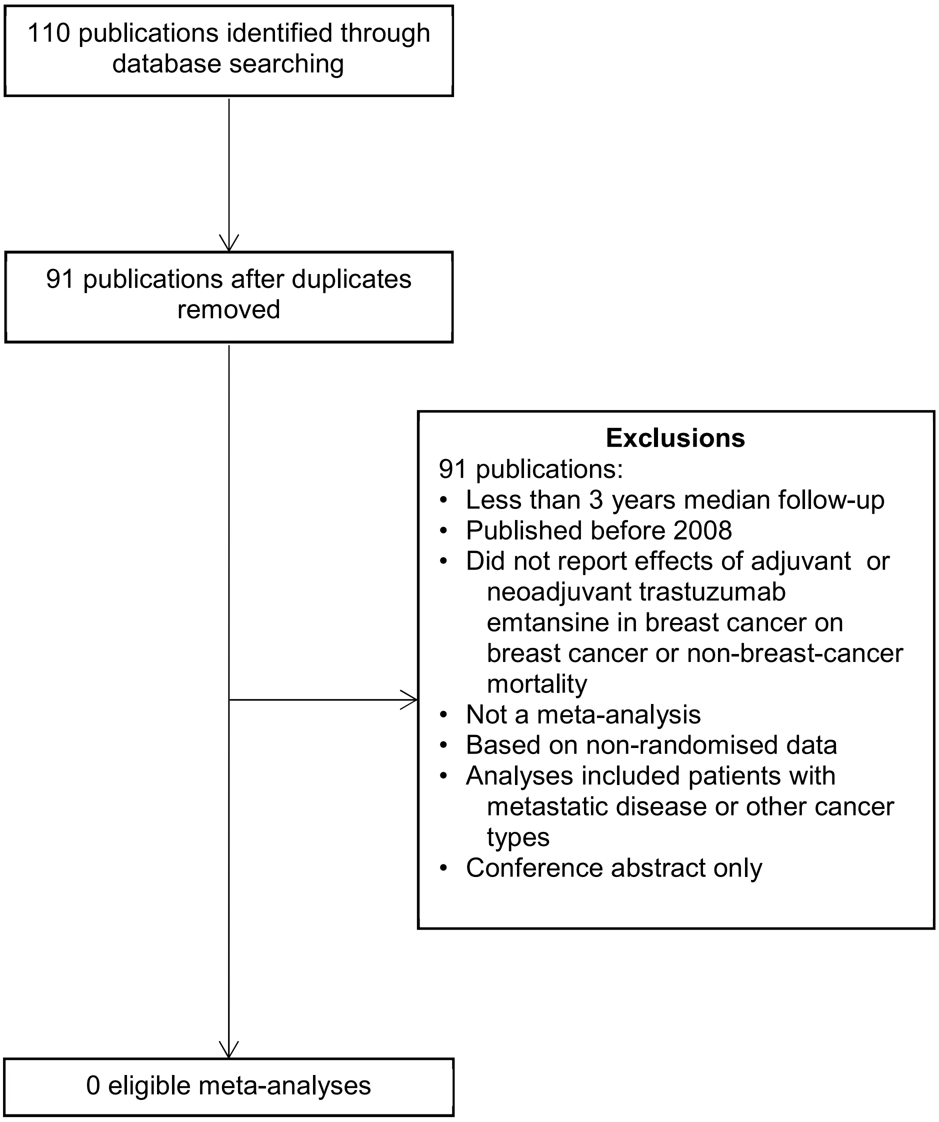

Supplemental Figure 5b. Trastuzumab emtansine in adjuvant or neoadjuvant breast cancer treatment: the process of study identification of *meta-analyses*

Continued on next page

Continued from previous page

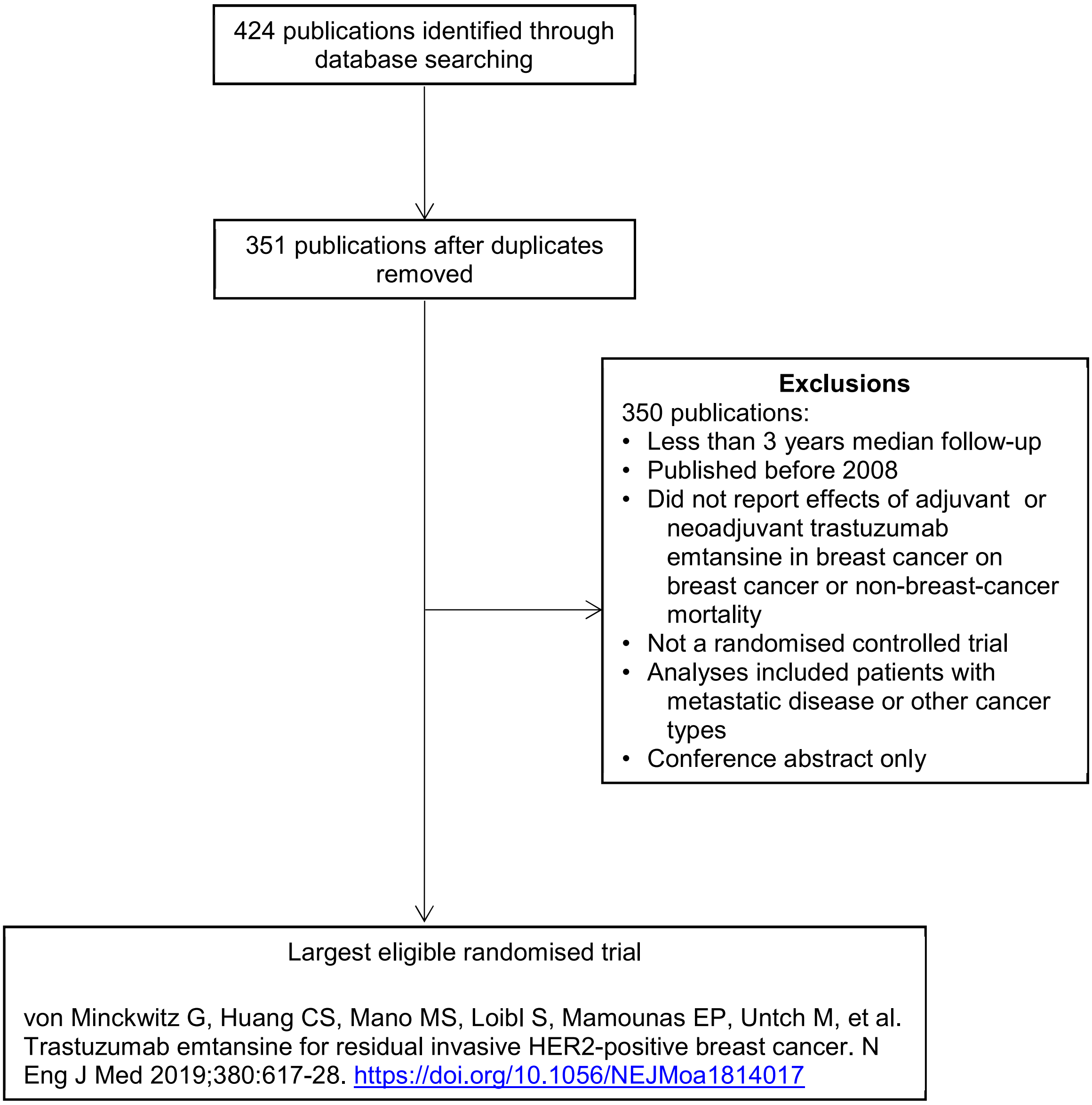

Supplemental Figure 5c. Trastuzumab emtansine in adjuvant or neoadjuvant breast cancer treatment: the process of study identification of *randomised trials*

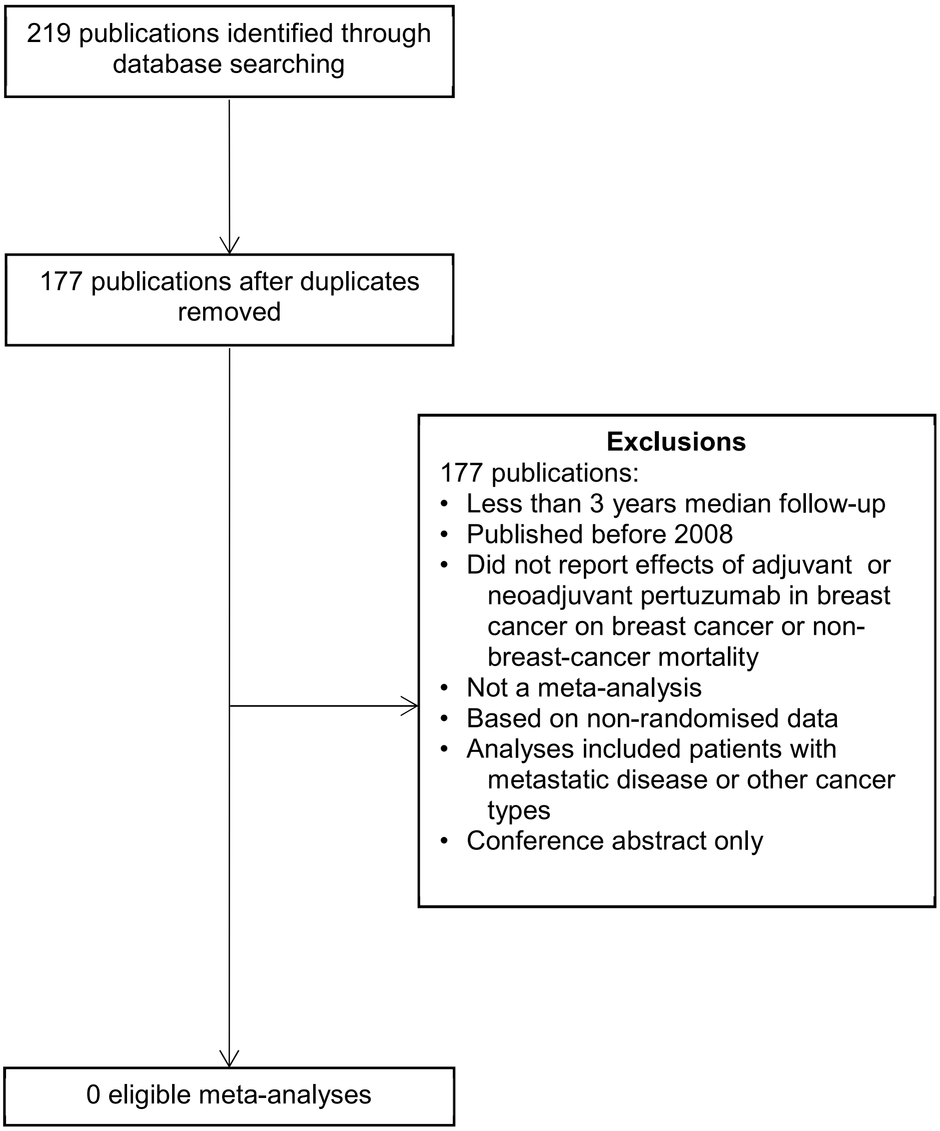

Supplemental Figure 6a. Pertuzumab in adjuvant or neoadjuvant breast cancer treatment: the process of study identification of *meta-analyses*

Continued on next page

Continued from previous page

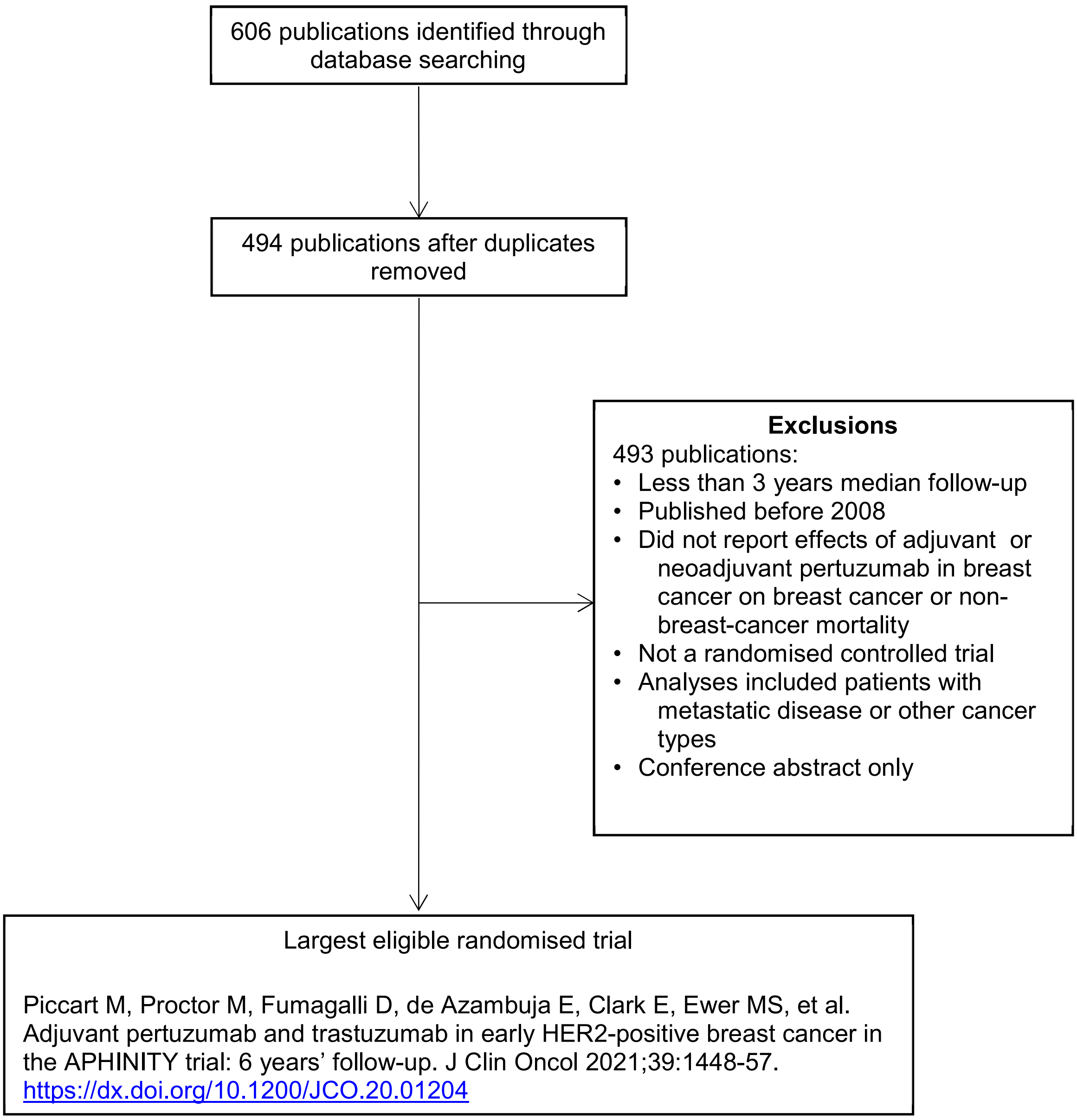

Supplemental Figure 6b. Pertuzumab in adjuvant or neoadjuvant breast cancer treatment: the process of study identification of *randomised trials*

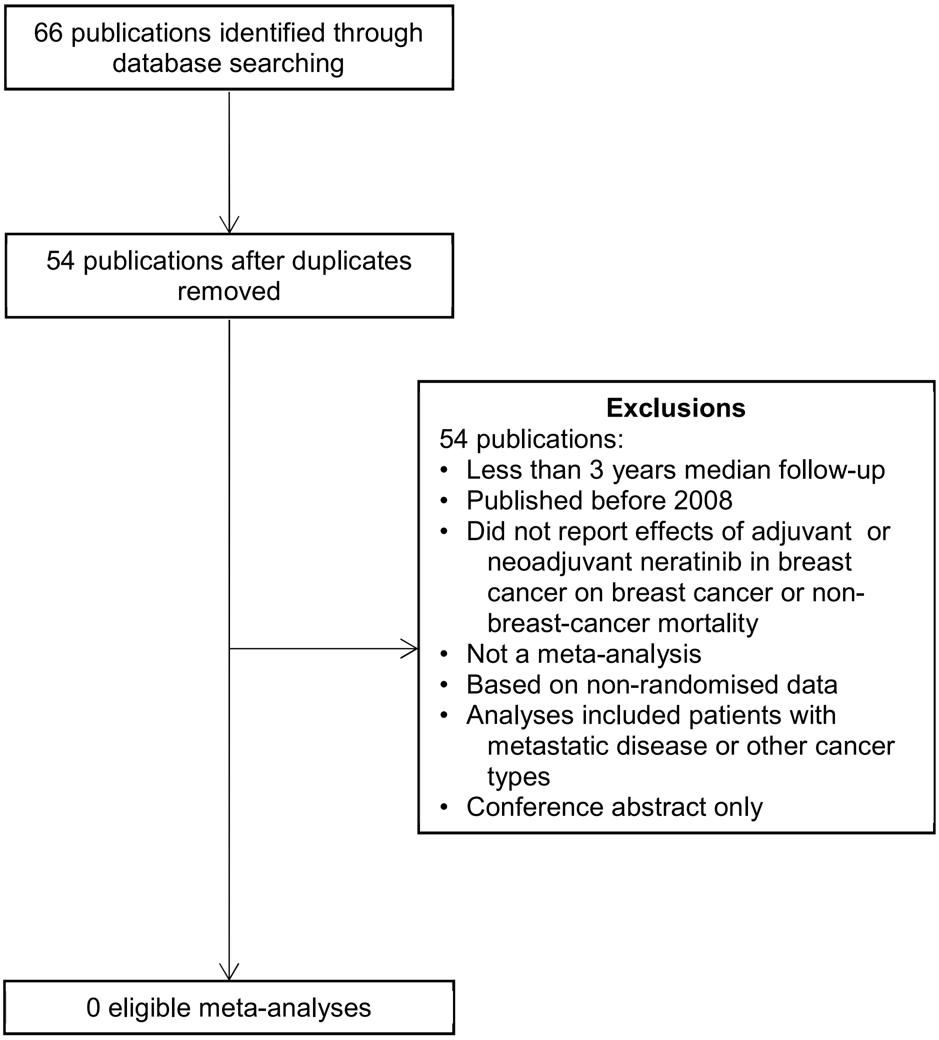

Supplemental Figure 7a. Neratinib in adjuvant or neoadjuvant breast cancer treatment: the process of study identification of *meta-analyses*

Continued on next page

Continued from previous page

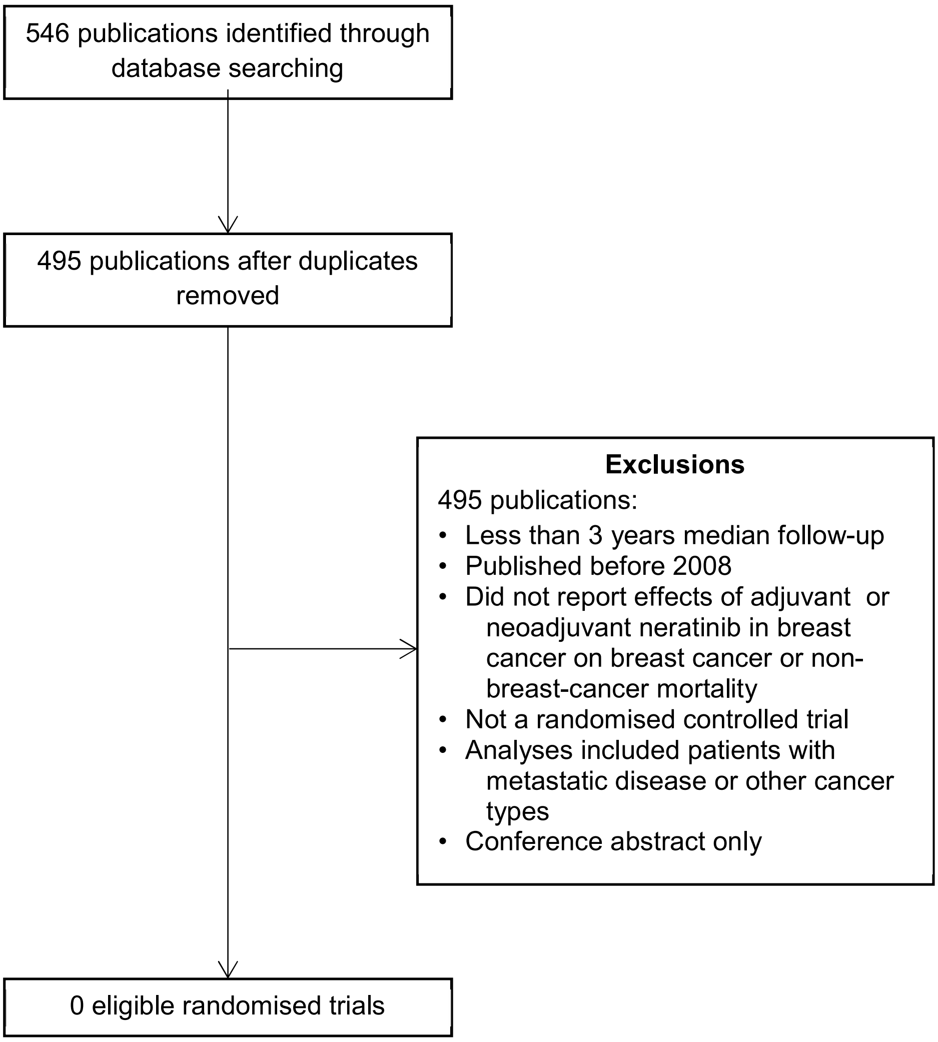

The following randomised trial was referenced in guidelines:

Martin M, Holmes FA, Ejlertsen B, Delaloge S, May B, Iwata H, et al. Neratinib after trastuzumab-based adjuvant therapy in HER2-positive breast cancer (ExteNET): 5-year analysis of a randomised, double-blind, placebo-controlled, phase 3 trial. Lancet Oncol 2017;18:1688-700. https://doi.org/10.1016/S1470-2045(17)30717-9

Supplemental Figure 7b. Neratinib in adjuvant or neoadjuvant breast cancer treatment: the process of study identification of *randomised trials*

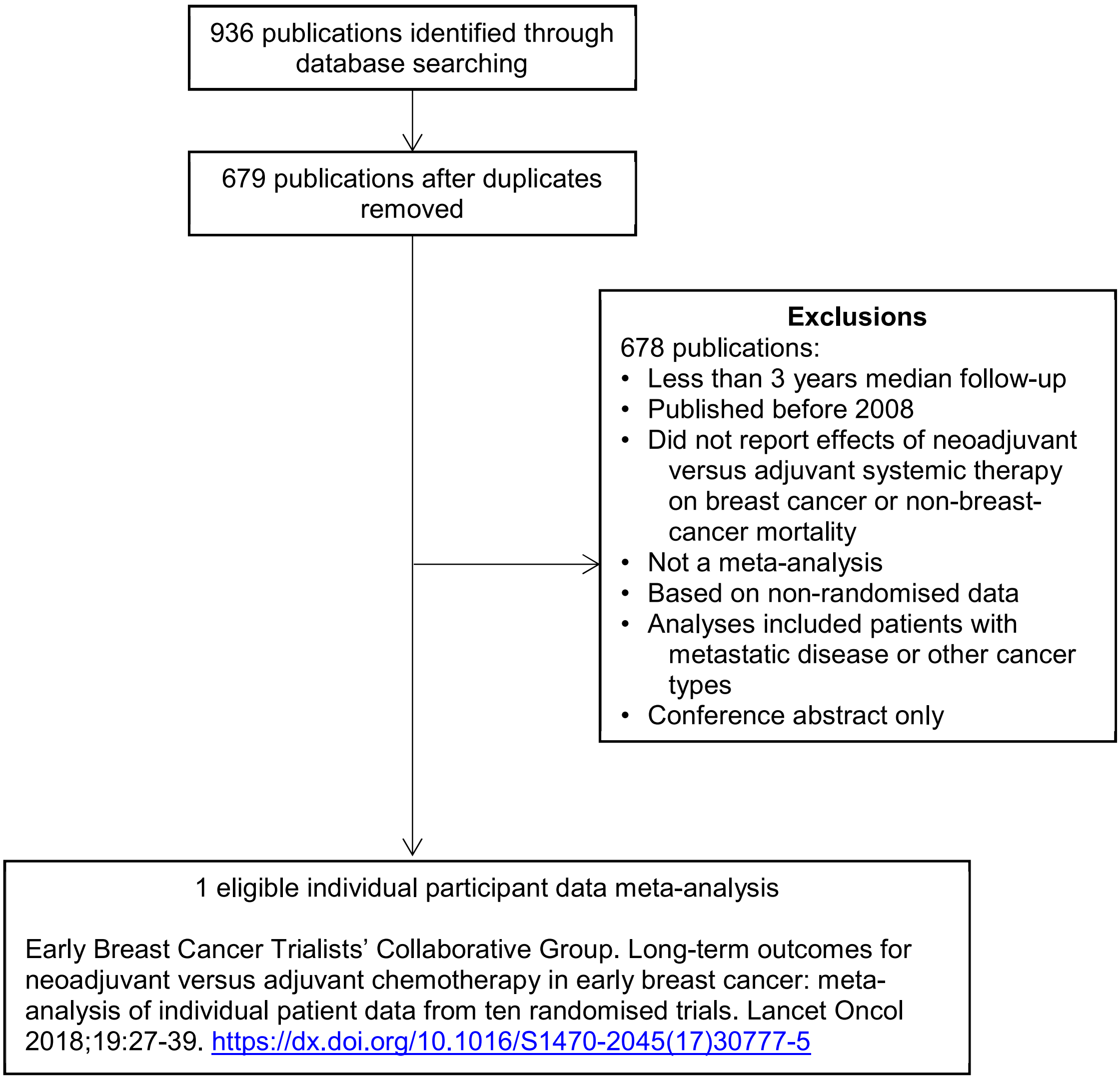

Supplemental Figure 8. Systemic therapy given neoadjuvantly versus adjuvantly in breast cancer treatment: the process of study identification of *meta-analyses*

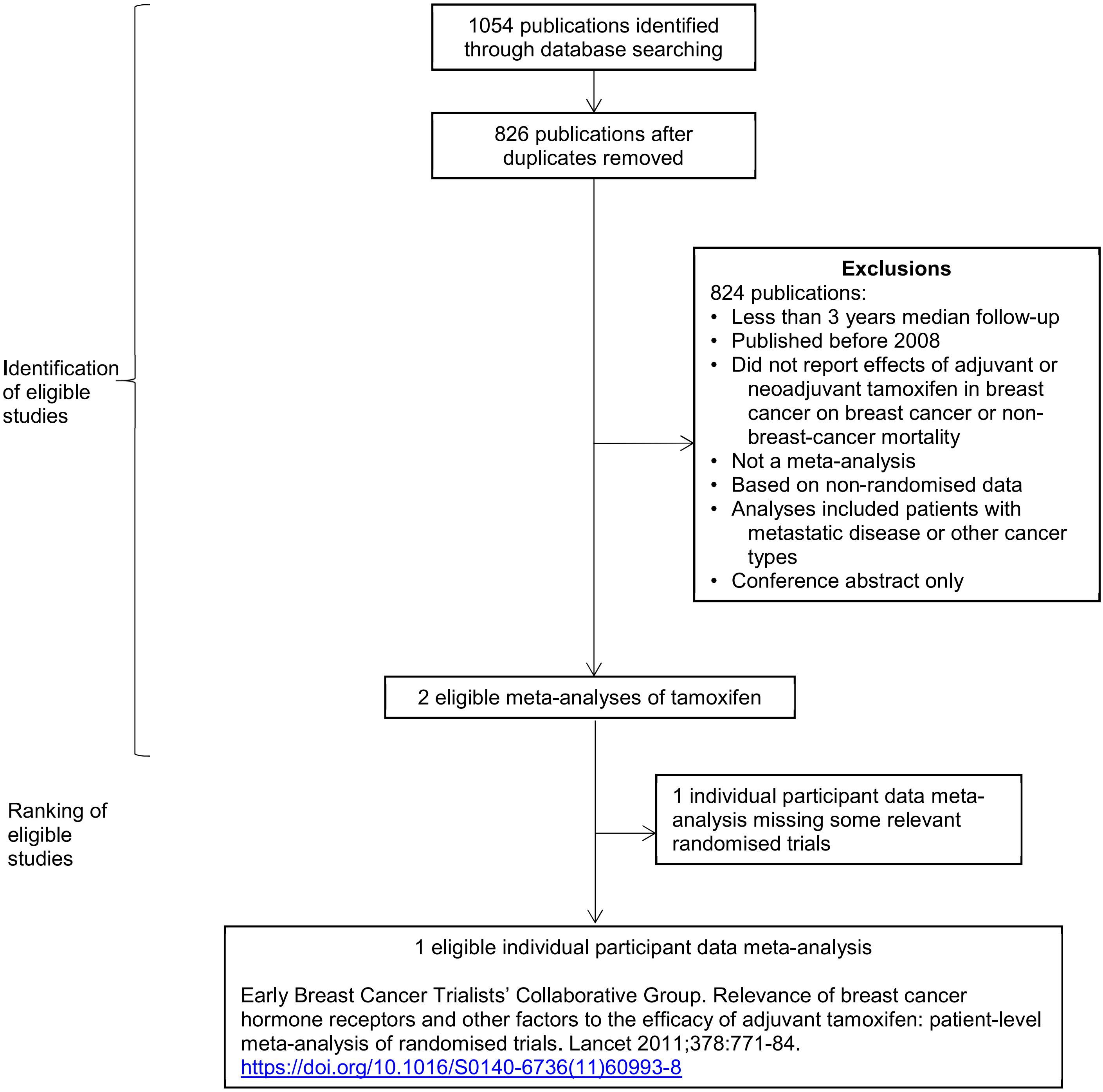

Supplemental Figure 9. Tamoxifen in adjuvant or neoadjuvant breast cancer treatment: the process of study identification of *meta-analyses*

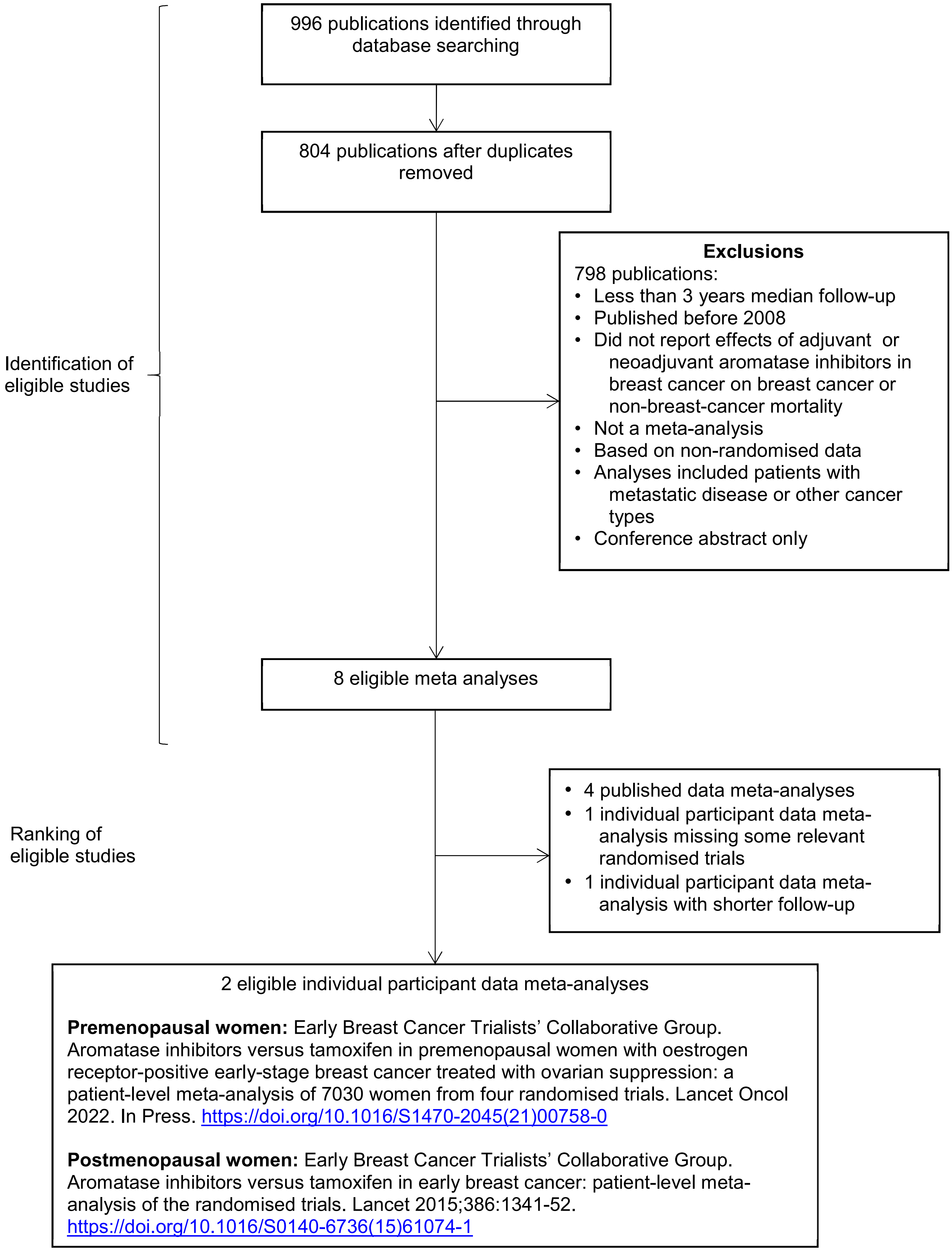

Supplemental Figure 10. Aromatase inhibitors in adjuvant or neoadjuvant breast cancer treatment: the process of study identification of *meta-analyses*

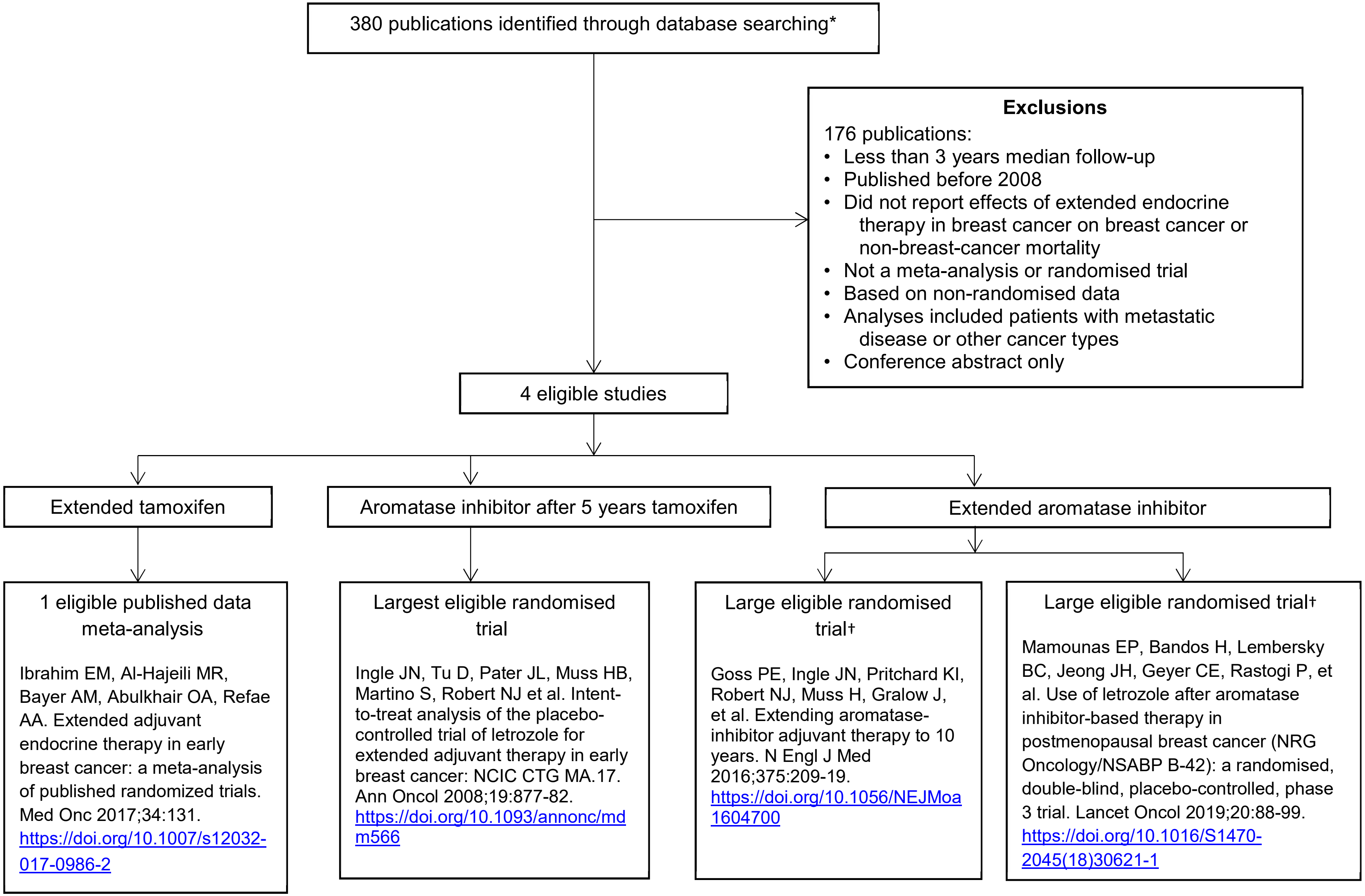

Supplemental Figure 11. Extended endocrine therapy in breast cancer treatment: the process of study identification of *meta-analyses and randomised trials*

*There were no duplicate records in this search

†Two randomised trials with differing designs were included because guidelines differed in their recommendations as to optimal AI duration

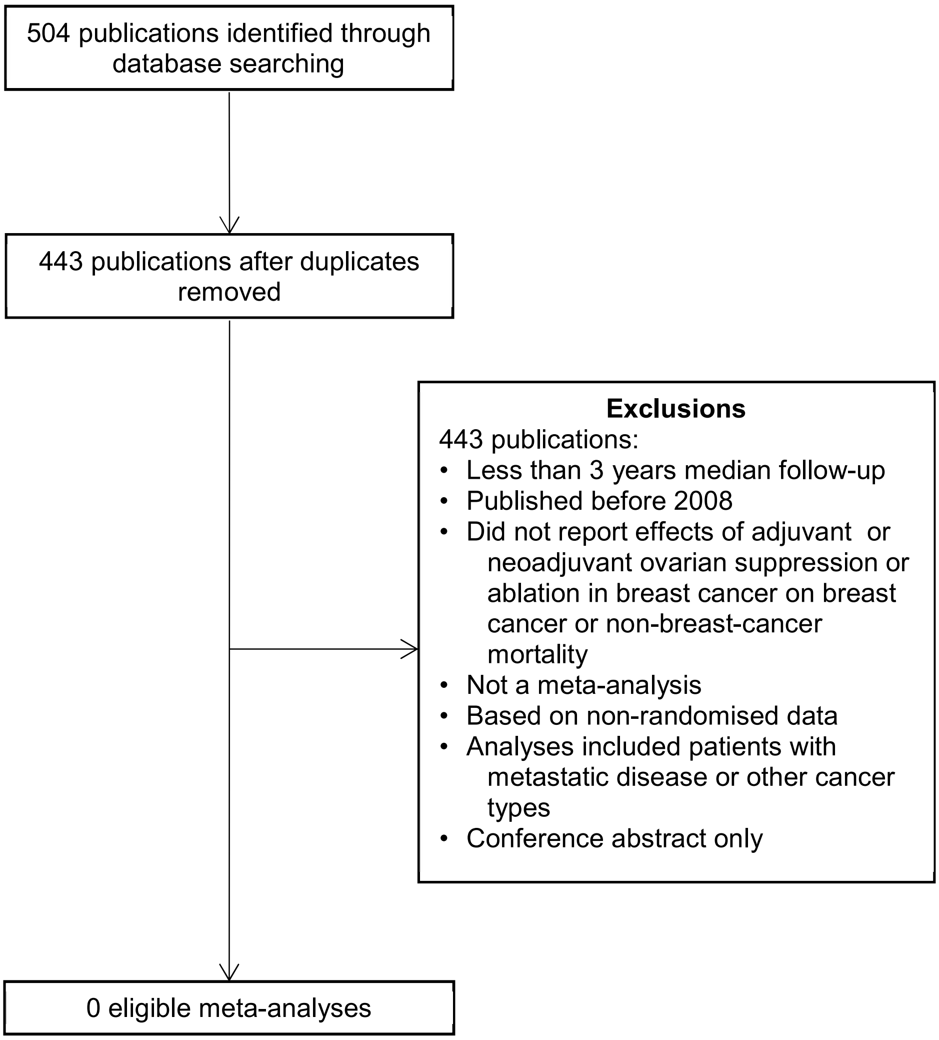

Supplemental Figure 12a. Ovarian suppression/ablation in adjuvant or neoadjuvant breast cancer treatment: the process of study identification of *meta-analyses*

Continued on next page

Continued from previous page

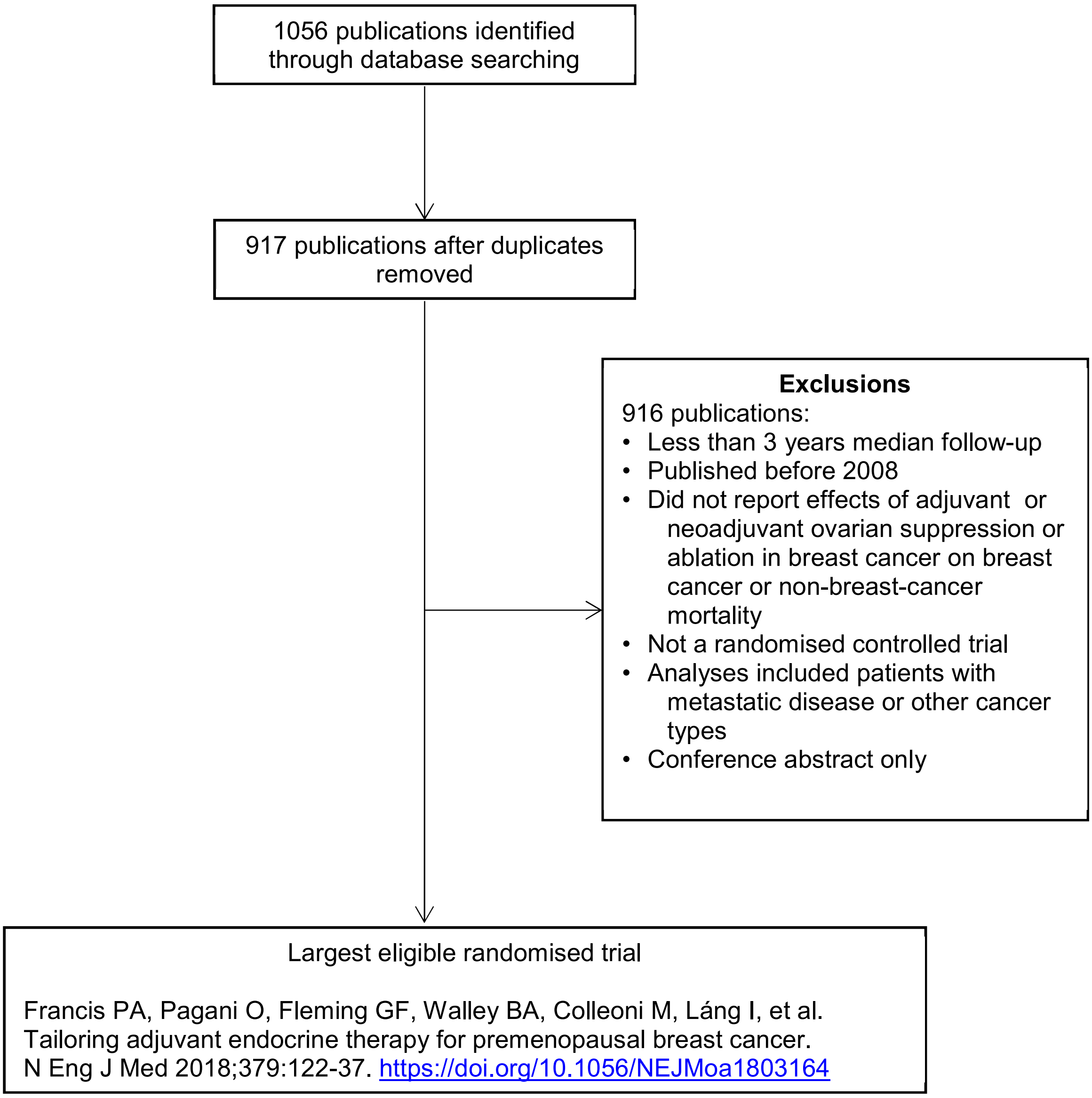

Supplemental Figure 12b. Ovarian suppression/ablation in adjuvant or neoadjuvant breast cancer treatment: the process of study identification of *randomised trials*

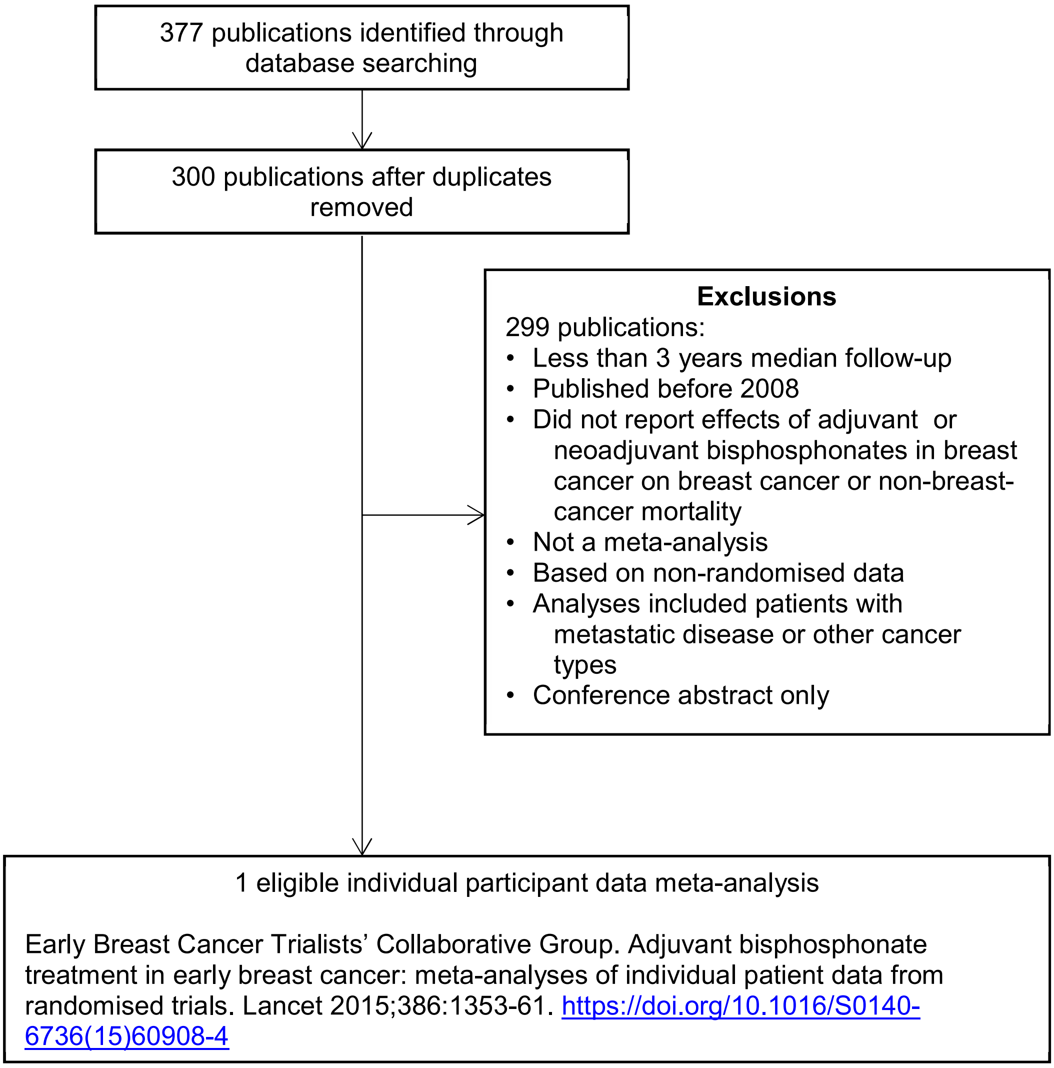

Supplemental Figure 13. Bisphosphonates in adjuvant or neoadjuvant breast cancer treatment: the process of study identification of *meta-analyses*

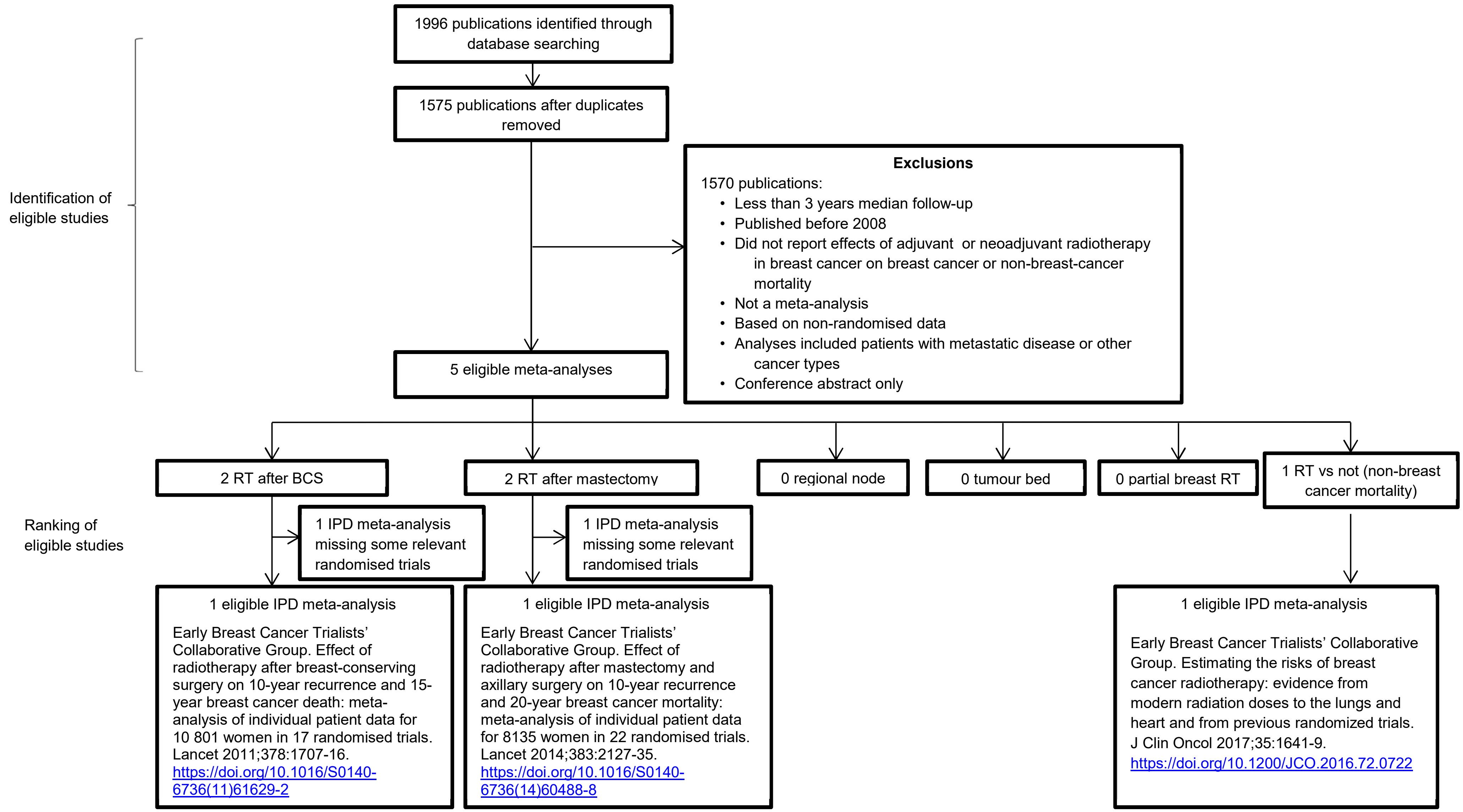

Abbreviations – BCS: breast conserving surgery, RT: radiotherapy, IPD: individual participant data

Supplemental Figure 14a. Radiotherapy in adjuvant or neoadjuvant breast cancer treatment: the process of study identification of *meta-analyses* *Continued on next page*

Continued from previous page

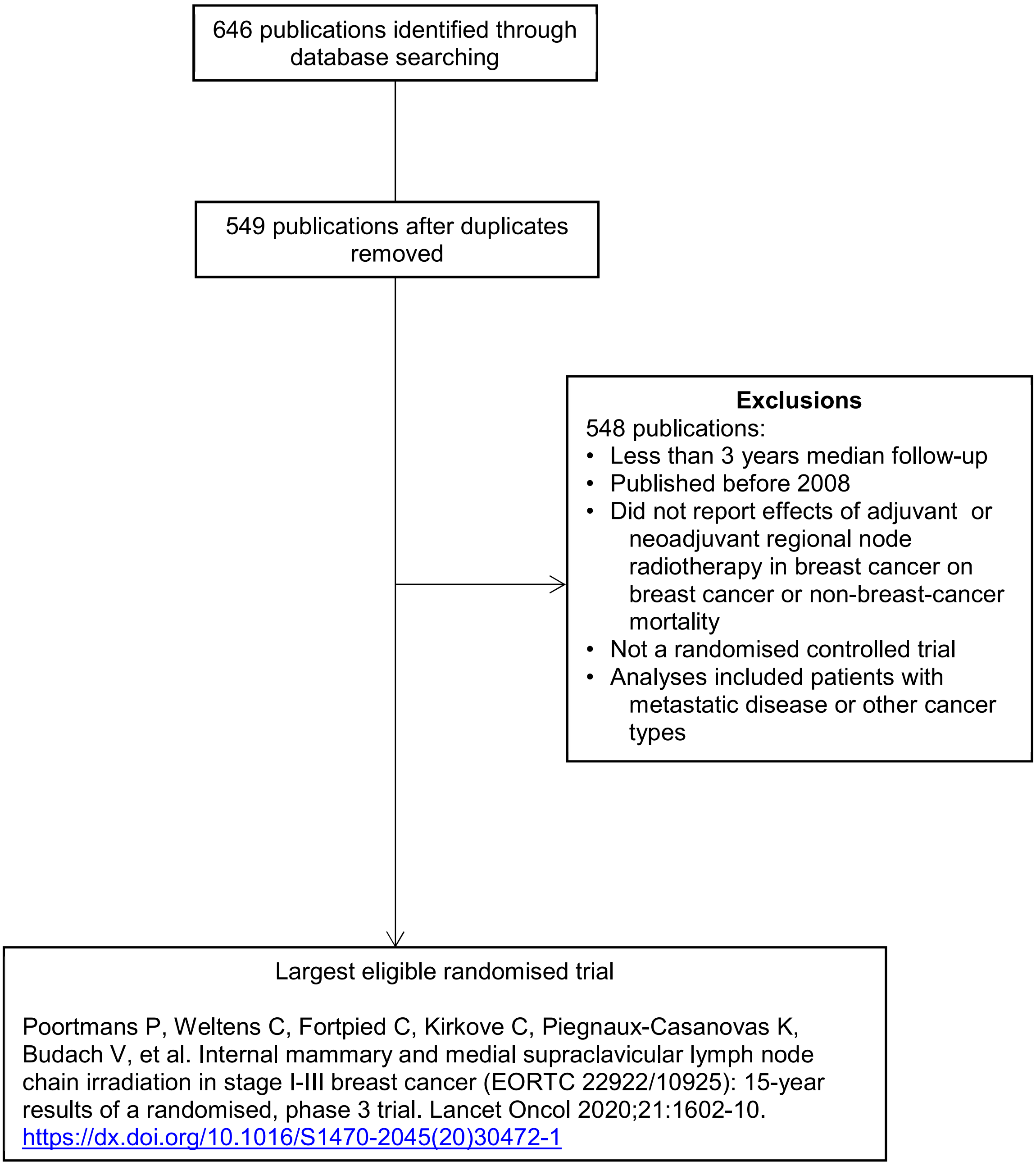

Supplemental Figure 14b.Regional node radiotherapy in adjuvant or neoadjuvant breast cancer treatment: the process of study identification of *randomised trials*

Continued from previous page

535 publications identified through database searching

Exclusions
534 publications:
- Less than 3 years median follow-up
- Published before 2008
- Did not report effects of adjuvant or neoadjuvant partial breast radiotherapy in breast cancer on breast cancer or non-breast-cancer mortality
- Not a randomised controlled trial
- Analyses included patients with metastatic disease or other cancer types
- Conference abstract only

Largest eligible randomised trial

Vicini FA, Cecchini RS, White JR, Arthur DW, Julian TB, Rabinovitch RA, et al. Long-term primary results of accelerated partial breast irradiation after breast-conserving surgery for early-stage breast cancer: a randomised, phase 3, equivalence trial. Lancet 2019;394:2155-64. https://doi.org/10.1016/S0140-6736(19)32514-0

Supplemental Figure 14c. Partial breast radiotherapy in adjuvant or neoadjuvant breast cancer treatment: the process of study identification of *randomised trials*

Continued on next page

Continued from previous page

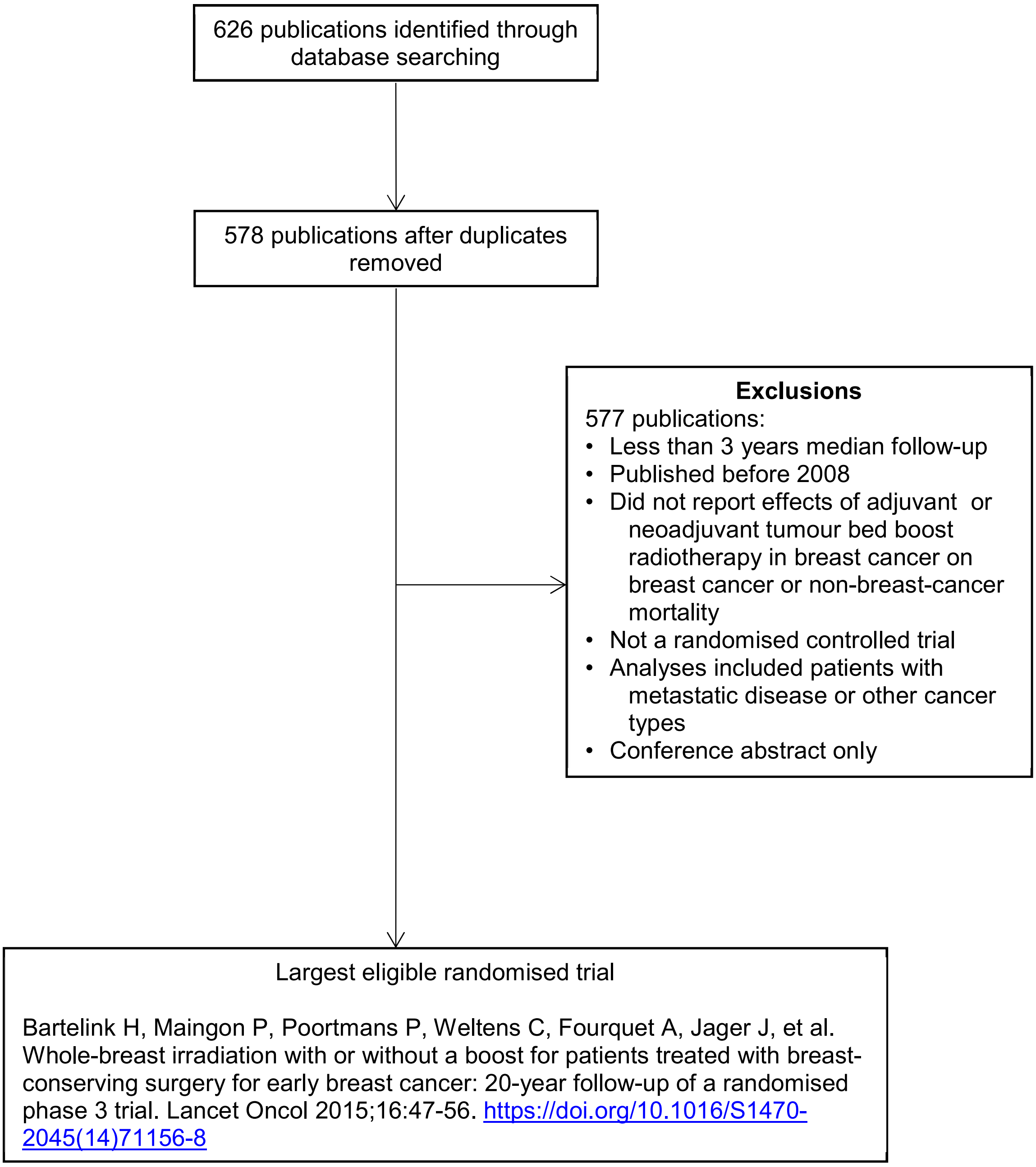

Supplemental Figure 14d. Tumour bed boost radiotherapy in adjuvant or neoadjuvant breast cancer treatment: the process of study identification of *randomised trials*

Supplemental Table 3. Risk ratios and p-values for studies where rate ratios were not reported

Risk ratios are estimated as $(\frac{a}{a+c})/(\frac{b}{b+d})$ where a, b, c, and d are numbers of individuals and defined as follows:

**Death from cause of interest**			
Yes		No	
Allocated to Treatment	Allocated to Control	Allocated to Treatment	Allocated to Control
a	b	c	d

For these trials in which rates are small and the treatments arms being compared have usually been followed for similar lengths of time risk ratios will be approximately equal to rate ratios. P-values are two-sided significance levels for testing whether the risk ratio is equal to 1, calculated using Fisher's exact method. Calculations were performed using 'csi' command in Stata/Se version 15.1.

**Treatment comparison**	**Reference**	**Page/Table/ Figure**	**Cause**	**a**	**b**	**c**	**d**	**Risk ratio**	**95% confidence interval**	**P value**
Any anthracycline chemotherapy vs no chemotherapy	EBCTCG 2012[1]	P442, Fig 6	Acute myeloid leukaemia	8	0	4746	4733	-	-	0.004
Taxane+anthracycline vs anthracycline	EBCTCG 2012[1]	P442, Fig 2	Acute myeloid leukaemia	11	1	22,117	22,122	11.00	(1.42-85.17)	0.003
Pertuzumab vs not	Piccart 2021[2]	Table 2	*Death post recurrence	86	108	2314	2296	0.80	(0.60-1.05)	0.06
			*Death without recurrence	39	39	2361	2365	1.00	(0.64-1.56)	0.54
		Table 3	Fatal adverse event	22	30	2342	2375	0.75	(0.43-1.29)	0.18
			Cardiac disorder	2	4	2362	2401	0.51	(0.09-2.77)	0.35
			Neoplasm benign, malignant and unspecified	13	17	2351	2388	0.78	(0.38-1.60)	0.31
			Nervous disorder	1	0	2363	2405	-	-	0.50

Continued on next page

Treatment comparison	Reference	Page/Table/ Figure	Cause	a	b	c	d	Risk ratio	95% confidence interval	P value
Trastuzumab emtansine vs trastuzumab	von Minckwitz 2019[3]	P621-2	*Death post recurrence	40	53	703	690	0.75	(0.51-1.12)	0.10
			*Death without recurrence	2	3	741	740	0.67	(0.11-3.98)	0.50
Neratinib versus not	Martin 2017[4]	Table 3	Death without recurrence	4	5	1416	1415	0.80	(0.22-2.97)	0.50
Ovarian suppression vs not (both with tamoxifen)	Francis 2018[5]	Fig 2, Table S3	*Deaths post recurrence	58	78	957	940	0.75	(0.54-1.04)	0.05
			*Deaths with no recurrence recorded	3	10	1012	1008	0.30	(0.08-1.09)	0.05
		Fig 2, Tables S2C, S3	Other primary cancers	2	2	1013	1016	1.00	(0.14-7.11)	0.69
			Myocardial infarction	0	2	1015	1016	-	-	0.25
			Cardiogenic shock	0	1	1015	1017	-	-	0.50
			Multiple sclerosis	0	1	1015	1017	-	-	0.50
			Mixed drug intoxication	0	1	1015	1017	-	-	0.50
			Unknown or incomplete information	1	3	1014	1015	0.33	(0.03-3.21)	0.31
Aromatase inhibitor vs tamoxifen (both with ovarian suppression)	EBCTCG 2022[6]	Appendix P16	Colorectal cancer	1	0	3527	3502	-	-	0.50
			Gastric cancer	1	0	3527	3502	-	-	0.50
			Head and neck cancer	1	0	3527	3502	-	-	0.50
			Skin cancer	2	0	3526	3502	-	-	0.25
			Brain cancer	0	1	3528	3501	-	-	0.50
			Primary liver cancer	0	1	3528	3501	-	-	0.50
			Uterine cancer	0	1	3528	3501	-	-	0.50
			Hepatic disease	1	0	3527	3502	-	-	0.50
			Diabetes	0	1	3528	3501	-	-	0.50
			Mixed drug intoxication	0	1	3528	3501	-	-	0.50
			Suicide	0	2	3528	3500	-	-	0.25
			Accident	0	1	3528	3501	-	-	0.50
			Infectious/parasitic	0	1	3528	3501	-	-	0.50

Continued on next page

Treatment comparison	Reference	Page/Table/ Figure	Cause	a	b	c	d	Risk ratio	95% confidence interval	P value
Aromatase inhibitor vs not (both after 5 years of tamoxifen)	Ingle 2008[7]	Table 3	Breast cancer mortality	58	70	2525	2517	0.83	(0.59-1.17)	0.16
			Non-breast-cancer mortality	96	85	2487	2502	1.13	(0.85-1.51)	0.22
			Other primary cancers	29	31	2554	2556	0.94	(0.57-1.55)	0.45
			Other known conditions	60	48	2523	2539	1.25	(0.86-1.82)	0.14
			All other causes	7	6	2576	2581	1.17	(0.39-3.47)	0.50
Extended aromatase inhibitor	Goss 2016[8]	Fig 1, P214	Breast deaths	31	34	928	925	0.91	(0.57-1.47)	0.40
			Non-breast deaths	69	66	890	893	1.05	(0.76-1.45)	0.43
			Other primary cancers	26	25	933	934	1.04	(0.61-1.79)	0.50
			Cardiovascular events	14	11	945	948	1.27	(0.58-2.79)	0.34
			Other causes	29	30	930	929	0.97	(0.58-1.60)	0.50
Extended aromatase inhibitor	Mamounas 2019[9]	P92	Breast deaths	46	47	1937	1936	0.98	(0.65-1.46)	0.50
			Non-breast deaths	118	99	1865	1884	1.19	(0.92-1.55)	0.10
		Appendix p7	Second primary cancer	38	36	1945	1947	1.06	(0.67-1.66)	0.45
			Cerebral haemorrhage	4	4	1979	1979	1.00	(0.25-3.99)	0.64
			Other central nervous system	4	3	1979	1980	1.33	(0.30-5.95)	0.50
			Ischaemic heart disease	5	6	1978	1977	0.83	(0.25-2.73)	0.50
			Heart failure	6	4	1977	1979	1.50	(0.42-5.31)	0.38
			Arrhythmia	2	1	1981	1982	2.00	(0.18-22.04)	0.50
			Renal disease	1	1	1982	1982	1.00	(0.06-15.98)	0.75
			Pulmonary disease	8	7	1975	1976	1.14	(0.42-3.15)	0.50
			Gastrointestinal or liver disease	1	6	1982	1977	0.17	(0.02-1.38)	0.06
			Infection	2	6	1981	1977	0.33	(0.07-1.65)	0.14
			Accidental death or suicide	2	0	1981	1983	-	-	0.25
			Adverse event not otherwise classified	4	1	1979	1982	4.00	(0.45-35.76)	0.19
			Cause unknown	41	24	1942	1959	1.71	(1.04-2.82)	0.02

Continued on next page

Treatment comparison	Reference	Page/Table/ Figure	Cause	a	b	c	d	Risk ratio	95% confidence interval	P value
Partial vs whole breast radiotherapy	Vicini 2019[10]	p2160	Breast cancer mortality	49	44	2044	1995	1.08	(0.73-1.62)	0.39

* Many papers report 'Death post recurrence' and 'Death without recurrence'. These are interpreted as 'Breast cancer deaths' and 'Non-breast-cancer deaths'
†One death was classified in both the nervous system and the injury, poisoning and procedural complications categories

References for Supplemental Table 3

1 Early Breast Cancer Trialists' Collaborative Group. Comparisons between different polychemotherapy regimens for early breast cancer: meta-analyses of long-term outcome among 100,000 women in 123 randomised trials. Lancet 2012;379:432-44. https://doi.org/10.1016/S0140-6736(11)61625-5

2 Piccart M, Procter M, Fumagalli D, de Azambuja E, Clark E, Ewer MS, et al. Adjuvant pertuzumab and trastuzumab in early HER2-positive breast cancer in the APHINITY trial: 6 years' follow-up. JCO 2021;39:1448-57. https://ascopubs.org/doi/10.1200/JCO.20.01204

3 von Minckwitz G, Huang CS, Mano MS, Loibl S, Mamounas EP, Untch M, et al. Trastuzumab Emtansine for Residual Invasive HER2-Positive Breast Cancer. N Eng J Med 2019;380:617-28. https://doi.org/10.1056/NEJMoa1814017

4 Martin M, Holmes FA, Ejlertsen B, Delaloge S, May B, Iwata H, et al. Neratinib after trastuzumab-based adjuvant therapy in HER2-positive breast cancer (ExteNET): 5-year analysis of a randomised, double-blind, placebo-controlled, phase 3 trial. Lancet Oncol 2017;18:1688-700. https://doi.org/10.1016/S1470-2045(17)30717-9

5 Francis PA, Pagani O, Fleming GF, Walley BA, Colleoni M, Láng I, et al. Tailoring adjuvant endocrine therapy for premenopausal breast cancer. N Eng J Med 2018;12;379:122-37. https://doi.org/10.1056/NEJMoa1803164

6 Early Breast Cancer Trialists' Collaborative Group. Aromatase inhibitors versus tamoxifen in premenopausal women with estrogen receptor positive early stage breast cancer treated with ovarian suppression: patient-level meta-analysis of 7,030 women in four randomised trials. Lancet Oncol 2022. https://doi.org/10.1016/S1470-2045(21)00758-0

7 Ingle JN, Tu D, Pater JL, Muss HB, Martino S, Robert NJ, et al. Intent-to-treat analysis of the placebo-controlled trial of letrozole for extended adjuvant therapy in early breast cancer: NCIC CTG MA.17. Ann Oncol 2008;19:877-82. https://doi.org/10.1093/annonc/mdm566

8 Goss PE, Ingle JN, Pritchard KI, Robert NJ, Muss H, Gralow J, et al. Extending Aromatase-Inhibitor Adjuvant Therapy to 10 Years. N Engl J Med 2016;375:209-19. https://www.nejm.org/doi/full/10.1056/NEJMoa1604700

9 Mamounas EP, Bandos H, Lembersky BC, Jeong JH, Geyer CE, Rastogi P, et al. Use of letrozole after aromatase inhibitor-based therapy in postmenopausal breast cancer (NRG Oncology/NSABP B-42): a randomised, double-blind, placebo-controlled, phase 3 trial. Lancet Oncol 2019;20:88-99. https://doi.org/10.1016/S1470-2045(18)30621-1

10 Vicini FA, Cecchini RS, White JR, Arthur DW, Julian TB, Rabinovitch RA, et al. Long-term primary results of accelerated partial breast irradiation after breast-conserving surgery for early-stage breast cancer: a randomised, phase 3, equivalence trial. Lancet 2019;394:2155-64. https://doi.org/10.1016/S0140-6736(19)32514-0

Supplemental Table 4. Calendar periods when adjuvant and neoadjuvant breast cancer treatments were first explicitly described in clinical guidelines

	Guidelines				
	**NCCN**	**ASCO/ASTRO**	**ESMO**	**St Gallen**	**NICE**
Year guidelines started	1996	1993	1999	1988	2002
			**Five-year period treatment first included**		
**Chemotherapy**					
Anthracycline-based	-	-	2001-2005	-	2001-2005
Taxane-based	2001-2005	-	2001-2005	2001-2005	-
Platinum-based (neoadjuvant)	2011-2015	2016-2021	2011-2015		2016-2021
Capecitabine after neoadjuvant chemotherapy	2016-2021	2016-2021	2016-2021	2016-2021	-
**Anti HER2 therapy**					
Trastuzumab	2006-2010	-	2006-2010	2006-2010	-
Pertuzumab	2016-2021	2016-2021	2016-2021	2016-2000	2016-2021
Trastuzumab emtansine	2016-2021	2016-2021	2016-2021	2016-2000	2016-2021
Neratinib	2016-2021	2016-2021	2016-2021	2016-2000	2016-2021
**Endocrine therapy in ER+ disease**					
Tamoxifen up to 5 yr*	-	-	-	-	-
Extended endocrine therapy	-	2011-2015	2011-2015	2011-2015	-
Ovarian suppression or ablation	-	2016-2021	2001-2005	1991-1995	2001-2005
Aromatase inhibitor	2001-2005	2001-2005	2006-2010	2006-2010	-
**Bisphosphonates**					
Bisphosphonates	2016-2021	2016-2021	2016-2021	2016-2021	2016-2021
**Radiotherapy**					
Whole breast RT*	-	-	-	-	-
Partial breast RT	2016-2021	2016-2021	2011-2015	2016-2021	2016-2021
Tumour bed boost	-	-	2006-2010	2001-2005	-
Chest wall RT*	-	-	-	-	-
Regional node RT†	2016-2021	2016-2021	2016-2021	2016-2021	2016-2021

Abbreviations NCCN National Comprehensive Cancer Network; ASCO American Society of Clinical Oncology; ASTRO American Society for Radiation Oncology; ESMO European Society of Medical Oncology; St Gallen International Consensus Guidelines; NICE National Institute for Health and Care Excellence; RT Radiotherapy.

*Treatment in widespread clinical use since before 1990.
† Treatment in widespread but variable clinical use since before 1990. Guidelines refer to recommendations for comprehensive regional RT including the internal mammary nodes given with radiotherapy to the breast or chest wall.

Absence of information in the table (-) may reflect varying methodologies for guideline development, timings of updates, non-availability of old guidelines and differing levels of detail. It does not mean that treatments were unavailable or not recommended. Some older treatments, e.g. whole breast radiotherapy, were adopted into widespread use before clinical guidelines were available (Figure 2). In general, the more recent the treatment, the greater the certainty as to when it was introduced into guidelines.

Treatments only recommended in the USA by the end of 2021 (pembrolizumab and abemaciclib) were not included.

NCCN guidelines on all current adjuvant and neoadjuvant breast cancer treatments are accessible on their website and in the Journal of the National Comprehensive Cancer Network (JNCCN) and they are updated at least annually. Outdated guidance is removed from the website, but some older versions of NCCN treatment guidelines are available in the JNCCN.

ASCO/ASTRO produce summaries of guidance on specific treatment categories. These are published when new evidence is generated. Both current and previous summaries are available.

ESMO produce clinical practice guidelines which are updated when there is need, as judged by the ESMO Guidelines Committee. Both current and previous guidelines are available.

St Gallen produce summaries of guidance on all adjuvant breast cancer treatments after each (usually bi-annual) meeting. Both recent and previous summaries are available.

NICE guidelines on adjuvant breast cancer treatments are published on their website but not elsewhere. Outdated guidelines are removed from the website but are accessible via the National Archives (https://webarchive.nationalarchives.gov.uk). Some technology appraisals relating to individual treatments are also produced. These may be updated or removed in the light of new evidence.

References for Supplemental Table 4

NCCN	2001-2005	Trauth D, Goldstein LJ. Taxanes in the Adjuvant Treatment of Breast Cancer. J Natl Compr Canc Netw 2003;1:222-31. https://doi.org/10.6004/jnccn.2003.0021 (*review article with guideline recommendation*)
		Carlson RW, Edge SB, McCormick B, Theriault R. Clinical Practice Guidelines in Oncology version 1.2005. National Comprehensive Cancer Network. Available at http://www.nccn.org.
	2006-2010	Carlson RW, Brown E, Burstein HJ, Gradishar WJ, Hudis CA, Loprinzi C, et al. NCCN Task Force Report: adjuvant therapy for breast cancer J Natl Compr Canc Netw 2006;4:S1.
	2016-2021	National Comprehensive Cancer Network Guidelines Version 6.2020 Breast Cancer. https://www.nccn.org/ Accessed 7th Nov 2020
		National Comprehensive Cancer Network Guidelines Version 2.2022 Breast Cancer. https://www.nccn.org/ 22nd Feb 2022
ASCO Chemo/targeted	2016-2021	Denduluri N, Chavez-MacGregor M, Telli ML, Eisen A, Graff SL, Hassett MJ, et al. Selection of optimal adjuvant chemotherapy and targeted therapy for early breast cancer: ASCO clinical practice guideline focused update. J Clin Oncol 2018;36:2433-43. https://doi.org/10.1200/JCO.2018.78.8604
ASCO Endocrine	2001-2005	Winer EP, Hudis C, Burstein HJ, Wolff AC, Pritchard KI, Ingle JN, et al. American Society of Clinical Oncology technology assessment on the use of aromatase inhibitors as adjuvant therapy for postmenopausal women with hormone receptor–positive breast cancer: status report 2004. J Clin Oncol 2005;23:619-29. https://doi.org/10.1200/JCO.2005.09.121
	2011-2015	Burstein HJ, Temin S, Anderson H, Buchholz TA, Davidson NE, Gelmon KE, et al. Adjuvant endocrine therapy for women with hormone receptor–positive breast cancer: American Society of Clinical Oncology clinical practice guideline focused update. J Clin Oncol 2014;32:2255. https://doi.org/10.1200/JCO.2013.54.2258
	2016-2021	Burstein HJ, Lacchetti C, Anderson H, Buchholz TA, Davidson NE, Gelmon KA, et al. Adjuvant Endocrine Therapy for Women With Hormone Receptor–Positive Breast Cancer: ASCO Clinical Practice Guideline Focused Update. J Clin Oncol 2019;37:423-38. https://doi.org/10.1200/JCO.18.01160
ASCO Bisphos	2016-2021	Dhesy-Thind S, Fletcher GG, Blanchette PS, Clemons MJ, Dillmon MS, Frank ES, et al. Adjuvant Bisphosphonates and Other Bone-Modifying Agents in Breast Cancer. J Clin Oncol 2017;35:2062-81. https://doi.org/10.1200/JCO.2016.70.7257
ASTRO Whole breast RT	2016-2021	Smith BD, Bellon JR, Blitzblau R, Freedman G, Haffty B, Hahn C, et al. Radiation therapy for the whole breast: Executive summary of an American Society for Radiation Oncology (ASTRO) evidence-based guideline. Pract Radiat Oncol 2018;8:145-52. https://doi.org/10.1016/j.prro.2018.01.012
ASTRO Partial breast RT	2016-2021	Correa C, Harris EE, Leonardi MC, Smith BD, Taghian AG, Thompson AM, et al. Accelerated Partial Breast Irradiation: Executive summary for the update of an ASTRO Evidence-Based Consensus Statement. Pract Radiat Oncol 2017;7:73-9. https://doi.org/10.1016/j.prro.2016.09.007
ASTRO Postmastectomy RT	2001-2005	Recht A, Edge SB, Solin LJ, Robinson DS, Estabrook A, Fine RE, Fleming GF, Formenti S, Hudis C, Kirshner JJ, Krause DA. Postmastectomy radiotherapy: clinical practice guidelines of the American Society of Clinical Oncology. Journal of clinical oncology. 2001 Mar 1;19(5):1539-69. https://doi.org/10.1200/JCO.2001.19.5.1539

	2016-2021	Recht A, Comen EA, Fine RE, Fleming GF, Hardenbergh PH, Ho AY, et al. Postmastectomy Radiotherapy: An American Society of Clinical Oncology, American Society for Radiation Oncology, and Society of Surgical Oncology Focused Guideline Update. J Clin Oncol 2016;34:4431-42. https://doi.org/10.1016/j.prro.2016.08.009
ESMO	2001-2005	Pestalozzi BC, Luporsi-Gely E, Jost LM, Bergh J. ESMO Minimum Clinical Recommendations for diagnosis, adjuvant treatment and follow-up of primary breast cancer. Ann Oncol 2005;16 Suppl 1:i7-9. https://doi.org/10.1093/annonc/mdi825
	2006-2010	Aebi S, Davidson T, Gruber G, Castiglione M, ESMO Guidelines Working Group. Primary breast cancer: ESMO Clinical Practice Guidelines for diagnosis, treatment and follow-up. Ann Oncol 2010;21 Suppl 5:v9-14. https://doi.org/10.1093/annonc/mdq159
	2011-2015	Senkus E, Kyriakides S, Penault-Llorca F, Poortmans P, Thompson A, Zackrisson S, et al. ESMO Guidelines Working Group. Primary breast cancer: ESMO Clinical Practice Guidelines for diagnosis, treatment and follow-up. Ann Oncol 2013;24: Suppl 6:vi7-23. https://doi.org/10.1093/annonc/mdt284
	2011-2015	Senkus E, Kyriakides S, Ohno S, Penault-Llorca F, Poortmans P, Rutgers E, et al. Primary breast cancer: ESMO Clinical Practice Guidelines for diagnosis, treatment and follow-up. Ann Oncol 2015:26 Suppl 5:v8-30. https://doi.org/10.1093/annonc/mdv298
	2016-2021	Cardoso F, Kyriakides S, Ohno S, Penault-Llorca F, Poortmans P, Rubio IT, et al. On behalf of the ESMO Guidelines Committee. Early breast cancer: ESMO Clinical Practice Guidelines for diagnosis, treatment and follow-up. Ann Oncol, 2019;30:1194–220. https://doi.org/10.1093/annonc/mdz173
St Gallen	1991-1995	Goldhirsch A, Wood WC, Senn HJ, Glick JH, Gelber RD. Meeting highlights: international consensus panel on the treatment of primary breast cancer. JNCI 1995;87:1441-5. https://doi.org/10.1093/jnci/90.21.1601
	2001-2005	Goldhirsch A, Wood WC, Gelber RD, Coates AS, Thurlimann B, Senn HJ. Meeting highlights: updated international expert consensus on the primary therapy of early breast cancer. J Clin Oncol 2003;21:3357-65.2001-2005. https://doi.org/10.1200/JCO.2003.04.576
		Goldhirsch A, Glick JH, Gelber RD, Coates AS, Thürlimann B, Senn HJ. Meeting highlights: international expert consensus on the primary therapy of early breast cancer 2005. Ann Oncol 2005;16:1569-83. https://doi.org/10.1093/annonc/mdi326
	2006-2010	Goldhirsch A, Coates AS, Gelber RD, Glick JH, Thürlimann B, Senn HJ. First—select the target: better choice of adjuvant treatments for breast cancer patients. Ann Oncol 2006;17:1772-6. https://doi.org/10.1093/annonc/mdl398
	2011-2015	Goldhirsch A, Winer EP, Coates AS, Gelber RD, Piccart-Gebhart M, Thürlimann B, et al. Personalizing the treatment of women with early breast cancer: highlights of the St Gallen International Expert Consensus on the Primary Therapy of Early Breast Cancer 2013. Ann Oncol 2013;24:2206-23. https://doi.org/10.1093/annonc/mdt303
	2016-2021	Burstein HJ, Curigliano C, Loibl S, Dubsky P, Gnant M, Poortmans P et al. Estimating the benefits of therapy for early-stage breast cancer: the St. Gallen International Consensus Guidelines for the primary therapy of early breast cancer 2019. Ann Oncol 2019;30:1541–57. https://doi.org/10.1093/annonc/mdz235
		Burstein HJ, Curigliano G, Thürlimann B, Weber WP, Poortmans P, Regan MM, et al. Customizing local and systemic therapies for women with early breast cancer: the St. Gallen International Consensus Guidelines for treatment of early breast cancer 2021. Ann Oncol 2021; 32:1216-35. https://doi.org/10.1016/j.annonc.2021.06.023
NICE	2001-2005	https://www.nice.org.uk/guidance/csg1/resources/improving-outcomes-in-breast-cancer-update-773371117. Accessed 22nd Feb 2022
	2016-2021	NICE (National Institute for Health and Care Excellence) guideline. Early and locally advanced breast cancer: diagnosis and management, 2018. http://www.nice.org.uk/guidance/ng101. Accessed 26th Nov 2020
		Pertuzumab for adjuvant treatment of HER2-positive early stage breast cancer. NICE Technology Appraisal Guidance TA569, 2019. https://www.nice.org.uk/guidance/ta569 Accessed 7th Nov 2020

Trastuzumab emtansine for adjuvant treatment of HER2-positive early breast cancer. NICE Technology Appraisal Guidance TA632, 2020. https://www.nice.org.uk/guidance/ta632. Accessed 7th Nov 2020

Neratinib for extended adjuvant treatment of hormone receptor-positive, HER2-positive early stage breast cancer after adjuvant trastuzumab. NICE Technology Appraisal Guidance TA612, 2019 https://www.nice.org.uk/guidance/ta612. Accessed 7th Nov 2020

Supplemental Table 5. Rate ratios for the effects of adjuvant and neoadjuvant breast cancer treatments on breast cancer and non-breast-cancer mortality.

Trial descriptions, and page, table, and figure numbers in references (see Table 2 in main text)

**Treatment type** *Patient group*	**Treatment comparison**	**Reference**	**Time-period studied***	**Number of women†**	**Breast cancer mortality RR (95% CI)**	**Table/ Figure/ Page**	**Non-breast-cancer mortality RR (95%CI)**	**Table/ Figure/ Page**
**Chemotherapy**								
All women	Anthracycline *vs* no chemotherapy‡	EBCTCG 2012[1]	10 years	8,575	0.79 (0.72-0.85)	Fig. 5, P439	1.20 (1.00-1.43)	Appendix P37
	Taxane+anthracycline *vs* anthracycline§	EBCTCG 2012[1]	8 years	11,167 breast<br>44,251 non breast	0.86 (0.79-0.93)	Fig. 1, P434	0.99 (0.83-1.15)	Appendix P25
ER-PR- HER2-	Platinum vs other chemotherapy (neoadjuvant)	Poggio 2018[2]	<1 year (path CR)	2,109	Not reported	-	Not reported	-
HER2- residual cancer after neoadjuvant chemotherapy	Capecitabine *vs* no chemotherapy	Masuda 2017[3]	4 years	910	Not reported	-	Not reported	-
**Anti HER2 therapy** *(HER2 positive cancer)*								
	Trastuzumab+chemotherapy *vs* chemotherapy alone	EBCTCG 2021[4]	10 years	13,864	0.67 (0.61-0.73)	Fig. 2B	0.90 (0.72-1.12)	Fig. 2C
High risk	Pertuzumab+trastuzumab *vs* trastuzumab alone (both after standard chemotherapy)	Piccart 2021[5]	6 years	4,804	0.80 (0.60-1.05)¶	Table 2	1.00 (0.64-1.56)¶	Table 2
Residual cancer after neoadjuvant chemotherapy and anti HER2 therapy	Trastuzumab emtansine *vs* trastuzumab (both after neoadjuvant therapy including taxane & trastuzumab)	von Minckwitz 2019[6]	3 years	1,486	0.75 (0.51-1.12)¶	P621-2	0.67 (0.11-3.98)¶	P621-2
ER+, after trastuzumab	Neratinib *vs* not (both after neoadjuvant and/or adjuvant chemotherapy +trastuzumab)	Martin 2017[7]	5 years	2,840	Not reported	-	0.80 (0.22-2.97)¶	Table 3, P1695
**Neoadjuvant timing of systemic therapy**								
Neoadjuvant versus adjuvant chemotherapy		EBCTCG 2018[8]	15 years	4,756	1.06 (0.95-1.18)	Fig. 2C	0.94 (0.73-1.22)	Appendix P7

Continued on next page

Continued from previous page

Treatment type<br>*Patient group*	Treatment comparison	Reference	Time-period Studied*	Number of women†	Breast cancer mortality RR (95% CI)	Table/ Figure/ Page	Non-breast-cancer mortality RR (95%CI)	Table/ Figure/ Page
**Endocrine therapy in ER+ disease**								
Any age	Tamoxifen for about 5 years *vs* no tamoxifen	EBCTCG 2011a[9]	15 years	10,645	0.70 (0.64-0.75)	Fig. 5	1.02 (0.90-1.14)	Table 1
	Extended tamoxifen for ≥10 years *vs* 5 years tamoxifen	Ibrahim 2017[10]	≥5 years**	14,281	0.88 (0.80-0.97)**	Fig 3	Not reported††	-
Pre-menopausal	Ovarian suppression for 5 years *vs* no ovarian suppression (both with tamoxifen)	Francis 2018[11]	8 years	2,033	0.75 (0.54-1.04)¶	Table S3, Fig S1	0.30 (0.08-1.09)¶	Table S3, Fig S1
	AI for 3 or 5 years *vs* tamoxifen for 3 or 5 years (both with ovarian suppression)	EBCTCG 2022[12]	10 years	7,030	1.01 (0.82-1.24)	Fig. 1C	1.30 (0.75-2.25)	Fig. 4
Post-menopausal	AI for *5 years vs* tamoxifen for *5 years*	EBCTCG 2015a[13]	10 years	9,885	0.85 (0.75-0.96)	Fig. 1B	0.94 (0.82-1.07)	Fig. 1C
	Tamoxifen for 2-3 years then AI up to year 5 *vs* tamoxifen for 5 years	EBCTCG 2015a[13]	8 years‡‡	11,798	0.84 (0.72-0.96)	Fig 3B	0.79 (0.67-0.93)	Fig 3C
	AI for 5 years vs no AI (both after 5 years of tamoxifen)	Ingle 2008[14]	4 years	5,170	0.83 (0.59-1.17)¶	Table 3	1.13 (0.85-1.51)¶	Table 3
	Extended AI for 5 years vs not (after ~10 years endocrine therapy with tamoxifen then AI)	Goss 2016[15]	10 years§§	1,918	0.91 (0.57-1.47)¶	P214	1.05 (0.76-1.45)¶	P214
	Extended AI for 5 years vs not (after 5 years endocrine therapy including at least 2 years AI)	Mamounas 2019[16]	7 years¶¶	3,966	0.98 (0.65-1.46)¶	P92,Table S2	1.19 (0.92-1.55)¶	P92, Table S2
**Bisphosphonates**								
Post-menopausal	Bisphosphonate *vs* no bisphosphonate (duration varied from one dose to 5 years)	EBCTCG 2015b[17]	10 years	11,767 breast<br>18,766 non-breast	0.82 (0.73-0.93)	Fig. 3, Panel F	0.99 (0.82-1.19)***	Appendix P4

Continued on next page

Continued from previous page

**Treatment category** *Patient group*	**Treatment comparison**	**Reference**	**Time-period Studied***	**Number of women†**	**Breast cancer mortality RR (95% CI)**	**Table/ Figure/ Page**	**Non-breast-cancer mortality RR (95%CI)**	**Table/ Figure/ Page**
**Radiotherapy**								-
After breast-conserving surgery	Whole breast RT (sometimes with regional node and/or tumour bed boost) vs no RT	EBCTCG 2011b[18]	15 years	10,801	0.82 (0.75-0.90)	Fig. 1	Not reported	
	Partial breast RT *vs* whole breast RT (sometimes with tumour bed boost)	Vicini 2019[19]	10 years	4,132†††	1.08 (0.73-1.62)¶	P2160, Fig.3C	Not reported	-
	Tumour bed boost *vs* no boost (after whole breast RT)	Bartelink 2015[20]	20 years	5,318	1.01 (0.86-1.20)	P52	Not reported	-
After mastectomy and axillary dissection, node positive cancer	Chest wall and regional node RT *vs* no radiotherapy	EBCTCG 2014[21]	20 years	3,131	0.84 (0.76-0.94)	Fig. 2 Panel F	Not reported	-
Any surgery, target or nodes	Regional node RT *vs* no regional node RT (after breast conserving surgery or mastectomy, sometimes with breast/chest wall RT in both arms)	Poortmans 2020[22]	15 years	4,004	0.81 (0.70-0.94)	Fig. 4	1·13 (0·91–1·40)	P1606
Any surgery, target or nodes	RT *vs* no RT‡‡‡	EBCTCG 2017[23]	20 years	40,781	Not reported	-	1.15 (1.09-1.22)	Table 2

Abbreviations: RR rate ratio; CI confidence interval; *vs* versus; HER2 human epidermal growth factor 2; ER oestrogen receptor; PR progesterone receptor; path CR pathological complete response; AI aromatase inhibitor; RT radiotherapy

* The time period following diagnosis that the RRs relate to. In most studies, this starts soon after time of diagnosis, as randomisation took place soon after diagnosis. For studies, where randomisation did not take place until several years after diagnosis, both time from diagnosis to randomisation and time studied following randomisation are given in footnotes.

† The number of women was the same for assessment of breast cancer mortality and non-breast-cancer mortality unless indicated.

‡ Anthracycline breast cancer mortality rate ratio is for four or more cycles of any anthracycline regimen e.g. 4AC (doxorubicin and cyclophosphamide) *versus* no chemotherapy. The non-breast-cancer mortality rate ratio is for any anthracycline chemotherapy *versus* no chemotherapy.

§ Taxane+anthracycline *vs* anthracycline breast cancer mortality rate ratio is for the addition of four taxane cycles to anthracycline-based chemotherapy (usually 4AC). A RR for taxane+anthracycline *vs* no chemotherapy can be derived by multiplying RRs for anthracycline *vs* nil and taxane+anthracycline *vs* nil, i.e. 0.79 x 0.86=0.68 (95% CI 0.59-0.77). The non-breast-cancer mortality rate ratio is for taxane+anthracycline *vs* the same or more anthracycline-based non-taxane chemotherapy.

¶ Rate ratio not published. Values shown are risk ratios calculated from published data, see Supplemental Table 3 for details.

**Meta-analysis of 4 published trials. All women received 5 years of tamoxifen before randomisation. Median follow-up after randomisation varied from 4.2-7.6 years. Reported measure of reduction in breast cancer mortality is odds ratio.

†† In the two largest trials, the RRs for non-breast-cancer mortality were 0.99 (95% CI 0.89-1.10) (ATLAS) and 0.94 (0.82-1.07) (aTTom).

‡‡ Treatments diverged 2-3 years after diagnosis. Time-period studied is 8 years starting at 2 years after diagnosis.

§§ Women randomised after around 5 years of AI preceded by 5 years of tamoxifen. Time-period studied is 10 years starting at randomisation.

¶¶Women received about 5 years of AI or of tamoxifen→AI before randomisation. Time-period studied is 7 years starting at randomisation.

*** Includes women of all ages.

††† 76% of patients had invasive breast cancer and 24% had ductal carcinoma in situ.

‡‡‡ Includes all trials of RT versus no RT and also trials of RT versus more extensive surgery.

References for Supplemental Table 5

1 Early Breast Cancer Trialists' Collaborative Group. Comparisons between different polychemotherapy regimens for early breast cancer: meta-analyses of long-term outcome among 100,000 women in 123 randomised trials. Lancet 2012;379:432-44. https://doi.org/10.1016/S0140-6736(11)61625-5

2 Poggio F, Bruzzone M, Ceppi M, Ponde NF, La Valle G, Del Mastro L, et al. Platinum-based neoadjuvant chemotherapy in triple-negative breast cancer: a systematic review and meta-analysis. Ann Oncol 2018; 29: 1497-508. https://doi.org/10.1093/annonc/mdy127

3 Masuda N, Lee SJ, Ohtani S, Im YH, Lee ES, Yokota I, et al. Adjuvant Capecitabine for Breast Cancer after Preoperative Chemotherapy. N Eng J Med; 2017;376:2147-59. https://doi.org/10.1056/NEJMoa1612645

4 Early Breast Cancer Trialists' Collaborative Group. Trastuzumab for early-stage, HER2-positive breast cancer: a meta-analysis of 13,864 women in seven randomised trials. Lancet Oncol 2021;22:1139-50. https://doi.org/10.1016/S1470-2045(21)00288-6

5 Piccart M, Procter M, Fumagalli D, de Azambuja E, Clark E, Ewer MS, et al. Adjuvant pertuzumab and trastuzumab in early HER2-positive breast cancer in the APHINITY trial: 6 years' follow-up. JCO 2021;39:1448-57. https://www.nejm.org/doi/full/10.1056/NEJMoa1703643

6 Von Minckwitz G, Huang CS, Mano MS, Loibl S, Mamounas EP, Untch M, et al. Trastuzumab Emtansine for Residual Invasive HER2-Positive Breast Cancer. N Eng J Med 2019;380:617-28. https://doi.org/10.1056/NEJMoa1814017

7 Martin M, Holmes FA, Ejlertsen B, Delaloge S, May B, Iwata H, et al. Neratinib after trastuzumab-based adjuvant therapy in HER2-positive breast cancer (ExteNET): 5-year analysis of a randomised, double-blind, placebo-controlled, phase 3 trial. Lancet Oncol 2017;18:1688-700. https://doi.org/10.1016/S1470-2045(17)30717-9

8 Early Breast Cancer Trialists' Collaborative Group. . Long-term outcomes for neoadjuvant versus adjuvant chemotherapy in early breast cancer: meta-analysis of individual patient data from ten randomised trials. Lancet Oncol 2018; 19: 27-39. https://doi.org/10.1016/S1470-2045(17)30777-5

9 Early Breast Cancer Trialists' Collaborative Group. Relevance of breast cancer hormone receptors and other factors to the efficacy of adjuvant tamoxifen: patient-level meta-analysis of randomised trials. Lancet 2011a;378:771-84. https://doi.org/10.1016/S0140-6736(11)60993-8

10 Ibrahim EM, Al-Hajeili MR, Bayer AM, Abulkhair OA, Refae AA. Extended adjuvant endocrine therapy in early breast cancer: a meta-analysis of published randomized trials. Med Onc 2017;34:131. https://doi.org/10.1007/s12032-017-0986-2

11 Francis PA, Pagani O, Fleming GF, Walley BA, Colleoni M, Láng I, et al. Tailoring adjuvant endocrine therapy for premenopausal breast cancer. N Eng J Med 2018;12;379:122-37. https://doi.org/10.1056/NEJMoa1803164

12 Early Breast Cancer Trialists' Collaborative Group. Aromatase inhibitors versus tamoxifen in premenopausal women with estrogen receptor positive early stage breast cancer treated with ovarian suppression: patient-level meta-analysis of 7,030 women in four randomised trials. Lancet Oncol 2022. https://doi.org/10.1016/S1470-2045(21)00758-0

13 Early Breast Cancer Trialists' Collaborative Group. Aromatase inhibitors versus tamoxifen in early breast cancer: patient-level meta-analysis of the randomised trials. Lancet 2015a;386:1341-52. https://doi.org/10.1016/S0140-6736(15)61074-1

14 Ingle JN, Tu D, Pater JL, Muss HB, Martino S, Robert NJ, et al. Intent-to-treat analysis of the placebo-controlled trial of letrozole for extended adjuvant therapy in early breast cancer: NCIC CTG MA.17. Ann Oncol 2008;19:877-82. https://doi.org/10.1093/annonc/mdm566

15 Goss PE, Ingle JN, Pritchard KI, Robert NJ, Muss H, Gralow J, et al. Extending Aromatase-Inhibitor Adjuvant Therapy to 10 Years. N Engl J Med 2016;375:209-19. https://www.nejm.org/doi/full/10.1056/NEJMoa1604700

16 Mamounas EP, Bandos H, Lembersky BC, Jeong JH, Geyer CE, Rastogi P, et al. Use of letrozole after aromatase inhibitor-based therapy in postmenopausal breast cancer (NRG Oncology/NSABP B-42): a randomised, double-blind, placebo-controlled, phase 3 trial. Lancet Oncol 2019;20:88-99. https://doi.org/10.1016/S1470-2045(18)30621-1

17 Early Breast Cancer Trialists' Collaborative Group. Adjuvant bisphosphonate treatment in early breast cancer: meta-analyses of individual patient data from randomised trials. Lancet 2015b;386:1353-61. https://doi.org/10.1016/S0140-6736(15)60908-4

18 Early Breast Cancer Trialists' Collaborative Group. Effect of radiotherapy after breast-conserving surgery on 10-year recurrence and 15-year breast cancer death: meta-analysis of individual patient data for 10,801 women in 17 randomised trials. Lancet 2011b;378:1707-16. https://doi.org/10.1016/S0140-6736(11)61629-2

19 Vicini FA, Cecchini RS, White JR, Arthur DW, Julian TB, Rabinovitch RA, et al. Long-term primary results of accelerated partial breast irradiation after breast-conserving surgery for early-stage breast cancer: a randomised, phase 3, equivalence trial. Lancet 2019;394:2155-64. https://doi.org/10.1016/S0140-6736(19)32514-0

20 Bartelink H, Maingon P, Poortmans P, Weltens C, Fourquet A, Jager J, et al. Whole-breast irradiation with or without a boost for patients treated with breast-conserving surgery for early breast cancer: 20-year follow-up of a randomised phase 3 trial. Lancet Oncol 2015;16:47-56. https://doi.org/10.1016/S1470-2045(14)71156-8

21 Early Breast Cancer Trialists' Collaborative Group. Effect of radiotherapy after mastectomy and axillary surgery on 10-year recurrence and 20-year breast cancer mortality: meta-analysis of individual patient data for 8135 women in 22 randomised trials. Lancet 2014;383:2127-35. https://doi.org/10.1016/S0140-6736(14)60488-8

22 Poortmans P, Weltens C, Fortpied C, Kirkove C, Peignaux-Casasnovas K, Budach V, et al. Internal mammary and medial supraclavicular lymph node chain irradiation in stage I-III breast cancer (EORTC 22922/10925): 15-year results of a randomised, phase 3 trial. Lancet Oncol 2020;21:1602-10. https:// 10.1016/S1470-2045(20)30472-1

23 Early Breast Cancer Trialists' Collaborative Group. Estimating the Risks of Breast Cancer Radiotherapy: Evidence From Modern Radiation Doses to the Lungs and Heart and From Previous Randomized Trials. J Clin Oncol 2017;35:1641-49. https://doi.org/10.1200/JCO.2016.72.0722

Supplemental Table 6. Rate ratios for the effects of adjuvant and neoadjuvant breast cancer treatments not at present reported to reduce breast cancer mortality (see Table 2 in main text)

Treatment type<br>*Patient group*	Treatment comparison	Reference	Time-period studied*	Number of women	Breast cancer recurrence RR (95% CI)	Ipsilateral breast tumour recurrence RR (95% CI)
**Chemotherapy**						
ER-PR- HER2-	Platinum (neoadjuvant)	Poggio 2018[1]	<1 year (path CR)	2,109	Not reported†	Not reported
HER2- residual cancer after neoadjuvant chemotherapy	Capecitabine *vs* not	Masuda 2017[2]	4 years	910	0.70 (0.53 - 0.92)‡	Not reported
**Anti HER2 therapy** *(HER2 positive cancer)*						
High risk	Pertuzumab *vs* not	Piccart 2021[3]	6 years	4,804	0.76 (0.64-0.91)§	Not reported
Residual cancer after neoadjuvant chemotherapy and anti HER2 therapy	Trastuzumab emtansine *vs* trastuzumab	von Minckwitz 2019[4]	3 years	1,486	0.50 (0.39-0.64)§	Not reported
ER+ after adjuvant trastuzumab	Neratinib *vs* not	Martin 2017[5]	5 years	2,840	0.73 (0.57-0.92)¶	Not reported
**Neoadjuvant timing of systemic therapy**						
Neoadjuvant versus adjuvant chemotherapy		EBCTCG 2018[6]	15 years	4,756	Local 1.37 (1.17-1.61)<br>Distant 1.02 (0.92-1.14)	Not reported
**Endocrine therapy in ER+ disease**						
Pre-menopausal	Ovarian suppression *vs* not (both with tamoxifen)<br>*5 years*	Francis 2018[7]	8 years	2,033	0.76 (0.62-0.93)**	Not reported
	AI *vs* tamoxifen (both with ovarian suppression)<br>*3 or 5 years*	EBCTCG 2022[8]	10 years	7.030	0.79 (0.69-0.90)††	Not reported
Post-menopausal	AI vs not<br>*(both after 5 years of tamoxifen)*	Ingle 2008[9]	4 years	5,170	0.68 (0.55–0.83)††	Not reported
	Extended AI<br>*(both after 5 or more years of endocrine therapy including AI)*	Goss 2016[10]<br>Mamounas 2019[11]	10 years‡‡<br>7 years§§	1,918<br>3,966	0.66 (0.48-0.91)††<br>0.85 (0.73–1.00)‡	Not reported<br>Not reported
**Radiotherapy**						
After breast-conserving surgery	Partial *vs* whole breast RT	Vicini 2019[12]	10 years	4,125¶¶	1.12 (0.98-1.29)‡	1.22 (0.94–1.58)
	Boost *vs* not after whole breast RT	Bartelink 2015[13]	20 years	5,318	0.97 (0.88-1.08)§	0.65 (0.52–0.81)

Abbreviations: RR rate ratio; CI confidence interval; *vs* versus; HER2 human epidermal growth factor 2; ER oestrogen receptor; PR progesterone receptor; path CR pathological complete response; AI aromatase inhibitor; RT radiotherapy

*The time period following diagnosis that RRs relate to. In most studies, this starts soon after time of diagnosis, as randomisation took place soon after diagnosis. For studies, where randomisation did not take place until several years after diagnosis, both time from diagnosis to randomisation and time studied following randomisation are given in footnotes.

†Odds ratio for pathological complete response to platinum chemotherapy versus platinum-free chemotherapy was 1.96, 95% CI 1.46-2.62.

‡ Breast cancer recurrence, second primary malignancy or death. For capecitabine, a reduction in overall mortality was reported (RR 0.59, 95% CI 0.39-0.90, P=0.01) so it is likely that it did, in fact reduce breast cancer mortality, although this was not reported specifically.

§ Recurrence of ipsilateral invasive breast tumor, recurrence of ipsilateral locoregional invasive disease, distant disease recurrence, contralateral invasive breast cancer, or death from any cause.

¶ First occurrence of invasive ipsilateral tumour recurrence, invasive contralateral breast cancer, local or regional invasive recurrence, distant recurrence, or death from any cause.

** Invasive recurrence of breast cancer, invasive contralateral breast cancer, second (non-breast) cancer, or death without recurrence or a second breast cancer.

†† Invasive recurrence of breast cancer or invasive contralateral breast cancer.

‡‡Women randomised after around 5 years of AI preceded by 5 years of tamoxifen. Time-period studied is 10 years starting at randomisation.

§§ Women received about 5 years of AI or of tamoxifen→AI before randomisation. Time-period studied is 7 years starting at randomisation.

¶¶ 76% of patients had invasive breast cancer and 24% had ductal carcinoma in situ.

References for Supplemental Table 6

1 Poggio F, Bruzzone M, Ceppi M, Ponde NF, La Valle G, Del Mastro L, et al. Platinum-based neoadjuvant chemotherapy in triple-negative breast cancer: a systematic review and meta-analysis. Ann Oncol 2018; 29: 1497-508. https://doi.org/10.1093/annonc/mdy127

2 Masuda N, Lee SJ, Ohtani S, Im YH, Lee ES, Yokota I, et al. Adjuvant Capecitabine for Breast Cancer after Preoperative Chemotherapy. N Eng J Med; 2017;376:2147-59. https://doi.org/10.1056/NEJMoa1612645

3 Piccart M, Procter M, Fumagalli D, de Azambuja E, Clark E, Ewer MS, et al. Adjuvant pertuzumab and trastuzumab in early HER2-positive breast cancer in the APHINITY trial: 6 years' follow-up. JCO 2021;39:1448-57. https://www.nejm.org/doi/full/10.1056/NEJMoa1703643

4 von Minckwitz G, Huang CS, Mano MS, Loibl S, Mamounas EP, Untch M, et al. Trastuzumab Emtansine for Residual Invasive HER2-Positive Breast Cancer. N Eng J Med 2019;380:617-28. https://doi.org/10.1056/NEJMoa1814017

5 Martin M, Holmes FA, Ejlertsen B, Delaloge S, May B, Iwata H, et al. Neratinib after trastuzumab-based adjuvant therapy in HER2-positive breast cancer (ExteNET): 5-year analysis of a randomised, double-blind, placebo-controlled, phase 3 trial. Lancet Oncol 2017;18:1688-700. https://doi.org/10.1016/S1470-2045(17)30717-9

6 Early Breast Cancer Trialists' Collaborative Group. . Long-term outcomes for neoadjuvant versus adjuvant chemotherapy in early breast cancer: meta-analysis of individual patient data from ten randomised trials. Lancet Oncol 2018; 19: 27-39. https://doi.org/10.1016/S1470-2045(17)30777-5

7 Francis PA, Pagani O, Fleming GF, Walley BA, Colleoni M, Láng I, et al. Tailoring adjuvant endocrine therapy for premenopausal breast cancer. N Eng J Med 2018;12;379:122-37. https://doi.org/10.1056/NEJMoa1803164

8 Early Breast Cancer Trialists' Collaborative Group. Aromatase inhibitors versus tamoxifen in premenopausal women with estrogen receptor positive early stage breast cancer treated with ovarian suppression: patient-level meta-analysis of 7,030 women in four randomised trials. Lancet Oncol 2022. https://doi.org/10.1016/S1470-2045(21)00758-0

9 Ingle JN, Tu D, Pater JL, Muss HB, Martino S, Robert NJ, et al. Intent-to-treat analysis of the placebo-controlled trial of letrozole for extended adjuvant therapy in early breast cancer: NCIC CTG MA.17. Ann Oncol 2008;19:877-82. https://doi.org/10.1093/annonc/mdm566

10 Goss PE, Ingle JN, Pritchard KI, Robert NJ, Muss H, Gralow J, et al. Extending Aromatase-Inhibitor Adjuvant Therapy to 10 Years. N Engl J Med 2016;375:209-19. https://www.nejm.org/doi/full/10.1056/NEJMoa1604700

11 Mamounas EP, Bandos H, Lembersky BC, Jeong JH, Geyer CE, Rastogi P, et al. Use of letrozole after aromatase inhibitor-based therapy in postmenopausal breast cancer (NRG Oncology/NSABP B-42): a randomised, double-blind, placebo-controlled, phase 3 trial. Lancet Oncol 2019;20:88-99. https://doi.org/10.1016/S1470-2045(18)30621-1

12 Vicini FA, Cecchini RS, White JR, Arthur DW, Julian TB, Rabinovitch RA, et al. Long-term primary results of accelerated partial breast irradiation after breast-conserving surgery for early-stage breast cancer: a randomised, phase 3, equivalence trial. Lancet 2019;394:2155-64. https://doi.org/10.1016/S0140-6736(19)32514-0

13 Bartelink H, Maingon P, Poortmans P, Weltens C, Fourquet A, Jager J, et al. Whole-breast irradiation with or without a boost for patients treated with breast-conserving surgery for early breast cancer: 20-year follow-up of a randomised phase 3 trial. Lancet Oncol 2015;16:47-56. https://doi.org/10.1016/S1470-2045(14)71156-8

Supplemental Table 7. Rate ratios for the effects of adjuvant and neoadjuvant breast cancer treatments on individual causes of non-breast-cancer mortality

**Treatment type**<br>*Patient group*	**Treatment comparison**	**Reference**	**Time-period studied***	**Number of women**	**Number of individual causes assessed**	**Table/ Figure/ Page**	**Cause of death**	**Rate ratio (95% confidence interval) or numbers of deaths and p-value**
**Chemotherapy**								
All women	Anthracycline *vs* no chemotherapy†	EBCTCG 2012[1]	10 years	8,575	NS	P442	Heart disease<br>Acute myeloid leukaemia	1.61 (1.00-2.22)<br>8 vs 0 deaths, p=0.004‡
	Taxane+anthracycline *vs* anthracycline§	EBCTCG 2012[1]	8 years	44,251	NS	P442	Acute myeloid leukaemia	11.00 (1.42-85.17)‡
ER-PR- HER2-	Platinum (neoadjuvant)	Poggio 2018[2]	<1 year (path CR)	2,109	NS	-	Not reported	-
HER2- residual cancer after neoadjuvant chemotherapy	Capecitabine *vs* not	Masuda 2017[3]	4 years	910	NS	-	Not reported	-
**Anti HER2 therapy** *(HER2 positive cancer)*								
All women	Trastuzumab *vs* not	EBCTCG 2021[4]	10 years	13,864	9	Appendix P19	None sig¶	-
High risk	Pertuzumab *vs* not	Piccart 2021[5]	6 years	4,769	4	Table 3	None sig‡¶	-
Residual cancer after neoadjuvant chemo and anti HER2 therapy	Trastuzumab emtansine *vs* trastuzumab	von Minckwitz 2019[6]	3 years	1,486	1	P624	None sig‡¶	-
ER+ after adjuvant trastuzumab	Neratinib *vs* not	Martin 2017[7]	5 years	2,840	1	Table 3	None sig‡¶	-
**Neoadjuvant timing of chemotherapy**								
Neoadjuvant versus adjuvant chemotherapy		EBCTCG 2018[8]	15 years	4756	0	-	Not reported	
**Endocrine therapy in ER+ disease**								-
Any age	Tamoxifen *vs* not<br>*5 years*	EBCTCG 2011a[9]	15 years	10,645	6	Table 1	None sig¶	-
	Extended tamoxifen<br>*10 vs 5 years***	Davies 2013[10]	10 years	12,894	5	Table 2	None sig¶	-

Continued on next page

Continued from previous page

**Treatment category** *Patient group*	**Treatment comparison**	**Reference**	**Time-period studied***	**Number of women**	**Number of individual causes assessed**	**Table/ Figure/ Page**	**Cause of death**	**Rate ratio (95% confidence interval) or numbers of deaths and p-value**
**Endocrine therapy in ER+ disease**								
Pre-menopausal	Ovarian suppression *vs* not (both with tamoxifen) *5 years*	Francis 2018[11]	8 years	2,033	6	Tables S2, S3	None sig‡¶	
	AI *vs* tamoxifen (both with ovarian suppression) *3 or 5 years*	EBCTCG 2022[12]	10 years	7,030	21	Appendix P16	Cancers other than breast	2.44 (1.19-5.01)
Post-menopausal	AI *vs* tamoxifen *5 years*	EBCTCG 2015a[13]	10 years	9,885	6	Appendix P41	None sig¶	
	Tamoxifen→AI *vs* tamoxifen *5 years total*	EBCTCG 2015a[13]	8 years††	11,798	6	Appendix P43	None sig¶	
	AI vs not (both after 5 years of tamoxifen)	Ingle 2008[14]	4 years	5,170	3	Table 3	None sig‡¶	
	Extended AI *(both after 5 or more years of endocrine therapy including AI)*	Goss 2016[15]	10 years ‡‡	1,918	3	P214	None sig‡¶	
		Mamounas 2019[16]	7 years ¶¶	3,966	12	Table S2	None sig‡¶	
**Bisphosphonate**								
Women of all ages	Bisphosphonate *vs* not <1-*5 years*	EBCTCG 2015b[17]	10 years	18,766	0	-	Not reported	
**Radiotherapy** *Any surgery, target or nodes*	Radiotherapy *vs* not§§	EBCTCG 2017[18]	20 years	40,781	29	Table 2	Heart disease	1.30 (1.15-1.46)
						Table 2	Lung cancer§§	1.64 (1.22-2.21)
						Table S4	Oesophageal cancer	2.51 (1.08-5.72)
						Table S4	Thrombo-embolism	2.10 (1.11-3.90)

Abbreviations: *vs* versus; NS not specified; HER2 human epidermal growth factor 2; ER oestrogen receptor; PR progesterone receptor; path CR pathological complete response; AI aromatase inhibitor
* The time period following diagnosis that RRs relate to. In most studies, this starts soon after time of diagnosis, as randomisation took place soon after diagnosis. For studies where randomisation did not take place until several years after diagnosis, both time from diagnosis to randomisation and time studied following randomisation are given in footnotes.
†Rate ratio is for any anthracycline chemotherapy *versus* no chemotherapy.
‡See Supplemental Table 3 for calculation of risk ratios, confidence intervals and p-values based on data published in the references.
§Rate ratio is for taxane+anthracycline *vs* the same or more non-taxane chemotherapy.
¶None of the causes reported was significantly increased in women randomised to the more intensive treatment compared with the less intensive treatment.
** Individual causes of death were not presented in the meta-analysis in Table 2 (Ibrahim 2017). Results from the largest trial to report individual causes of death (ATLAS) are included here.
†† Treatments diverged 2-3 years after diagnosis. Time-period studied is 8 years starting at 2 years after diagnosis.
‡‡Women randomised after around 5 years of AI preceded by 5 years of tamoxifen. Time-period studied is 10 years starting at randomisation.

¶¶ Women received about 5 years of AI or of tamoxifen→AI before randomisation. Time-period studied is 7 years starting at randomisation.
§§Lung cancer mortality over all time periods was increased, but the excess was only 10+ years after diagnosis. Separate lung cancer mortality RRs for years 0-9 and 10+ were unavailable. Lung cancer incidence rate ratios during years 0-9 and 10+ were 1.08 (0.76-1.53) and 2.10 (1.48-2.98) respectively. These RRs are likely to be similar to mortality RRs due to the poor survival from lung cancer

References for Supplemental Table 7

1 Early Breast Cancer Trialists' Collaborative Group. Comparisons between different polychemotherapy regimens for early breast cancer: meta-analyses of long-term outcome among 100,000 women in 123 randomised trials. Lancet 2012;379:432-44. https://doi.org/10.1016/S0140-6736(11)61625-5

2 Poggio F, Bruzzone M, Ceppi M, Ponde NF, La Valle G, Del Mastro L, et al. Platinum-based neoadjuvant chemotherapy in triple-negative breast cancer: a systematic review and meta-analysis. Ann Oncol 2018; 29: 1497-508. https://doi.org/10.1093/annonc/mdy127

3 Masuda N, Lee SJ, Ohtani S, Im YH, Lee ES, Yokota I, et al. Adjuvant Capecitabine for Breast Cancer after Preoperative Chemotherapy. N Eng J Med; 2017;376:2147-59. https://doi.org/10.1056/NEJMoa1612645

4 Early Breast Cancer Trialists' Collaborative Group. Trastuzumab for early-stage, HER2-positive breast cancer: a meta-analysis of 13,864 women in seven randomised trials. Lancet Oncol 2021;22:1139-50. https://doi.org/10.1016/S1470-2045(21)00288-6

5 Piccart M, Procter M, Fumagalli D, de Azambuja E, Clark E, Ewer MS, et al. Adjuvant pertuzumab and trastuzumab in early HER2-positive breast cancer in the APHINITY trial: 6 years' follow-up. JCO 2021;39:1448-57. https://www.nejm.org/doi/full/10.1056/NEJMoa1703643

6 von Minckwitz G, Huang CS, Mano MS, Loibl S, Mamounas EP, Untch M, et al. Trastuzumab Emtansine for Residual Invasive HER2-Positive Breast Cancer. N Eng J Med 2019;380:617-28. https://doi.org/10.1056/NEJMoa1814017

7 Martin M, Holmes FA, Ejlertsen B, Delaloge S, May B, Iwata H, et al. Neratinib after trastuzumab-based adjuvant therapy in HER2-positive breast cancer (ExteNET): 5-year analysis of a randomised, double-blind, placebo-controlled, phase 3 trial. Lancet Oncol 2017;18:1688-700. https://doi.org/10.1016/S1470-2045(17)30717-9

8 Early Breast Cancer Trialists' Collaborative Group. Long-term outcomes for neoadjuvant versus adjuvant chemotherapy in early breast cancer: meta-analysis of individual patient data from ten randomised trials. Lancet Oncol 2018; 19: 27-39. https://doi.org/10.1016/S1470-2045(17)30777-5

9 Early Breast Cancer Trialists' Collaborative Group. Relevance of breast cancer hormone receptors and other factors to the efficacy of adjuvant tamoxifen: patient-level meta-analysis of randomised trials. Lancet 2011a;378:771-84. https://doi.org/10.1016/S0140-6736(11)60993-8

10 Davies C, Pan H, Godwin J, Gray R, Arriagada R, Raina V, et al. Long-term effects of continuing adjuvant tamoxifen to 10 years versus stopping at 5 years after diagnosis of oestrogen receptor-positive breast cancer: ATLAS, a randomised trial. Lancet 2013;381(9869):805-16. https://doi.org/10.1016/S0140-6736(12)61963-1

11 Francis PA, Pagani O, Fleming GF, Walley BA, Colleoni M, Láng I, et al. Tailoring adjuvant endocrine therapy for premenopausal breast cancer. N Eng J Med 2018;12;379:122-37. https://doi.org/10.1056/NEJMoa1803164

12 Early Breast Cancer Trialists' Collaborative Group. Aromatase inhibitors versus tamoxifen in premenopausal women with estrogen receptor positive early stage breast cancer treated with ovarian suppression: patient-level meta-analysis of 7,030 women in four randomised trials. Lancet Oncol 2022. https://doi.org/10.1016/S1470-2045(21)00758-0

13 Early Breast Cancer Trialists' Collaborative Group. Aromatase inhibitors versus tamoxifen in early breast cancer: patient-level meta-analysis of the randomised trials. Lancet 2015a;386:1341-52. https://doi.org/10.1016/S0140-6736(15)61074-1

14 Ingle JN, Tu D, Pater JL, Muss HB, Martino S, Robert NJ, et al. Intent-to-treat analysis of the placebo-controlled trial of letrozole for extended adjuvant therapy in early breast cancer: NCIC CTG MA.17. Ann Oncol 2008;19:877-82. https://doi.org/10.1093/annonc/mdm566

15 Goss PE, Ingle JN, Pritchard KI, Robert NJ, Muss H, Gralow J, et al. Extending Aromatase-Inhibitor Adjuvant Therapy to 10 Years. N Engl J Med 2016;375:209-19. https://www.nejm.org/doi/full/10.1056/NEJMoa1604700

16 Mamounas EP, Bandos H, Lembersky BC, Jeong JH, Geyer CE, Rastogi P, et al. Use of letrozole after aromatase inhibitor-based therapy in postmenopausal breast cancer (NRG Oncology/NSABP B-42): a randomised, double-blind, placebo-controlled, phase 3 trial. Lancet Oncol 2019;20:88-99. https://doi.org/10.1016/S1470-2045(18)30621-1

17 Early Breast Cancer Trialists' Collaborative Group. Adjuvant bisphosphonate treatment in early breast cancer: meta-analyses of individual patient data from randomised trials. Lancet 2015b;386:1353-61. https://doi.org/10.1016/S0140-6736(15)60908-4

18 Early Breast Cancer Trialists' Collaborative Group. Estimating the Risks of Breast Cancer Radiotherapy: Evidence From Modern Radiation Doses to the Lungs and Heart and From Previous Randomized Trials. J Clin Oncol 2017;35:1641-49. https://doi.org/10.1200/JCO.2016.72.0722

Supplemental Table 8. Epidemiological studies in which dose-response relationships for radiation-related diseases have been derived. Studies are of women with breast cancer unless otherwise indicated

(a) Heart disease (incidence)

Reference	Endpoint	No of cases*
Darby 2013[1]	Major coronary events	963
Lorenzen 2020[2]	Major coronary events	531
Jacobse 2019[3]	Ischaemic heart disease	183
Van den Bogaard 2017[4]	Acute coronary events	30

*Studies are ordered according to the number of cases.

(b) Lung cancer (incidence and mortality)

Reference	No of cases*
EBCTCG 2017[5]	475 (includes published data meta-analysis of all studies below)
Gilbert 2003[6]†	146
Grantzau 2014[7]	105
Inskip 1994[8]	61
Prochazka 2005[9]	70

*Studies are ordered according to the number of cases.
†Radiotherapy was for breast cancer in all studies except Gilbert 2003 where radiotherapy was for Hodgkin lymphoma.

(c) Oesophageal cancer (incidence)

Reference	No of cases
Morton 2012[10]	240 (based on dose to site of oesophageal cancer)
Journy 2020[11]	156

The dose-response relationship based on whole oesophagus dose was used in Table 3 because it is based on median oesophagus dose, which is assessable for patients being considered for breast cancer radiotherapy

(d) Thromboembolism

There are no available dose-response relationships. The mechanism by which radiation may cause thromboembolism is unknown.

References for Supplemental Table 8

1 Darby SC, Ewertz M, McGale P, Bennet AM, Blom-Goldman U, Bronnum D et al. Risk of ischemic heart disease in women after radiotherapy for breast cancer. N Engl J Med 2013; 368:987-98. https://doi.org/10.1056/NEJMoa1209825

2 Lorenzen EL, Rehammer JC, Jensen MB, Ewertz M, Brink C. Radiation-induced risk of ischaemic heart disease following breast cancer radiotherapy in Denmark 1977-2005. Radioth Oncol 2020; 152: 103-10. https://doi.org/10.1016/j.radonc.2020.08.007

3 Jacobse JN, Duane FK, Boekel NB, Schaapveld M, Hauptmann M, Hooning M, et al. Radiation Dose-Response for Risk of Myocardial Infarction in Breast Cancer Survivors. Int J Radiat Onc Biol Phys 2019; 103:595-604. https://doi.org/10.1016/j.ijrobp.2018.10.025

4 Van den Bogaard VAB, Ta BDP, Van der Schaaf A, Bouma AB, Middag AMH, Bantema-Joppe EJ et al. Validation and modification of a prediction model for acute cardiac events in patients with breast cancer treated with radiotherapy based on three-dimensional dose distributions to cardiac substructures. J Clin Oncol 2017; 35:1171-78. https://doi.org/10.1200/JCO.2016.69.8480

5 Early Breast Cancer Trialists' Collaborative Group. Estimating the risks of breast cancer radiotherapy: Evidence from modern radiation doses to the lungs and heart and from previous randomised trials. J Clin Oncol 2017; 35:1641-9. https://doi.org/10.1200/JCO.2016.72.0722

6 Gilbert ES, Stovall M, Gospodarowicz M, Van Leeuwen FE, Andersson M, Glimelius B, et al. Lung cancer after treatment for Hodgkin's disease: Focus on radiation effects. Radiat Res 2003; 159:161-73. https://doi.org/10.1667/0033-7587(2003)159[0161:lcatfh]2.0.co;2

7 Grantzau T, Thomsen MS, Vaeth M, Overgaard J. Risk of second primary lung cancer in women after radiotherapy for breast cancer. Radioth Oncol 2014; 111:366-73. https://doi.org/10.1016/j.radonc.2014.05.004

8 Inskip PD, Stovall M, Flannery JT. Lung cancer risk and radiation dose among women treated for breast cancer. J Natl Cancer Inst 1994; 86:983-88. https://doi.org/10.1093/jnci/86.13.983

9 Prochazka M, Hall P, Gagliardi G, Granath F, Nilsson BN, Shields PG, at al. Ionizing radiation and tobacco use increases the risk of a subsequent lung carcinoma in women with breast cancer: Case-only design. J Clin Oncol 2005; 23:7467-74. https://doi.org/10.1200/JCO.2005.01.7335

10 Morton LM, Gilbert ES, Hall P, Andersson M, Joensuu H, Vaalavirta L, et al. Risk of treatment-related esophageal cancer among breast cancer survivors. Ann Oncol 2012; 23:3081-91. https://doi.org/10.1093/annonc/mds144

11 Journy N, Schonfeld SJ, Hauptmann M, Roberti S, Howell RM, Smith SA, et al. Dose-volume effects of breast cancer radiation therapy on the risk of second oesophageal cancer. Radioth Oncol 2020; 151:33-9. https://doi.org/10.1016/j.radonc.2020.07.022
